# Chromatin alternates between A and B compartments at kilobase scale for subgenic organization

**DOI:** 10.1038/s41467-023-38429-1

**Published:** 2023-06-06

**Authors:** Hannah L. Harris, Huiya Gu, Moshe Olshansky, Ailun Wang, Irene Farabella, Yossi Eliaz, Achyuth Kalluchi, Akshay Krishna, Mozes Jacobs, Gesine Cauer, Melanie Pham, Suhas S. P. Rao, Olga Dudchenko, Arina Omer, Kiana Mohajeri, Sungjae Kim, Michael H. Nichols, Eric S. Davis, Dimos Gkountaroulis, Devika Udupa, Aviva Presser Aiden, Victor G. Corces, Douglas H. Phanstiel, William Stafford Noble, Guy Nir, Michele Di Pierro, Jeong-Sun Seo, Michael E. Talkowski, Erez Lieberman Aiden, M. Jordan Rowley

**Affiliations:** 1 Department of Genetics, Cell Biology and Anatomy, University of Nebraska Medical Center, Omaha, NE USA; 2 Center for Genome Architecture, Department of Molecular and Human Genetics, Baylor College of Medicine, Houston, TX USA; 3 Computational Biology and Clinical Informatics, Baker Heart and Diabetes Institute, Melbourne, VIC Australia; 4 Center for Theoretical Biological Physics, Northeastern University, Boston, MA USA; 5 CNAG-CRG, Centre for Genomic Regulation (CRG), Barcelona Institute of Science and Technology (BISB), 17 08028 Barcelona, Spain; 6 Integrative Nuclear Architecture Laboratory, Center for Human Technologies, Istituto Italiano di Tecnologia, Genova, Italy; 7 Paul G. Allen School of Computer Science & Engineering, University of Washington, Seattle, WA USA; 8 Department of Genome Sciences, University of Washington, Seattle, WA USA; 9 Department of Structural Biology, Stanford University School of Medicine, Stanford, CA 94305 USA; 10 Massachusetts General Hospital, Boston, MA USA; 11 Macrogen Inc, Seoul, Republic of Korea; 12 Department of Human Genetics, Emory University School of Medicine, Atlanta, GA USA; 13 Curriculum in Bioinformatics and Computational Biology, University of North Carolina at Chapel Hill, Chapel Hill, NC USA; 14 Thurston Arthritis Research Center, University of North Carolina, Chapel Hill, NC USA; 15 Department of Cell Biology and Physiology, University of North Carolina, Chapel Hill, NC USA; 16 Department of Biochemistry and Molecular Biology, University of Texas Medical Branch, Galveston, TX USA; 17 Department of Physics, Northeastern University, Boston, MA USA; 18 Asian Genome Institute, Seoul National University Bundang Hospital, Gyeonggi-do, Republic of Korea; 19 Department of Neurology, Harvard Medical School, Boston, MA USA; 20 Program in Medical Population Genetics and Stanley Center for Psychiatric Research, Broad Institute of MIT and Harvard, Cambridge, MA USA; 21 Center for Theoretical Biological Physics, Rice University, Houston, TX USA

**Keywords:** Nuclear organization, Epigenomics, Genome informatics, Gene regulation

## Abstract

Nuclear compartments are prominent features of 3D chromatin organization, but sequencing depth limitations have impeded investigation at ultra fine-scale. CTCF loops are generally studied at a finer scale, but the impact of looping on proximal interactions remains enigmatic. Here, we critically examine nuclear compartments and CTCF loop-proximal interactions using a combination of in situ Hi-C at unparalleled depth, algorithm development, and biophysical modeling. Producing a large Hi-C map with 33 billion contacts in conjunction with an algorithm for performing principal component analysis on sparse, super massive matrices (POSSUMM), we resolve compartments to 500 bp. Our results demonstrate that essentially all active promoters and distal enhancers localize in the A compartment, even when flanking sequences do not. Furthermore, we find that the TSS and TTS of paused genes are often segregated into separate compartments. We then identify diffuse interactions that radiate from CTCF loop anchors, which correlate with strong enhancer-promoter interactions and proximal transcription. We also find that these diffuse interactions depend on CTCF’s RNA binding domains. In this work, we demonstrate features of fine-scale chromatin organization consistent with a revised model in which compartments are more precise than commonly thought while CTCF loops are more protracted.

## Abstract (web summary)

Ultra-deep mapping of genome organization uncovers precise nuclear compartments and diffuse CTCF loops. This work demonstrates that compartment domains segregate the 5′ and 3′ ends of genes and that CTCF loops create proximal structures.

## Introduction

The nucleus of the human genome is partitioned into distinct spatial compartments, such that stretches of active chromatin tend to lie in one compartment, called the A compartment, and stretches of inactive chromatin tend to lie in the other, called the B compartment^1^. Compartmentalization was identified using Hi-C, a method that relies on DNA-DNA proximity ligation to create maps reflecting the spatial arrangement of the genome^1^. Loci in the same spatial compartment exhibit relatively frequent contacts in a Hi-C map, even when they lie far apart along a chromosome or on entirely different chromosomes^1,2^. Accurate classification of the resulting genome-wide contact patterns requires a large number of contacts to be characterized at each locus^3^. As such, genome-wide compartment profiles in human cells are typically generated at resolutions ranging from 40 kb to 1 Mb^1,2,4^. Even recently published fine-scale maps using Micro-C did not investigate compartment eigenvector at <100 kb resolution^5,6^. This may be because extant compartment detection algorithms require operations, such as calculating principal eigenvectors^1^, which are computationally intractable when the underlying matrices have millions of rows and columns—high-resolution Hi-C matrices^3^. Indeed, fine-scale compartment analysis has been more feasible in organisms with smaller genomes, such as *Drosophila melanogaster*^7,8^.

Here, we construct an in situ Hi-C map in human lymphoblastoid cells spanning 42 billion read-pairs and 33 billion contacts. We combine this map with the creation of an algorithm dubbed POSSUMM, which greatly accelerates the calculation of the principal eigenvector and the largest eigenvalues of massive, sparse matrices containing millions of rows and billions of nonzero entries. Combining our ultra-deep map with POSSUMM, we find that it is possible to map the contents of the A and B compartments with 500 bp resolution, a 100-fold improvement in resolution. This resolution demonstrates fine-scale compartment organization, such that nearly all active promoters and enhancers locate in tiny A compartments, even when proximal regions are in B. We also detect discordant compartments on gene bodies, such that the 5′ and 3′ ends of genes often locate to distinct compartments. These sub-genic discordant compartments occur most frequently at large and at paused genes.

Finally, we show that when we classify loops based on their appearance, at fine resolution, it becomes possible to distinguish between loops that form by extrusion and those that form via non-extrusion mechanisms. This analysis reveals interactions proximal to CTCF loops that depend on CTCF’s RNA binding domains. Overall, this work reveals several fundamental principles of fine-scale 3D genome organization.

## Results

### Generation of an ultra-deep in situ Hi-C map in lymphoblastoid cells spanning 33 billion contacts

**Fig. 1:**
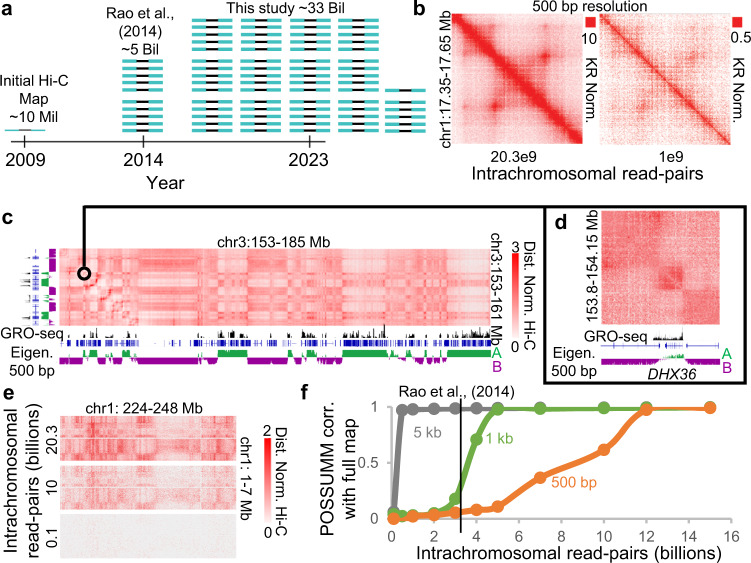
By combining ultra-deep Hi-C and POSSUMM, we generated a fine map of nuclear compartmentalization achieving 500 bp resolution. **a** Schematic representing the total mapped read-pairs in the current study compared to earlier published Hi-C studies. **b** Example locus showing Hi-C signal in 500 bp bins in our full map with 20.3 billion intrachromosomal read-pairs (left) and when read-pairs are subsampled to 1 billion (right). Scales are set to be proportional to sequencing depth. **c** Example of compartment interactions in a Hi-C map identified by the eigenvector (Eigen.) in 500 bp bins (bottom track). The black track displays transcription measured by GRO-seq. The black square represents the region shown in Fig. 1d. Scales represent distance normalized Hi-C. **d** Zoomed in view of a compartment domain. **e** Long-range Hi-C signal displaying how sequencing depth impacts the visibility of the long-range compartmental checkerboard pattern. **f** Correlation of the eigenvector in the full map compared to various sequencing depths. The black line indicates the number of intra-chromosomal read pairs in the published GM12878 dataset^2^. Source data are provided as a Source Data file.

We produced an ultra-deep Hi-C map of lymphoblastoid cells by sequencing over 42 billion PE150 read-pairs with over 150 individual Hi-C experiments. Experiments included a selection of three restriction enzymes, providing a digestion site every 75 bp on average (Supplementary Fig. 1a), and we obtained signal for 99.8% of non-repetitive 500 bp bins (Supplementary Fig. 1b). The resulting dataset is far deeper than any prior published Hi-C map and yielded 33 billion contacts after alignment, deduplication, and quality filtering (Supplementary Table 1). By comparison, the average published Hi-C map contains roughly 300 million contacts; 93% of Hi-C maps in the 4DNucleome database^9^ have less than 1 billion contacts (Supplementary Fig. 1c, Supplementary Table 2); and the widely used lymphoblastoid Hi-C map generated in Rao et al. contains 4.9 billion contacts (Fig. 1a).

We generated contact matrices at a series of resolutions, including as fine as 500 bp. These matrices greatly improved the visibility of features (Fig. 1b, browsable link: https://tinyurl.com/2ew48yof). Notably, this high coverage also enhanced the long-range plaid pattern indicative of compartments (Fig. 1c, Supplementary Fig. 1d, browsable link: https://tinyurl.com/2mthqtjk), as well as the corresponding compartment domains observed along the diagonal of the map (Fig. 1d, Supplementary Fig. 1e). Critically, because the number of contacts at every locus was greatly increased (Supplementary Fig. 1f-m), with an average of 22,000 contacts incident on each kilobase of the human genome, we were able to distinguish between loci in the A compartment and loci in the B compartment with much finer resolution (Fig. 1c).

### Development of PCA of Sparse, SUper Massive Matrices (POSSUMM), and its use to create a genome-wide compartment profile with 500 bp resolution

Extant methods for classifying loci into one compartment or the other typically rely on numerical linear algebra to calculate the principal eigenvector (called, in this context, the A/B compartment eigenvector) and the largest eigenvalues of correlation matrices associated with the Hi-C contact matrix. At 100 kb resolution, these matrices typically have thousands of rows and columns and millions of entries, making them tractable using extant numerical algorithms, such as those implemented by Homer^10^, Juicer^11^, and Cooler^12^. However, at kilobase resolution or beyond, these matrices have hundreds of thousands of rows and hundreds of billions to trillions of entries, making them intractable using the aforementioned tools. For example, computing an eigenvector for chr1 at 500 bp resolution entails generating a matrix with 250 billion entries and performing a calculation that is projected to require >4.6 TB of RAM (Supplementary Fig. 2a).

As such, we developed a method, POSSUMM, for calculating the principal eigenvector and the largest eigenvalues of a matrix. POSSUMM repeatedly multiplies a matrix with itself in order to calculate the principal eigenvector (Box 1). However, POSSUM does not explicitly calculate all of the intermediate matrices. Instead, it explicitly calculates only the tiny subset of intermediate values required to obtain the principal eigenvector using a Lanczos-like method making it vastly more efficient at calculating eigenvectors than current software (Box 1). In addition to our Hi-C matrices, we benchmarked POSSUMM’s calculation of eigenvectors on several other types of matrices available from https://sparse.tamu.edu, with sizes ranging from 2.2e4–2.3e8 rows/columns and from 2e6–3e9 non-zero entries. POSSUMM calculated the first four eigenvectors much faster and more efficiently than other methods (Box 1, Supplementary Fig. 2b, c, Supplementary Tables 3, 4). Importantly, due to memory efficiency, only POSSUMM was able to calculate eigenvectors for the largest matrices (Supplementary Fig. 2d, Supplementary Tables 3, 4). This demonstrates that POSSUMM enables the efficient calculation of eigenvectors in diverse types of massive matrices, including web-connectivity, protein databank, census data, gene regulatory network, internet traffic, and social network topology matrices, in addition to Hi-C data (Supplementary Table 3).

Using POSSUMM, we assigned loci to the A and B compartments at resolutions up to and including 500 bp (Fig. 1c). The calculation of the A/B compartment eigenvector at 500 bp resolution took only 2.5 min, and 23 GB of RAM (Supplementary Fig. 2a, d). In comparison, CscoreTool^13^, a non-PCA-based compartment caller, took 2.8 days and 62 GB of RAM to achieve similar compartment calls at 1 kb on chromosome 1 (Supplementary Fig. 2a, c). Because POSSUMM enables eigenvector calculation of massive matrices, we further tested it by calculating the compartment eigenvector at 500 bp resolution on the genome-wide (GW) matrix composed of inter-chromosomal interactions and has >38 trillion possible bin-pairs. Even with the extreme size of this matrix, POSSUMM took only 39 min and 77.65 GB of RAM to calculate the first four principal components (Supplementary Fig. 2a, Supplementary Table 3). The resultant compartment values from the principal eigenvector mostly matched those derived from individual chromosomes, but with some additional noise likely due to the overall lower inter-chromosomal signal characteristic of Hi-C maps (Supplementary Fig. 2e–g). For this reason, we use A and B compartments identified by POSSUMM on intra-chromosomal interactions, which accurately detects the segregation of active from inactive chromatin (Supplementary Fig. 2h–k). Importantly, the extreme sequencing depth was essential to identify compartments at 500 bp resolution due to signal sparsity at long distances for lower sequencing depths (Fig. 1e, f, Supplementary Fig. 2l-m). However, coarser resolution compartment analysis is also feasible by POSSUMM, and only 500 million intra-chromosomal contacts were necessary to identify compartments in 5 kb bins (Fig. 1f).

Box 1 PCA of Sparse, SUper Massive Matrices (POSSUMM) Overview of the POSSUMM algorithm for PCA analysis of massive matrices. POSSUMM calculates matrix-vector products using a sparse representation of the matrix, without explicitly computing the correlation matrix. By maintaining the sparsity of the matrix, POSSUMM makes eigenvector calculation more widely feasible, especially for data types in large sparse matrices such as those used for tracking social networks, web-connectivity, internet traffic, census data, and gene expression networks in addition to Hi-C maps. For genome-wide A/B compartment identification, POSSUMM’s matrix-vector product implementation enables eigenvector calculation at higher resolutions (smaller bins) that are prohibitive to other methods due to the massive size of the dense correlation matrix.
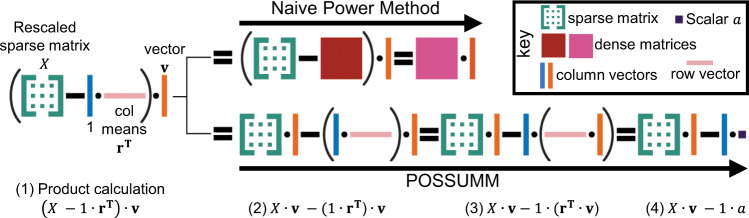

### The median compartment interval is 12.5 kb long

**Fig. 2:**
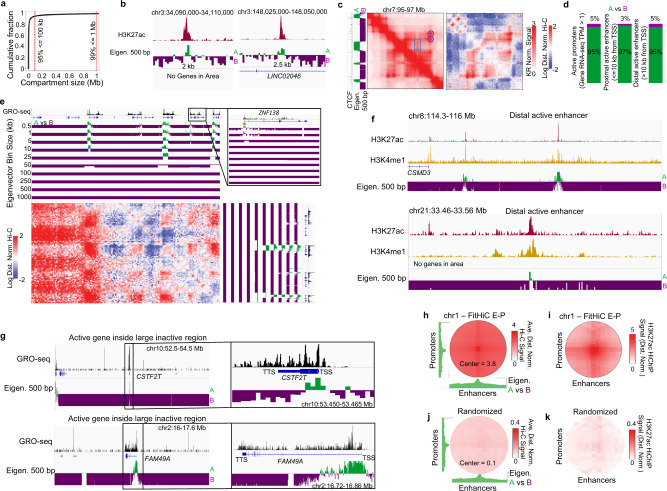
Nearly all active TSSs and enhancers localize to kilobase-scale A compartments. **a** Cumulative fraction of compartment domain sizes when identified at 500 bp resolution. **b** Examples of small compartment intervals. **c** Example of a compartmental interval smaller than an inside a CTCF loop. Observed and distance-normalized maps are shown to highlight the compartment interactions. Circles highlight loops that encompass the compartment interaction pattern indicated by a rectangle. CTCF ChIP-seq and eigenvector are shown on the side. **d** Percentage of active gene promoters, proximal enhancers, and distal enhancers assigned to A (green) or B (purple) compartment intervals when identified by the 500 bp compartment eigenvector. Source data are provided as a Source Data file. **e** Example of small compartment intervals only identifiable at high-resolution (red asterisks). Log transformed and distance normalized Hi-C map is shown alongside the eigenvector tracks at various bin sizes. **f** Examples of active enhancers denoted by H3K27ac and H3K4me1 signal localizing to the A compartment and surrounded by the B compartment. **g** Examples of active promoters denoted by GRO-seq signal localizing to the A compartment and surrounded by B compartment intervals. **h** Average eigenvector (green tracks) and Hi-C signal at promoters and enhancers identified by FitHiC. **i** Average H3K27ac HiChIP signal at those same loci. **j** Average Hi-C and **k** H3K27ac HiChIP signal at the same promoters and enhancers, but they are randomly assigned to each other. Color intensity scales on (**j**) and (**k**) are 10-fold lower to highlight the lack of signal even at this lower range.

We next used our fine map of nuclear compartments to examine the frequency with which loci alternate from one compartment to the other. Nearly 99% of compartment intervals were less than 1 Mb in size, and 95% were smaller than 100 kb (Fig. 2a). The median compartment interval was only 12.5 kb (Supplementary Fig. 3a), with some as small as 2 kb (Fig. 2b), and thousands of compartment intervals were no longer than 10 kb (Supplementary Fig. 3a, b). In comparison, the median size of CTCF loops in our map was 360 kb in length, demonstrating that compartment intervals can be smaller than individual loops (Fig. 2c).

We note that the size of the A/B compartments in this ultra-resolution map is smaller than that of previously annotated subcompartments. Originally annotated in GM12878 LCLs at 100 kb resolution^2^, subcompartments represent subclassifications of A/B compartments. We called subcompartments and were able to reach 10 kb resolution using Calder^14^, such that the subcompartments reflected different chromatin states (Supplementary Fig. 3c); bin sizes smaller than 10 kb met with memory limits likely stemming from the need to cluster the resultant massive matrix. We compared the high-resolution A/B compartments to subcompartments and found that subcompartments can indeed categorize A and B compartments further (Supplementary Fig. 3d). We also see that the different subcompartments correspond exceptionally well with the intensity of the eigenvector at fine-scale (Supplementary Fig. 3e). Thus, our data suggest that subcompartment calling represents a sub-classification of compartments as opposed to sub-scale features.

### Kilobase-scale compartment intervals frequently give rise to contact domains

It is well known that long compartment intervals often give rise to contact domains, i.e., genomic intervals in which all pairs of loci exhibit an enhanced frequency of contact among themselves^7,8,15–17^ (Fig. 1d). Such contact domains are referred to as compartment domains. We found that even short compartment intervals less than 5 kb frequently give rise to contact domains (Supplementary Fig. 4a), demonstrating that contiguous intervals of chromatin in the same compartment can form contact domains regardless of scale. We previously demonstrated that a proportion of TAD borders correspond to the edges of compartment domains which persist or strengthen upon loss of CTCF loops^8,17^. Using onTAD^18^ to define 14,400 unique TAD borders, we found that 19% of borders correspond to compartment domain borders, over half of which did not overlap CTCF loop anchors (Supplementary Fig. 4b). We then found that TADs corresponding to loops were slightly stronger than those corresponding to compartment domains (Supplementary Fig. 4c). Despite this strength difference, compartment domain borders are clearly visible by Hi-C, and we even see evidence of Hi-C domains where one border corresponds to a compartment border while the other is a CTCF loop border (Supplementary Fig. 4d). This further supports a model where domain and compartmental organization are not separate parts of a hierarchy, but rather, TADs consist of multiple distinct features at similar scales, including that of compartment domains and CTCF loop domains^15^.

### Essentially all active promoter and enhancer elements localize in the A compartment

Next, we compared our fine map of nuclear compartments to ENCODE’s catalog of regulatory elements in GM12878 cells. We examined active promoters (defined as 500 bp near the TSS, absence of repressive marks H3K27me3 or H3K9me3, and with >= 1 Reads Per Kilobase per Million [RPKM] gene expression in RNA-seq) and found that nearly all lie in the A compartment, with only 5% assigned to the B compartment (Fig. 2d — left). When examining active promoters assigned to the B compartment, we noticed that even these had higher values in the principal eigenvector compared to the surrounding regions (Supplementary Fig. 5a). Indeed, if we use a slightly more stringent threshold (assigning promoters to the B compartment only if the corresponding entry of the principal eigenvector is <−0.001), we find that only 233 (2.5%) of active promoters are assigned to the B compartment. Notably, the eigenvector from coarser bins placed most of the active promoters in the A compartment, however 10 kb, 100 kb, and 1 Mb resolutions resulted in an extra 62, 360, and 1270 active promoters to be assigned to the B compartment (Supplementary Fig. 5b). This is at least in part because the use of coarse resolutions leads to the averaging of interaction profiles from neighboring loci, such that a DNA element in the A compartment might be assigned to the B compartment if most of the flanking sequence was inactive (Fig. 2e, Supplementary Fig. 5c–h).

Similarly, we found that essentially all active proximal enhancers (defined by annotation in DenDB^19^, ≤10 kb from a TSS, and overlapping H3K27ac but not H3K27me3 and H3K9me3^20^) lie in the A compartment (Fig. 2d – middle). Moreover, essentially all active distal enhancers (DenDB^19^, >10 kb from a TSS, with H3K27ac, but not H3K27me3 or H3K9me3^20^) lie in the A compartment (Fig. 2d – right): only 5% were assigned to the B compartment. Many of these distal enhancer elements represent small islands of A compartment chromatin in a sea of inactive, B compartment chromatin (Fig. 2b,c,f). This demonstrates that individual DNA elements can escape a neighborhood that is overwhelmingly associated with one compartment to localize with a different compartment (Fig. 2b-g, Supplementary Fig. 5g, h). When coarser resolution compartment profiles are used, the number of active distal enhancers assigned to the B compartment increases up to 4.6-fold at 1 Mb resolution (Supplementary Fig. 5i). Again, this is at least in part because the use of coarse resolutions leads to the averaging of interaction profiles from neighboring loci (Supplementary Fig. 5g–j).

Taken together, we find that essentially all active regulatory elements, including both promoters and enhancers, lie in the A compartment, even when immediately neighboring sequences do not. We, therefore, asked whether enhancer-promoter connections have a similar Hi-C signal to compartmental interactions. We called FitHiC interactions on chr1, finding that promoters have significantly called interactions that connect them to three enhancers on average (Supplementary Fig. 5k). We then examined distance normalized Hi-C signal and found a spike at the enhancer-promoter connection (Fig. 2h). This corresponds to a spike in A compartmental eigenvector (Fig. 2h), which may be indicative of a relationship between the A compartment and enhancer-promoter signal. These interactions can also be seen in available H3K27ac HiChIP data in GM12878 cells^21^ (Fig. 2i). We found a similar signal for promoter-promoter and enhancer-enhancer connections, with the strongest H3K27ac HiChIP signal at enhancer-enhancer connections (Supplementary Fig. 5lm). To determine if these enrichments can be explained by the fact that both the enhancers and promoters locate to the A compartment, we took the same list of enhancers and randomly shuffled them among the promoters, thereby creating a randomized list of pseudo-connections using the same anchors. Plotting the Hi-C and H3K27ac HiChIP signal, we find no evidence of these enhancers and promoters forming specific interactions despite both anchors lying in the A compartment (Fig. 2j,k). Therefore, enhancer-promoter interactions are more specific than general A compartment association.

### The B compartment is largely characterized by an absence of commonly examined chromatin marks

Recently, it was proposed that chromatin features (i.e., TF binding sites) in the A compartment are drivers of compartmentalization^18^. The ability of small, isolated distal enhancers to interact within the A compartment supports this model; therefore, we next characterized genomic intervals in the B compartment defined at 500 bp resolution. Similar to the A compartment, we found that small B compartmental intervals exist and are often oppositely annotated as A at coarser resolutions (Supplementary Fig. 6a). However, compared to the A compartment, we saw fewer B compartmental bins with opposite calls at coarser resolution (Supplementary Fig. 6b). Intriguingly, we found that small B compartment intervals frequently do not correspond to repressive chromatin marks such as H3K27me3 or H3K9me3 (Supplementary Fig. 6a, c). Indeed, we noticed that many B compartment intervals and B-type subcompartments do not correspond to any commonly examined chromatin mark, being mostly composed of quiescent chromatin^22^ (Supplementary Fig. 2h,3c). To extend this analysis, we compiled a list of loci that had evidence of any commonly studied active and repressive marks (peak in any of the following ENCODE datasets: H2AZ, H3K4me1, H3K4me3, H3K27ac, H3K36me3, H3K28me2, H3K9ac, H4K20me1, RNAPII, ATAC-seq, EZH2, H3K27me3, or H3K9me3). This revealed that 51% of B compartmental bins do not have any of these commonly studied marks (Supplementary Fig. 6d). Although far from comprehensive, this absence of common marks in the B compartment lends some support to the proposal that sequences in A compartmental intervals may be the drivers of compartmentalization^23^. We note, however, that the B compartment highly overlaps with Lamin Associated Domains, even those called in a different cell line^24^ (Supplementary Fig. 6e), and therefore might contribute to their organization^25,26^.

### Many genes exhibit discordant compartmentalization, with the TSS in the A compartment and the TTS in the B compartment

**Fig. 3:**
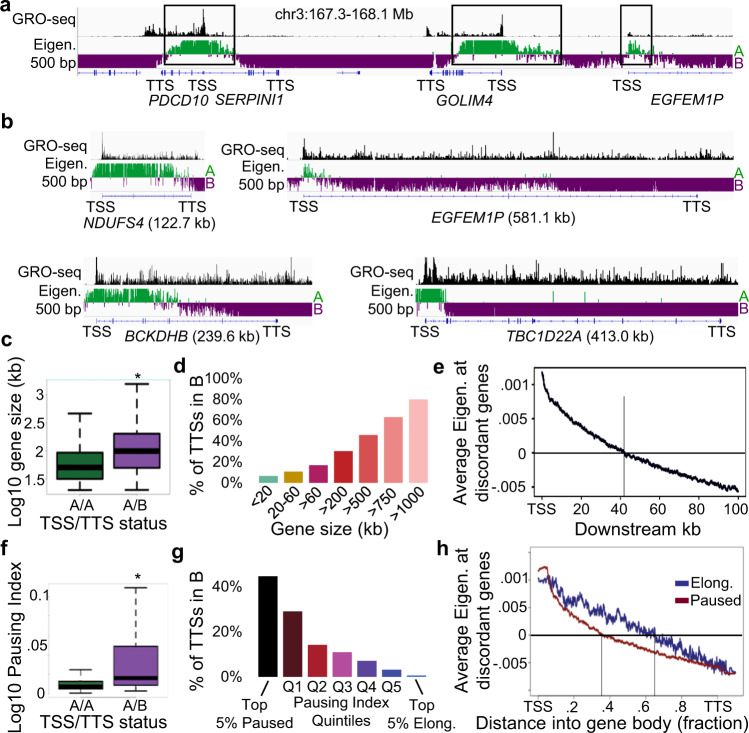
Many genes exhibit discordant compartmentalization. **a**, **b** Examples of genes of various sizes where the TSS is in the A compartment while the TTS is in the B compartment. GRO-seq signal is shown as an indicator of the gene’s transcription status. Black rectangles indicate regions of TSSs that reside in the A compartment. **c** Sizes of genes with concordant (labeled A/A & green) or discordant (labeled A/B & purple) compartments. * indicates *p* < 2.2e-16 two-sided Wilcoxon Rank Sum, *n* = 6021 (AA) and 510 (AB). Source data are provided as a Source Data file. **d** Percentage of TTSs that localize to the B compartment for genes of various sizes that have the TSS in the A compartment. Source data are provided as a Source Data file. **e** Average profile of the eigenvector at discordant genes. The vertical line indicates the distance where the average eigenvector value equals zero. **f** Pausing Index of genes with concordant (labeled A/A & green) or discordant (labeled A/B & purple) compartments. * indicates *p* < 2.2e-16 two-sided Wilcoxon Rank Sum, *n* = 6021 (AA) and 510 (AB). Source data are provided as a Source Data file. **g** Percentage of TTS that localize to the B compartment for genes with different pausing statuses and have the TSS in A. Source data are provided as a Source Data file. **h** Scaled average profiles of the compartment eigenvector for elongating (blue) or paused (red) discordant genes. The vertical line indicates the distance where the average eigenvector value equals zero.

When exploring the fine map of nuclear compartmentalization, we noticed many genes where the TSS and TTS localize to opposite compartments (Fig. 3a., Supplementary Fig. 7a,b, see also Figs. 1d, 2e,g), which we term as discordant. Approximately 8% of genes with the TSS in the A compartment are discordant (Supplementary Fig. 7c). Discordant compartments are more easily seen at large genes (Fig. 3b, c). Indeed, very few small genes (<20 kb) are discordant, while the majority of large genes (>750 kb) are discordant (Fig. 3d). Using the average profile at discordant genes, we find that the eigenvector decreases sharply after the TSS followed by ~2% decrease every 1 kb downstream, with an average crossing threshold into the B compartment at ~42 kb (Fig. 3e).

We next asked if genes with discordant compartments (i.e., the TSS was in compartment A, but the TTS was in compartment B) could be explained by different chromatin marks at the TSS vs. TTS. We examined chromatin marks at the TTS in active genes larger than 20 kb, comparing genes with concordant vs. discordant compartments. Notably, genes with discordant compartments cannot be explained by heterochromatin overlapping the TTS as they generally lack repressive marks (Supplementary Fig. 7c, d). Looking at the TSS, concordant and discordant compartment genes have similar marks and likely cannot be explained by differences at the TSS (Supplementary Fig. 7d, e). Instead, we found that diminished levels of active marks at the TTS, specifically RNAPII, H3K4me1, and H3K36me3, were correlated with the presence of discordant compartments (Supplementary Fig. 7d, e).

We noticed that discordant genes have lower expression levels (Supplementary Fig. 7f); therefore, we sought to determine if discordant compartmentalization was associated with transcriptional pausing as measured by GRO-Seq. By examining genes longer than 20 kb, we found that long elongating genes are more likely to exhibit concordant compartmentalization, whereas long paused genes were more likely to exhibit discordant compartmentalization (Fig. 3f, g). Indeed, average profiles across discordant genes revealed that elongating genes have a larger portion of the gene body in the A compartment (30% more on average) (Fig. 3h). Analysis of subcompartments showed similar results in regards to gene size and correlation with transcriptional pausing (Supplementary Fig. 7g).

Taken together, these data support a model where an active TSS localizes to the A compartment but brings with it only a small portion of the gene body, depending on the elongation status.

### 3D modeling helps delineate compartment domains

**Fig. 4:**
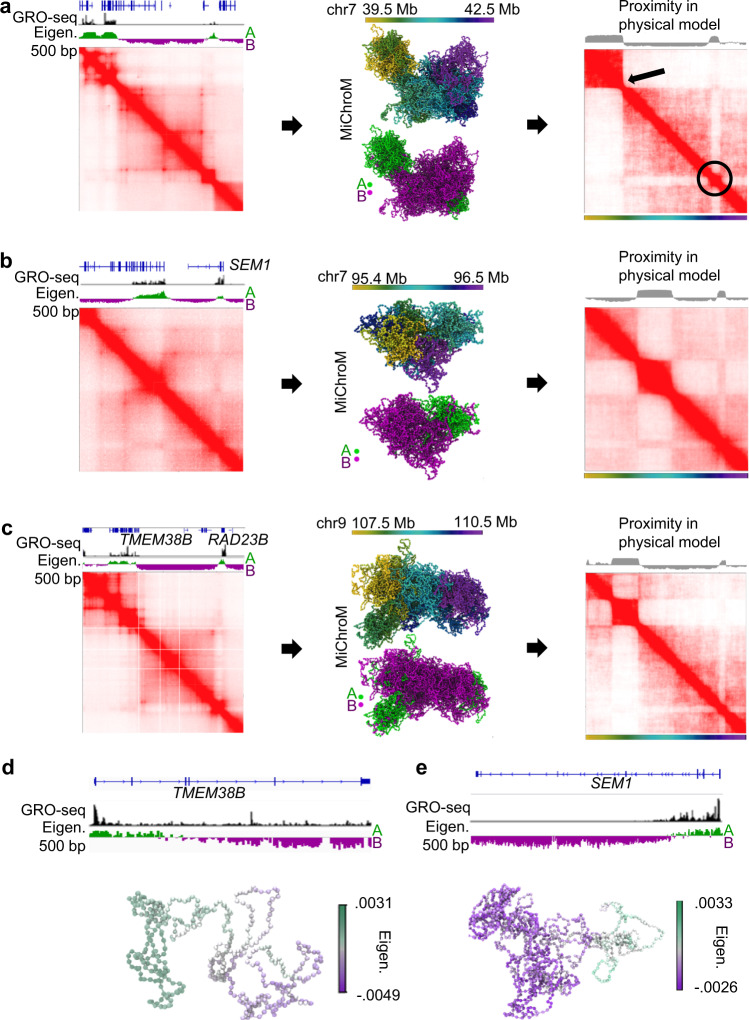
Computational modeling of chromatin segments with kilobase-scale compartments. **a–c** Examples of simulated chromatin segments for chr7: 39.5–42.5 Mb, chr7: 95.4–96.5 Mb and chr9: 107.5–110.5 Mb. Each segment is shown with the experimental Hi-C map (left) and a representation of the structures (middle) that were used to build the simulated distance map (right) at 1 kb resolution. The circle and line indicate compartmental features near the diagonal captured by the simulation. **d**, **e** Examples modeling genes with discordant compartments are shown for *TMEM38B* in chromosome 9 and *SEM1* in chromosome 7.

Because ultra-deep Hi-C reveals compartmental patterns at the kilobase scale, we next modeled the ensemble of 3D genomic structures associated with those patterns. Using the MiChroM energy landscape model, we performed molecular dynamics physical simulations for several segments of chromatin ranging from 1 Mb to 3 Mb (Fig. 4); thus, minimizing the risk of spurious boundary effects. To characterize the structural organization of fine-scale compartmentalization, these molecular dynamics simulations used nucleosome-resolution modeling of the chromatin fiber, thus significantly finer than the smallest feature under investigation (see Methods).

First, we trained the energy-function parameters to recapitulate the compartmental pattern seen at 1 kb resolution in a 3 Mb region of chromosome 7 (Fig. 4a). Once trained, we used these parameters to predict the 3D structural ensembles of a 1.1 Mb, 3 Mb, and 2.5 Mb region on chromosomes 7, 9, and 4, respectively (Fig. 4a–c middle, Supplementary Fig. 8a–e). To determine how well the ensembles of 3D structures reflect Hi-C contacts, we then generate 2D distance matrices for the nucleosomes within the ensemble structure (Fig. 4a–c, right, Supplementary Fig. 8e). Comparing experimental Hi-C maps to the MiChroM modeled distance maps (Fig. 4a–c, Supplementary Fig. 8d) reveals that the learned physical model accurately reflects the fine compartmentalization patterns near the diagonal (Supplementary Fig. 8a), which are often masked by CTCF loops (Fig. 4a), a feature outside our prediction. We then examined how this physical model depicts sub-genic discordant compartments for the ensemble structures of *TMEM38B* and *SEM1* (Fig. 4d, e). Physical simulations of discordant genes display extended structures due to the intra-genic transition between A and B compartments (Fig. 4d, Supplementary Fig. 8f, g). This is also supported by the slightly larger distributions of the radius of gyration (R_g_) for these genes in comparison with the same-sized regions completely in compartment A or B (Supplementary Fig. 8h). Our model suggests that discordant genes likely have extended structures compared to their non-discordant counterparts (Supplementary Fig. 8i). Altogether, these results reveal that compartmental simulations can distinguish near-diagonal compartment domains from loop domains.

### Loci with ambiguous Hi-C compartment definitions have high cellular heterogeneity

**Fig. 5:**
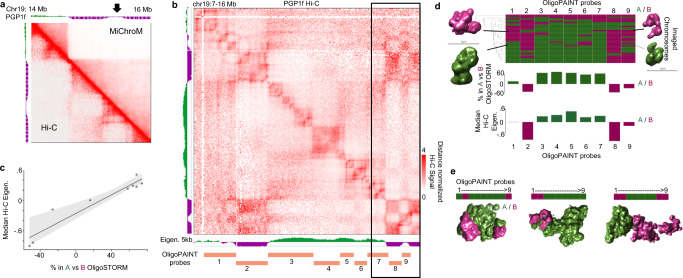
Ambiguous Hi-C compartment intervals have high heterogeneity. **a** Hi-C map compared to MiChroM in PGP1f cells. Arrow indicates a locus with ambiguous eigenvector values, which MiChroM had difficulty predicting. **b** Distance normalized Hi-C map of PGP1f cells, with the imaged segments denoted. Rectangle highlights the section modeled by MiChroM. **c** Median eigenvector within each imaged segment (y-axis) compared to the relative percent of images where that location was in A vs. B by OligoSTORM (x-axis). The line represents a linear fit, *R*^2^ = 0.91, while the shaded area is a fit encompassing all data points. **d** The imaging-based compartment status in single chromosomes. Heatmap represents the A or B designation of each imaged segment (columns) based on spatial and volumetric features of individual chromosomes in single cells (rows). On the sides are representative images of the corresponding genomic segments. Below are the percentage of individual chromosomes where the imaging reflects A vs. B compartment segments compared to the median eigenvector. Source data are provided as a Source Data file. **e** Representative images of the entire region colored by the A/B designation of each imaged segment. MichroM model parameters are included in Source Data.

Next, we examined compartments in 5 other published Hi-C maps with sufficient sequencing depth for POSSUMM to call compartments at 5 kb resolution^2,27^. We chose a 2 Mb region on chr19 that showed large differences in compartments (Supplementary Fig. 9a, b) and performed MiChroM modeling of this region in each cell type. In each, MichroM captured the organization attributed to compartments (Supplementary Fig. 9c, d). However, in some maps, we noticed that MichroM could not capture more ambiguous compartmental patterns, represented by the eigenvector near 0, for example, in PGP1f cells (Fig. 5a).

Unlike LCL, PGP1f cells are adherent, which enables super-resolution imaging^27^. This region of chr19 was previously imaged at super-resolution using the single-molecule localization microscopy method of OligoSTORM in PGP1f cells^27^. Nine chromosomal segments (CS1-9) ranging in size between 0.36 and 1.8 Mb (Fig. 5a) were imaged through sequential OligoSTORM in PGP1f cells, revealing correlations between three-dimensional structure and active and inactive chromatin (Fig. 5c, Supplementary Fig. 9e)^27^. Probed region CS9 corresponds to the region with ambiguous Hi-C eigenvector that MichroM was unable to model (Fig. 5b). We find the CS9 segment has the most heterogeneity in imaging, notably as much heterogeneity as CS1, which overlaps both A and B segments (Fig. 5d). These data indicate that ambiguous eigenvector segments, which are therefore difficult to model (e.g., Supplementary Fig. 9f), correspond to regions of high cellular heterogeneity. We also note that despite the heterogeneity in compartment status, small A and B genomic intervals can nevertheless be segregated into distinct physical locations in images of individual chromosomes (Fig. 5e)^27–30^.

### Loop extrusion forms diffuse loops

We next examined intense loops in our Hi-C dataset, identifying 32,970 loops. Ninety-one percent of these loops contained a CTCF-bound motif at both anchors, with a strong preference for the convergent orientation (Supplementary Fig. 10a). As previously noted, sequencing depth impacts the ability to identify total CTCF loops (Supplementary Fig. 10b–g) while the convergent orientation preference remains (Supplementary Fig. 10h). Interestingly, higher sequencing depth allowed detection of longer loops, plateauing at approximately 5 billion intra-chromosomal contacts (Supplementary Fig. 10i). Because of this plateau, we estimate that our ultra-resolution Hi-C data is able to capture the majority of CTCF loops, which also suggests an approximate upper limit for CTCF loop formation at ~3.4 Mb (Supplementary Fig. 10j).

**Fig. 6:**
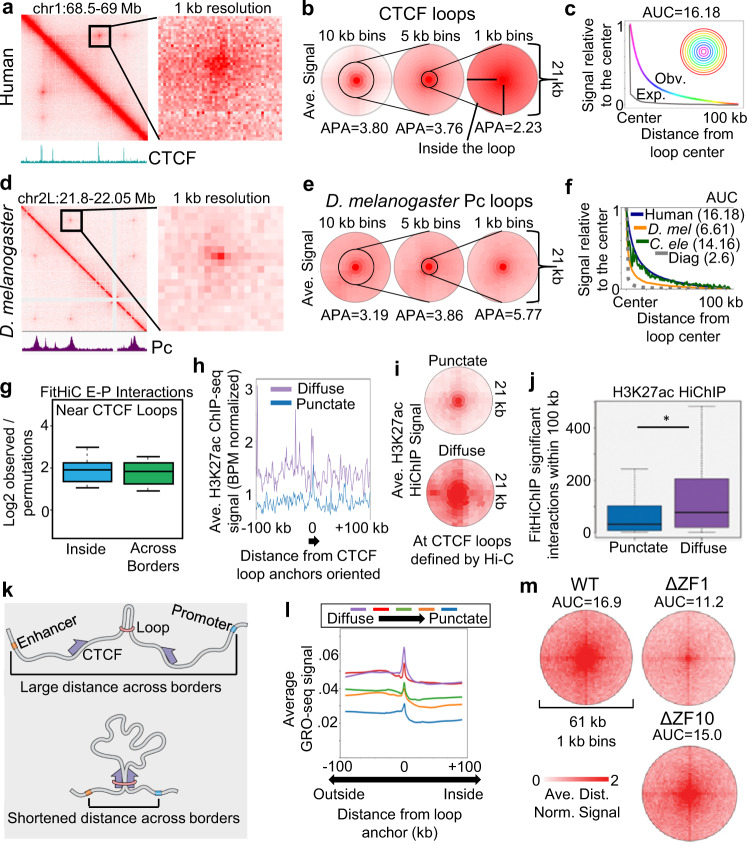
Diffuse CTCF loops are dependent on RNA-binding domains. **a** Example of broad signal enrichment near CTCF loops when binned at 1 kb. The CTCF ChIP-seq signal is shown below. **b** Average signal at CTCF loops when binned at 10, 5, or 1 kb, centered on convergent CTCF anchors. **c** Average Hi-C signal in 1 kb bins at each radial distance away from the CTCF loop anchors (rainbow). The average signal of the diagonal decay is shown for reference (gray) to estimate interactions due to polymeric distance. AUC = area under the curve. **d** Example of punctate signal enrichment at Pc loops in *D. melanogaster* when binned at 1 kb. The Pc ChIP-seq signal is shown below. **e** Average signal at *D. melanogaster* Pc loops when binned at 10, 5, or 1 kb. **f** Average Hi-C signal in 1 kb bins at each radial distance away from human CTCF loop anchors (blue) vs. *D. melanogaster* Pc loops (orange) and *C. elegans* X-chromosome loops (green). The average signal at the *C. elegans* Hi-C diagonal is shown for reference (gray). AUC = area under the curve. **g** Enrichment vs. random regions of Fit-Hi-C enhancer-promoter interactions within 100 kb of loops inside the loop (blue) or crossing over loop boundaries (green). Boxplots represent the median and the interquartile range (IQR), with whiskers representing 1.5*IQR. *n* = 18,948 EP FitHiC interactions, 2559 CTCF loops, and 10 permutations. Average H3K27ac ChIP-seq (**h**) and HiChIP (**i**) signal near diffuse vs. punctate CTCF loop anchors. **j** Number of H3K27ac HiChIP significant interactions determined by FitHiChIP near punctate (*n* = 1076) vs. diffuse (*n* = 1086) CTCF loop anchors. Boxplots represent the median and the interquartile range (IQR), with whiskers representing 1.5*IQR. * indicated *p* < 2.2e-16 Wilcoxon sum-rank test. **k** Diagram of how CTCF loops can shorten distances between enhancers (orange) and promoters (blue) even when both are located outside of the loop. **l** Average GRO-seq signal at CTCF loop anchors and neighboring loci for loops divided into five distinct diffuse categories. **m** Average Hi-C signal in WT (left), ΔZF1 (right), or ΔZF10 (bottom) CTCF mutants at CTCF loops. AUC area under the curve.

Interestingly, when we examined loops at 1 kb resolution, we noticed that the signal is diffuse (Fig. 6a, Supplementary Fig. 11a, browsable link: https://tinyurl.com/2f2sfp3a), indicative of frequent contacts proximal to the CTCF binding sites, which we will refer to as diffuse loop anchors (Fig. 6b). The elevated contact frequency decreases with distance from the corresponding anchors (Fig. 6c, rainbow) (a loss of signal of c.a. −6% from one bin to the next; i.e., 6%/kb compounding). Curiously, the rate of signal loss is much slower than the decay rate of the Hi-C diagonal (Fig. 6c, Supplementary Fig. 11b – expected) (c.a. −28%/kb), which is thought to reflect the properties of the chromatin polymer. We found that these diffuse structures are seen at a range of loop sizes (Supplementary Fig. 11c). While high sequencing depth is important to visualize diffuse structures at individual loops, these diffuse structures can be measured by metaplot analysis in maps with less sequencing depth (Supplementary Fig. 11d). However, even by metaplot analysis, maps with approximately 100 million intrachromosomal contacts or less impact the ability to measure the diffuse signal (Supplementary Fig. 11d). Turning to published data, we see evidence of diffuse CTCF loops in HFF cells by both in situ Hi-C and MicroC (Supplementary Fig. 11ef). We did see a sharper signal loss in Micro-C data, but this corresponds to a sharper diagonal decay (Supplementary Fig. 11g). Importantly, even by Micro-C, the rate of signal loss for loops was slower than the diagonal decay (Supplementary Fig. 11f), indicating that CTCF loops enrich the interactions of proximal loci.

We wondered whether this proximal signal (i.e., diffuse loops) was seen for loops in other species. We examined hundreds of loops observed in a published high-resolution Hi-C map from *Drosophila melanogaster* Kc167 cells at 1 kb resolution^7,31^ (Fig. 6d&e). Interestingly, the loops in *Drosophila* lose signal at a rate (c.a. −20%/kb) that matched the diagonal of the *Drosophila* Hi-C map (c.a. −23%/kb) and was more dramatic than the rate seen for human CTCF-mediated loops (Fig. 6f, Supplementary Fig. 11h&i). This suggests that CTCF loops create interactions between sequences bound by CTCF and adjacent sequences. However, in *Drosophila*, Polycomb complex (Pc) associated loops only create direct interactions between Pc-bound sequences.

Finally, we examined loops previously identified in *C. elegans*^32–34^. While the maps appeared noisier (Supplementary Fig. 11j), the loss of signal with distance was slower (c.a. −11%/kb) than at the diagonal (c.a. −24%/kb) (Fig. 6f, green vs. gray), and was more similar to the rate of loss seen for human CTCF-mediated loops than the one observed for *D. melanogaster* loops (Fig. 6f, Supplementary Fig. 11k).

Notably, the type of signal loss observed (diffuse vs. punctate) matched the putative mechanism by which the loops formed. CTCF-mediated loops in humans are bound by, and dependent on, the SMC complex and form by cohesin-mediated extrusion^35–38^. Indeed, after cohesin depletion, we detect a loss of both the central and proximal interactions (Supplementary Fig. 11l). The loops in *C. elegans* are bound by the SMC complex condensin, and we previously suggested that they are formed by condensin-mediated loop extrusion^32–34^. Indeed, the interactions between loop-adjacent sequences further support loop formation by extrusion in *C. elegans*. By contrast, *Drosophila* loops are much less likely to be bound by CTCF, cohesin, condensin, or other extrusion-associated proteins^7^. Instead, they are bound by the Polycomb complex, *Pc*, and may form by means other than extrusion^39–41^.

These findings suggest that the mechanism of loop formation influences whether loops will be punctate or diffuse, with extrusion-mediated loops forming diffuse peaks and compartmentalization-mediated loops forming more punctate features.

### Deletion of CTCF’s RNA binding domains leads to more punctate loops

We next examined promoter-enhancer FitHiC interactions where both the promoter and enhancer lie within 100 kb of a loop anchor. In some cases, these interactions lie entirely inside the loop, but in others, they cross the loop anchor. Both cases exhibited strongly enriched contact frequency as compared to enhancer-promoter interactions that are unrelated to CTCF loops, i.e., near permutated random sites (Fig. 6g). By contrast, in *Drosophila*, Fit-Hi-C interactions between promoters and enhancers do not extend as far away from the loop (Supplementary Fig. 12a). To further test the potential functional implication of diffuse CTCF loops, we categorized loops into more diffuse vs. more punctate (Supplementary Fig. 12b). We then examined H3K27ac ChIP-seq signal, a mark of active enhancers, and found that diffuse loops have more proximal H3K27ac within 100 kb compared to punctate loops (Fig. 6h). Using GM12878 H3K27ac HiChIP data^21^, we also found higher signal and more FitHiChIP^42^ significant interactions proximal to diffuse CTCF loops (Fig. 6i,j). We found that H3K27ac HiChIP interactions near diffuse loops are stronger both inside and outside the loop (Supplementary Fig. 12c). Thus, the diffuse CTCF loop signal corresponds to the enrichment of enhancer-promoter interactions nearby, even outside the loop (Fig. 6k).

The proximal signal did not correlate strongly with CTCF motif strength, CTCF ChIP-seq peak strength, or RAD21 ChIP-seq peak strength (Supplementary Fig. 12d–g). Instead, we found that more diffuse CTCF-mediated loops are associated with higher levels of transcription (Fig. 6l) and chromatin accessibility (Supplementary Fig. 12h) near the loop anchors. This suggests that nearby transcriptional activity could impact CTCF’s interaction with the nearby sequences and/or the loop extrusion process.

Recently it was shown that RNAs, including those found at active enhancers, are important for some of CTCF’s impact on chromatin organization^43^. The CTCF protein contains 11 zinc finger domains, and it was shown that ZF1 and ZF10 bind to RNA and that deletion of these two domains causes weakening of loops throughout the genome^44^. We performed aggregate peak analysis on the published Hi-C in ZF1 and ZF10 mutants^44^ using bullseye plots in order to explore the effect of these deletions on loop-proximal interactions. Interestingly, we found that loops appeared more punctate in both CTCF RNA binding mutants (Fig. 6m). This effect was especially pronounced in the ZF1 mutant. Another recent study performed Hi-C after the deletion of ZF8, which is not predicted to bind RNA^45^. While the sequencing depth was lower than what we found necessary to make conclusive claims at 1 kb resolution (see Supplementary Fig. 11d), metaplots failed to show a change in diffuse signal in the ZF8 mutant (Supplementary Fig. 12i).

Taken together, these findings are consistent with a model where CTCF’s RNA-binding domains and the presence of bound RNAs result in a more protracted diffuse loop and may enrich contacts among regulatory elements near the loop anchors.

## Discussion

By generating a Hi-C map with extraordinary sequencing depth (33 billion PE, or 9.9 terabases of uniquely mapped sequence), we create a fine-scale map of nuclear compartmentalization.

**Fig. 7:**
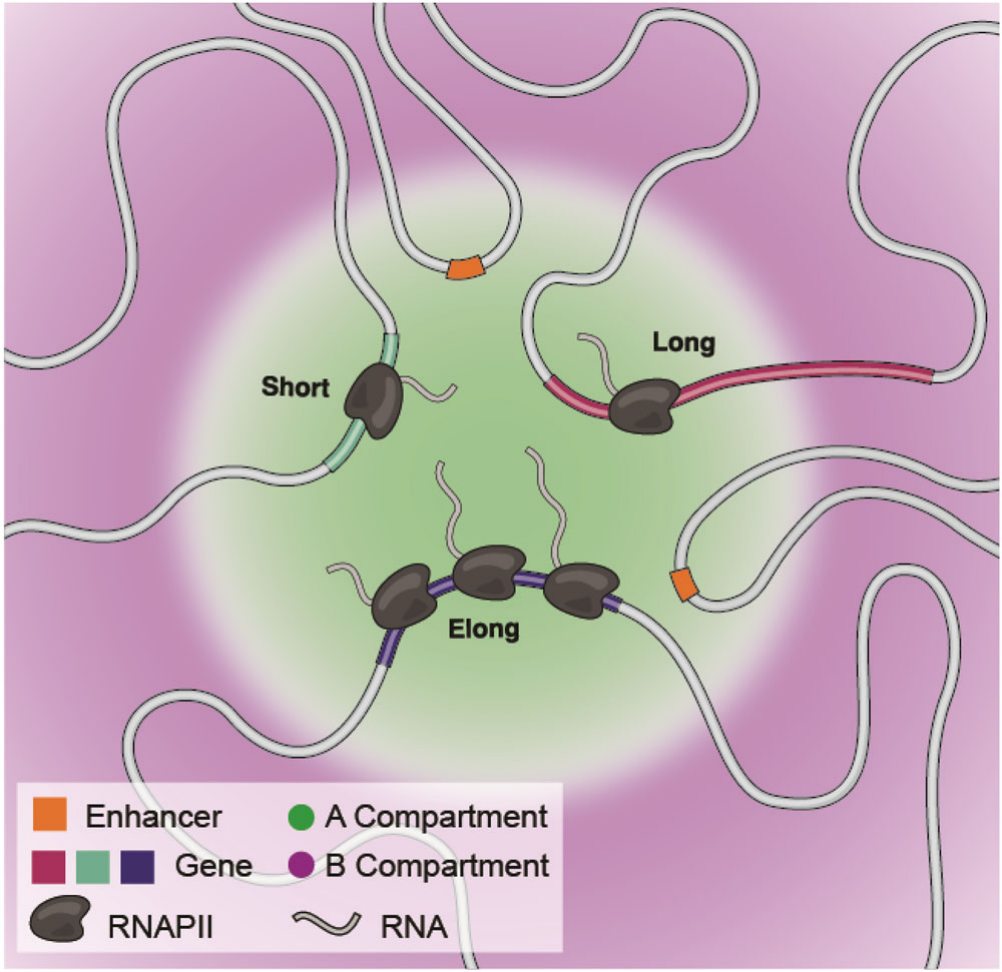
Sub-genic compartmentalization organizes the human genome. Diagram depicting localization of active enhancers and TSSs to the A compartment, while TTSs are oriented to the B compartment dependent on size and transcription elongation status.

Our findings demonstrate that compartment intervals and domains can be far smaller than previously appreciated. This contrasts with the common hierarchical model of chromatin organization in which compartments are multi-megabase features partitioned into TADs and loops^15,46–48^. Our results indicate that compartment intervals can be so small that active DNA elements will localize with the A compartment even when surrounded by inactive chromatin localizing in the B compartment (Fig. 7).

This study required approximately 150 Hi-C experiments, which were completed in 2018 as an ENCODE phase 4 pilot project exploring the generation of contact maps with much higher sequencing depths. Using Hi-C, we demonstrate that discordant sub-genic compartments and diffuse CTCF loop structures are present using the same basic methodology that led to the original identification and definition of the coarser features. POSSUMM achieved compartment calls at 5 kb in published Micro-C maps, much higher than the 100 kb resolution calls reported in the referenced publications^5,6^ (Supplementary Fig. 13, see Supplementary Discussion). Interestingly, we could denote compartments at 5 kb in their similarly deep Hi-C maps, suggesting that both Hi-C and Micro-C are suited for higher-resolution compartment identification. However, 5 kb is still an order of magnitude coarser than the 500 bp achieved in our current Hi-C study. Therefore, it will be valuable to characterize kilobase-sized compartments by Micro-C experiments sequenced to a similar depth used here. Indeed, during the review of this manuscript, a preprint reported the development of Region Capture Micro-C to achieve high sequencing depth at specific loci and identified micro-compartments within these specific regions^49^. While we cannot determine if these represent the same features, these findings are consistent in that small discrete loci can segregate into compartments at fine scale.

Strikingly, we find that essentially all distal enhancer elements lie in the A compartment. This contrasts with earlier work, using coarse-resolution maps of compartmentalization, which only report general enrichment of active distal enhancers in the A compartment rather than as a fundamental characteristic of active enhancers^50,51^. Similarly, many previous studies have reported a coarse enrichment of active genes in the A compartment^15^, yet we find that essentially all active promoters lie in the A compartment.

We also observe that the likelihood that a locus lies inside the A compartment declines as one moves away from the promoter along the gene body. Interestingly, we observe numerous genes with discordant compartmentalization, where the TSS and TTS tend to be in different compartments. Considering chromatin as a polymer, neighboring kilobase-sized A and B compartments likely cannot be located too far apart, which raises the question of whether compartments represent distinct physical locations. Such separation has been demonstrated via imaging^27–30^, albeit at genomic scales considerably larger than those achievable via POSSUMM and our ultra-deep Hi-C dataset. While speculative, this segregation could indicate phase-separated droplets^52–54^, which is supported by our physical modeling of chromatin phase separation. This suggests that the TSS and TTS of a gene with discordant compartmentalization might be physically proximal within the nucleus, in neighboring A and B droplets (Fig. 7). This may also explain the high levels of variability in genome organization detected by imaging approaches^27,55–57^, as transitions between A and B would not necessarily indicate large spatial movements. We should note, however, that recent evidence indicates that it is also possible that large spatial changes do occur after transcriptional activation^58^.

The finding that active promoters—specifically, active TSSs—are overwhelmingly localized in the A compartment, that TTS compartment status correlates with RNAPII levels at the TTS, and that genes with discordant compartmentalization tend to be transcriptionally paused is consistent with a model in which RNAPII drives localization to the A compartment. In support, recent results from DNA and RNA FISH showed dramatic changes in the conformation of large, activated genes^58^. In contrast, a recent RNAPII degradation study showed little effect on genome organization; however, these experiments did not achieve the sequencing depth required to perform the fine mapping of nuclear compartmentalization to resolve phenomena such as genes with discordant compartmentalization^59^. Alternatively, other components of the transcription complex that travel along the gene body during transcription elongation may mediate interactions that assign sequences to the A compartment. In future studies, it will be of great interest to examine how RNAPII and other components of the transcription complex impact genome organization at the TSS and TTS separately.

We note that our data represent averages within the cellular population, and as such, we cannot resolve where each finely resolved component lies during the transcriptional process itself. In the future, fine mapping of nuclear compartments in single cells will be needed to decipher these relationships. Moreover, our study did not attempt to study subcompartments or models with ≥3 distinct compartment states^2,50,60^, which will be an important topic for future work.

Our ultra-deep Hi-C map also helped identify interesting properties of chromatin loops. In particular, we observe that CTCF-mediated loops are highly diffuse or diffuse, more so than would be predicted based on polymer behavior alone. Interestingly, the enhanced loop-proximal signal is observed for loops that form by extrusion, such as loops in human^2,35–38^ and *C. elegans*^32–34^, but not for *Pc*-associated loops observed in *Drosophila*^7,31,40,41^.

In vitro studies have found that large chromatin complexes can impede looping factors^61,62^, and cohesin was shown to build up near transcriptionally active regions^63^. However, studies have also reported the independence of CTCF loops and transcription^59,64,65^, bringing the relationship between transcription and CTCF looping into question. Recently, it was shown that CTCF RNA-binding domains, ZF1 and ZF10, are important for looping^44^. Additionally, CTCF ZF1 mutations have been implicated in oncogenic transcription^66^. Our finding that diffuse loops are altered in CTCF RNA-binding mutants supports the argument that transcription can impact fine-scale chromatin organization in mammals^67^, as does the correlation between TTS compartmental domains and elongation status. Additionally, while nearly all active enhancers and promoters are in the A compartment, our findings indicate that other features likely drive enhancer-promoter specificity. Indeed, a class of regulatory tethering elements was recently proposed by high-resolution Micro-C data in D. *melanogaster*^68^. While we find that diffuse CTCF loops have more marks of active enhancers in their proximity, there could be a tradeoff between tethering elements and insulator function, as found in *D. melanogaster*^68^. Future work on how CTCF diffuse loops impact these two features will be important.

Our POSSUMM method, a numerical linear algebra algorithm for calculating principal eigenvectors, is now part of the Juicer pipeline for Hi-C analysis. Our power analyses suggest fine mapping of nuclear compartments at sub-kilobase resolution becomes possible for maps containing 7 billion contacts or more (See Supplemental Discussion). As sequencing costs continue to decline, we expect fine mapping of nuclear compartments will become increasingly common.

## Methods

### Inclusion and ethics

This research was approved by the Institutional Review Board of Mass General Brigham #2013P000323 as secondary analysis. The approval number C-0806-023-246 for the AK1 individual was assigned based on the Institutional Review Board of Seoul National University guidelines.

### Library preparation, initial processing, and quality metrics

Hi-C libraries were prepared according to the in-situ method^2^. In this method, cells were crosslinked in 1% v/v formaldehyde for ten minutes and quenched by adding 2.5 M glycine to a final concentration of 0.2 M for 5 min. Cells were pelleted by centrifugation at 300 *G* for 5 min at 4 °C and then washed with cold PBS. Cells were lysed with lysis buffer containing 10 mM Tri-HCl pH 8.0, 10 mM NaCl, 0.2% Igepal CA630 and protease inhibitors for 15 min on ice. Cells were centrifuged at 300 G and washed in that same buffer, and then resuspended in 50 µl 0.5% SDS for 5 min at 62 °C. Afterward, 145 µl of water and 25 µL of Trition X-100 were added and incubated at 37 °C for 15 min. Chromatin was digested overnight in MboI, MseI, or NlaIII in the corresponding buffer. Fragment overhangs were repaired and biotinylated using equal amounts of biotin-14-dATP, dCTP, dGTP, and dTTP in the presence of 40 units of DNA Polymerase I, Large (Klenow) Fragment at 37 °C for 1 h. Chromatin was ligated in 1x T4 DNA ligase buffer, Triton X-100, 0.12 mg BSA, and 2000 units T4 DNA ligase at room temperature for four hours. Proteins were digested, and chromatin decrosslinked in 0.72 mg/ml proteinase k, 1% SDS, and 0.5 M NaCl for 30 min at 55 °C, followed by 68 °C overnight. Libraries were sequenced and then processed using Illumina HiSeq 4000 software and JuicerTools v1.14.08^11^, in which we aligned to the hg19 genomeThe full map represents libraries prepared by digestion of various 4-cutter restriction enzymes, MboI, MseI, and NlaIII. To create a Hi-C megamap representing the average of lymphoblastoid cells, we pooled Hi-C from both male and female cell lines, GM12891 (RRID: CVCL_9630), GM12892 (RRID: CVCL_99631), GM18951 (RRID: CVCL_N804), GM18526 (RRID: CVCL_E124), GM13976 (RRID: CVCL_L266), GM13977 (RRID: CVCL_L267), GM11168 (RRID: CVCL_W113), GM19239 (RRID: CVCL_9634), AK1^69^, and GM12878 (RRID: CVCL_7526), and from six individuals. We found nearly as high reproducibility scores between these cell lines as we did between technical replicates (Tables S5, S6) and therefore combined them to form a mega-map of LCLs. Reproducibility scores were calculated by HiCRep v1.12.2 stratum adjusted correlation coefficient^70^. Subsampled Hi-C maps were created by uniform random selection of read-pairs from the 33.3 billion Hi-C dataset. We provide a script for subsampling Hi-C data at https://github.com/JRowleyLab/HiCSampler. Fragment size was performed by virtual digestion of the hg19 genome using MboI, MseI, and NlaIII. Estimation of the percent alignable rows was done by summing reads in each row and removing rows that were unmappable according to ENCODE’s publicly available hg19 mappability track: Index of /goldenPath/hg19/encodeDCC/wgEncodeMapability (ucsc.edu).

We used several metrics to evaluate the quality of the full 20.3 billion Hi-C map compared to subsampled Hi-C maps. First, as a simple Boolean metric, the number of bin pairs with at least one read was plotted as a fraction of the total number of possible bin pairs. This was done for bin pairs within a 1 Mb distance and all intra-chromosomal bin pairs. Non-mappable regions were excluded from analysis and identified by searching for rows and columns within the Hi-C matrix with no mappable read-pairs. Second, the noise estimates were calculated by taking the autocorrelation function (ACF) average, using a lag of 1, for each row within the matrix of distance-normalized Hi-C read-pairs. Noise values were then estimated by (ACF −1)*−1. To compare the 33.3 billion Hi-C map and subsampled maps, we calculated the ACF on a representative region of the matrix extending between chromosome 1: 1–10 Mb. We provide a script for noise estimation at https://github.com/JRowleyLab/HiCNoiseMeasurer.

UMAP clustering was performed using DNase, H2AZ, H3K27ac, H3K27me3, H3K36me3, H3K4me1, H3K4me3, H3K9ac, and H3K9me3 obtained by Avocado v0.1.0^71^. AA/AB clustering scores were obtained by taking each point in the A compartment, summing the UMAP cluster distances to the nearest 10 other points labeled as A, and dividing by the sum of distances to the nearest ten other points labeled B. As an alternative, we calculated the 5, 10, 50, 100, 500, and 1000 nearest neighbors using the ball tree algorithm in the python package scikit-learn v1.0.2^72^ and calculated the average number of neighbors that had opposite compartmental statuses. Logistic regression was performed using the python package statsmodels v0.13.2.

### Compartment analysis

Compartments were identified using the A/B eigenvector of the Hi-C matrix using POSSUMM and by CScoreTool v1.1^13^. POSSUMM can be downloaded from: https://github.com/aidenlab/EigenVector and is also now implemented in the ENCODE version of the Juicer pipeline: https://github.com/ENCODE-DCC/hic-pipeline. Subcompartments were identified by Calder v1.0^14^ at 10 kb; we tried higher resolution and alternative subcompartment callers but met with errors due to the extensive memory requirements necessary for the task.

### Introduction to PCA of Sparse, SUper Massive Matrices (POSSUMM)

Let $X$ be a matrix with column vectors ${X}^{(1)},\ldots,{X}^{\left(n\right)}$. Let ${Y}^{(i)}=({X}^{\left(i\right)}-{c}_{i})/{\sigma }_{i}\, 1\le i\le n$, where ${c}_{i}$ is the mean of ${X}_{i}$ and ${\sigma }_{i}$ is its standard deviation. Let $Y=({Y}^{\left(i\right)},\ldots,{Y}^{(n)})$ be an n x n matrix with column vectors. The correlation matrix of $X$ is defined as $A={Y}^{T}Y$ where ${Y}^{T}$ is transposed $Y$. Since $A$ is symmetric and positive semi-definite it has n real eigenvalues ${\lambda }_{1}\ge {\lambda }_{2}\ge \ldots \ge {\lambda }_{n}\ge 0$ and $n$ eigenvectors. ${v}_{1},\ldots,{v}_{n}$ where $A{v}_{i}={\lambda }_{i}{v}_{i}$.

We note that the so-called A/B compartment eigenvector is simply the eigenvector of $A$ corresponding to its largest eigenvalue, where $X$ is given by the Hi-C contact matrix. This is equivalent to the first principal component in Principal Component Analysis. In our case, $X$ is a large, sparse matrix containing millions of rows, millions of columns, and tens of billions of nonzero entries (dubbed a Sparse, SUper Massive Matrix).

Suppose we seek to calculate the largest eigenpairs, ${\lambda }_{i},{v}_{i}$ of $A$ in this case. Although $X$ is sparse, we note that both $Y$ and $A$ are dense matrices. Unfortunately, storing dense matrices with millions of rows and columns in memory is impossible. Hence we cannot use any method for calculating the eigenvectors of $A$ that would require us to explicitly calculate either $Y$ or $A$. Similarly, traditional sparse matrix methods for eigendecomposition are not usable here, again because $A$ - the correlation matrix we hope to analyze - is a dense matrix.

Therefore, to calculate eigenvectors for $A$, we began by implementing a method that makes it possible to calculate the matrix-vector product $A{{{{{\bf{v}}}}}}$ (where v is an arbitrary vector) using a sparse representation of $X$, i.e., without explicitly computing either $A$ or $Y$. See POSSUMM details below for a complete description.

Next, we note that there are many methods for calculating eigenvectors in which the input matrix only appears via a matrix-vector product. These include the Power and Lanczos methods and their many variants^73^. Thus, in principle, any of these methods - for which there are many implementations in Fortran, C, C++, Matlab, and R - can be combined with the sparse $A{{{{{\bf{v}}}}}}$ product calculation described above in order to calculate eigenpairs of $A$. In practice, methods combining these two approaches are not available.

To the best of our knowledge, the sole exception is a method in the R package *irlba*, which was released while this study was being performed. The details of this method are unpublished, but the method itself is available at https://cran.r-project.org/web/packages/irlba/index.html. However, *irlba* is implemented in R and cannot handle cases where $X$ has more than roughly two billion nonzero entries, which is exceeded in the present case. It also does not enable parallelization, which limits performance in highly demanding settings. We compare the Lanczos-like POSSUMM implementation to that of irlba v2.3.5 (Supplementary Table 3).

POSSUMM uses sparse $A{{{{{\bf{v}}}}}}$ product calculation, is memory-efficient, and enables parallelization via multi-threading.

### POSSUMM details

To identify compartments from sparse Hi-C matrices, we began by excluding all rows and columns with 0 variance. Let $X$ be a matrix with column vectors ${{{{{{\bf{X}}}}}}}^{\left({{{{{\bf{1}}}}}}\right)},{{\ldots }},{{{{{{\bf{X}}}}}}}^{\left({{{{{\bf{n}}}}}}\right)}$. Let (1) ${{{{{{\bf{Y}}}}}}}^{{{{{{\boldsymbol{(}}}}}}{{{{{\bf{i}}}}}}{{{{{\boldsymbol{)}}}}}}}=({{{{{{\bf{X}}}}}}}^{\left({{{{{\bf{i}}}}}}\right)}-{c}_{i})/{\sigma }_{i}\, 1\le i\le n$, where ${c}_{i}$ is the mean of ${{{{{{\bf{X}}}}}}}_{{{{{{\bf{i}}}}}}}$ and ${\sigma }_{i}$ is its standard deviation. Let $Y=\left({{{{{{\bf{Y}}}}}}}^{\left({{{{{\bf{i}}}}}}\right)},{{\ldots }},{{{{{{\bf{Y}}}}}}}^{\left({{{{{\bf{n}}}}}}\right)}\right)$ be an *n* × *n* matrix with column vectors. The correlation matrix of $X$ is (2) $A={Y}^{T}Y$ where ${Y}^{T}$ is transposed $Y$. Since A is symmetric and positive semi-definite it has *n* real eigenvalues ${\lambda }_{1}\ge {\lambda }_{2}\ge \ldots \ge {\lambda }_{n}\ge 0$ and $n$ eigenvectors. ${{{{{{\bf{v}}}}}}}_{{{{{{\bf{1}}}}}}}{{{{{\boldsymbol{,}}}}}}{{\ldots }}{{{{{\boldsymbol{,}}}}}}{{{{{{\bf{v}}}}}}}_{{{{{{\bf{n}}}}}}}$ where (3) $A{{{{{{\bf{v}}}}}}}_{{{{{{\bf{i}}}}}}}={\lambda }_{i}{{{{{{\bf{v}}}}}}}_{{{{{{\bf{i}}}}}}}$. These eigenvectors are a basis of *R*^*n*^ (i.e., a set of vectors that are independent and span the space) and if ${\lambda }_{i}\ne {\lambda }_{j}$ then ${{{{{{\bf{v}}}}}}}_{{{{{{\bf{i}}}}}}}\perp {{{{{{\bf{v}}}}}}}_{{{{{{\bf{j}}}}}}}$ (i.e., ${{{{{{\bf{v}}}}}}}_{{{{{{\bf{i}}}}}}}^{{{{{{\bf{T}}}}}}}{{{{{{\bf{v}}}}}}}_{{{{{{\bf{j}}}}}}}=0$). To compute ${{{{{{\bf{v}}}}}}}_{{{{{{\bf{1}}}}}}}$ using the power method (a.k.a power iterations), suppose that ${\lambda }_{1} > {\lambda }_{2}$ and let ${{{{{{\bf{x}}}}}}}_{{{{{{\bf{0}}}}}}}$ be any nonzero vector in *R*^*n*^, we define the recursive relation: (4) ${{{{{{\bf{x}}}}}}}_{{{{{{\bf{k}}}}}}{{{{{\boldsymbol{+}}}}}}{{{{{\bf{1}}}}}}}= A{{{{{{\bf{x}}}}}}}_{{{{{{\bf{k}}}}}}}={{A}^{k+1}{{{{{\bf{x}}}}}}}_{0}$. We can represent ${{{{{{\bf{x}}}}}}}_{{{{{{\bf{0}}}}}}}$ as (5) ${{{{{{\bf{x}}}}}}}_{{{{{{\bf{0}}}}}}}={a}_{1}{{{{{{\bf{v}}}}}}}_{{{{{{\bf{1}}}}}}}+\ldots+{a}_{n}{{{{{{\bf{v}}}}}}}_{{{{{{\bf{n}}}}}}}$ and therefore (6) ${A}^{k}{{{{{{\bf{x}}}}}}}_{{{{{{\bf{0}}}}}}}={a}_{1}{\lambda }_{1}^{k}{{{{{{\bf{v}}}}}}}_{{{{{{\bf{1}}}}}}}+\ldots+{a}_{n}{\lambda }_{n}^{k}{{{{{{\bf{v}}}}}}}_{{{{{{\bf{n}}}}}}}={\lambda }_{1}^{k}({a}_{1}{{{{{{\bf{v}}}}}}}_{{{{{{\bf{1}}}}}}}+{a}_{2}{\left(\frac{{\lambda }_{2}}{{\lambda }_{1}}\right)}^{k}{{{{{{\bf{v}}}}}}}_{{{{{{\bf{2}}}}}}}+\ldots+ {a}_{n}{\left(\frac{{\lambda }_{n}}{{\lambda }_{1}}\right)}^{k}{{{{{{\bf{v}}}}}}}_{{{{{{\bf{n}}}}}}})$. Once we have estimates of the eigenvector and the two largest eigenvalues, we can estimate the error given that (7) $\left|\left|{{{{{\bf{v}}}}}}-{{{{{{\bf{v}}}}}}}_{{{{{{\bf{1}}}}}}}\right|\right|\le \frac{\left|\left|A{{{{{\bf{v}}}}}}-{\lambda }_{1}{{{{{\bf{v}}}}}}\right|\right|}{\left|\left|{\lambda }_{1}-{\lambda }_{2}\right|\right|}$. To find an estimate of ${\lambda }_{2}$ we know that ${{{{{{\bf{v}}}}}}}_{{{{{{\bf{2}}}}}}}\perp {{{{{{\bf{v}}}}}}}_{{{{{{\bf{1}}}}}}}$ and ${{{{{{\bf{||v}}}}}}}_{{{{{{\bf{1}}}}}}}{||}=1$. Let ${{{{{{\bf{x}}}}}}}_{{{{{{\bf{0}}}}}}}$ be any vector and let (8) ${{{{{{\bf{x}}}}}}}_{{{{{{\bf{k}}}}}}{{{{{\boldsymbol{+}}}}}}{{{{{\bf{1}}}}}}}=A({{{{{{\bf{x}}}}}}}_{{{{{{\bf{k}}}}}}}-{c}_{k}{{{{{{\bf{v}}}}}}}_{{{{{{\bf{1}}}}}}})$ where ${c}_{k}={{{{{{\bf{v}}}}}}}_{{{{{{\bf{1}}}}}}}^{{{{{{\bf{T}}}}}}}{{{{{{\bf{x}}}}}}}_{{{{{{\bf{k}}}}}}}$ (and then $({{{{{\bf{x}}}}}}_{{{{{{\bf{k}}}}}}}-{c}_{k}{{{{{{\bf{v}}}}}}}_{{{{{{\bf{1}}}}}}})\perp {{{{{{\bf{v}}}}}}}_{{{{{{\bf{1}}}}}}}$). If (9) ${{\lambda_{2} }^{(k)}}=\left|\left|A{{{{{{\bf{x}}}}}}}_{{{{{{\bf{k}}}}}}}\right|\right|/|\left|{{{{{{\bf{x}}}}}}}_{{{{{{\bf{k}}}}}}}\right||$ using the same argument as before ${{\lambda_{2} }^{(k)}}\to {\lambda }_{2}$ as $k\to \infty$. This is true even if ${\lambda }_{2}\approx {\lambda }_{3}$ (${{{{{{\bf{x}}}}}}}_{{{{{{\bf{k}}}}}}}$ may not converge to ${{{{{{\bf{v}}}}}}}_{{{{{{\bf{2}}}}}}}$, but ${{\lambda }_{2}}^{(k)}$ wil converge to ${\lambda }_{2}$). In this way, we have an estimate of ${\lambda }_{1}$ and ${\lambda }_{2}$ and may estimate the error in ${{{{{\bf{v}}}}}}$. Since (10) $A={Y}^{T}Y,{Ax}={Y}^{T}\left({Yx}\right)={({\left({Yx}\right)}^{T}Y)}^{T}$, we do not need to compute A (which has the complexity of $O({n}^{3})$). We used two matrix-vector products at every iteration $Y$. Moreover, if $X$ is large a naïve multiplication of a vector by a matrix can still take a long time and storing $Y$ may require a large amount of memory. For example, to store human chr1 at 1 kb resolution (where $n\approx 250000$) 500 GB of RAM would be required just to store $Y$. With sparse implementation we recall that $Y=({{{{{{\bf{Y}}}}}}}^{\left({{{{{\bf{i}}}}}}\right)},\ldots,{{{{{{\bf{Y}}}}}}}^{\left({{{{{\bf{n}}}}}}\right)})$ where (11) ${{{{{{\bf{Y}}}}}}}^{\left({{{{{\bf{i}}}}}}\right)}=\frac{{{{{{{\bf{X}}}}}}}^{\left({{{{{\bf{i}}}}}}\right)}-{c}_{i}}{{\sigma }_{i}}=\frac{{{{{{{\bf{X}}}}}}}^{\left({{{{{\bf{i}}}}}}\right)}}{{\sigma }_{i}}-\frac{{c}_{i}}{{\sigma }_{i}}$ . While $\frac{{{{{{{\bf{X}}}}}}}^{\left({{{{{\bf{i}}}}}}\right)}}{{\sigma }_{i}}$ is sparse, $\frac{{{{{{{\bf{X}}}}}}}^{\left({{{{{\bf{i}}}}}}\right)}}{{\sigma }_{i}}-\frac{{c}_{i}}{{\sigma }_{i}}$ is not. In lieu of explicit computation, let $1={({{{{\mathrm{1,1}}}}},\ldots,1)}^{T}$ then (12) ${{{{{{\bf{Y}}}}}}}^{\left({{{{{\bf{i}}}}}}\right)}=\frac{{{{{{{\bf{X}}}}}}}^{\left({{{{{\bf{i}}}}}}\right)}}{{\sigma }_{i}}-\frac{{c}_{i}}{{\sigma }_{i}}1$ and then (13) ${Y={XS}-1\bullet r}^{T}$ where (14) $S=[1/{\sigma }_{1}\ddots 1/{\sigma }_{n}]$ and (15) $r={[{c}_{1}/{\sigma }_{1}{,\ldots,c}_{n}/{\sigma }_{n}]}^{T}$ and then (16) ${{Yx}=\left(X\bullet S\right)x-1\bullet r}^{T}\bullet x$. Let (17) $Z=X\bullet S$. Since (18) ${r}^{T}x={\sum }_{i=1}^{n}{{r}_{i}x}_{i},{Yx}={Zx}-({\sum }_{i=1}^{n}{{x}_{i}r}_{i})1$. Since Z is as sparse as X we can do everything with sparse matrices as (19) ${x}^{T}Y={x}^{T}Z-\left({x}^{T}1\right){r}^{T}={x}^{T}Z-({\sum }_{i=1}^{n}{x}_{i}){r}^{T}$. Now matrix-vector multiplication has a complexity of the number of nonzero elements in X (which never exceeds the number of contacts in the map). Projected time and memory usage were calculated by fitting a power decay curve, *R*^2^ of fit = 0.95 for time, and *R*^2^ of fit = 0.98 for memory usage.

After compartment calling, chromatin marks were profiled at features that overlap A or B compartments by overlapping with ChIP-seq peaks and using average signal profiles created by pyBigWig from the deepTools package^74^. ChIP-seq peaks and bigwig files were obtained from the ENCODE Roadmap Epigenomics project^75^. We filtered promoters with bivalent marks as active genes with twofold higher H3K27me3 or H3K9me3 signal compared to the average at promoters. Contiguous compartment domain sizes were calculated by requiring at least two consecutive bins to have the same sign in the eigenvector. We assigned genes to elongating, mid, and paused to create profiles of A compartmental status along genes. Elongation status was determined by RPKM GRO-seq signal within 250 bp of the TSS compared to the gene body, excluding 500 bp from the TSS. Differences between Promoter—Gene Body GRO-seq signal were ranked and placed into three equal categories considering only genes ≥20 kb in size.

### Loop analysis

Loops were identified by HiCCUPS included in JuicerTools v1.14.08^2^ or SIP v1.4^32^ at multiple resolutions. For HiCCUPS, we used parameters –m 2000 –r 500,1000,5000,10000 –f .05,.05.05.05. For SIP, we used an FDR 0.05 at each resolution with the parameters for resolutions of 500 bp; -d 15 –g 3.0; 1 kb: -d 17 –g 2.5; 5 kb: -d 6 –g 1.5; and 10 kb: -d 5 –g 1.3. Loops called by both methods were combined by placing all loops into 10 kb bins, and if HiCCUPS and SIP called the same loop within the 10 kb bin, then only one instance of this loop was kept. Loops in subsampled maps were overlapped with loops called in the full 20.3 billion maps if the loop was within ±25 kb of each other. Overlap of loops with CTCF was done using a published list of CTCF ChIP-seq peaks and motifs^2^. Central 1 kb bins were assigned to those where we could unambiguously assign a CTCF ChIP-seq peak to a unique bin at motifs in convergent orientation. Only loops with unambiguous CTCF assignment were used in loop-proximal, a.k.a. knot, analysis. *Drosophila* Pc loops were filtered for overlap between previously published identifications^31,40^. Bullseye plots were created using SIPMeta v1.3^32^^,^ and the rate of loss was calculated as the average at each Manhattan distance (ring) moving away from the central bin. These values were plotted as a ratio to the central bin’s signal. The central bin of loops called at AUC values was computed using Simpson’s rule. The percentage rate of change listed in the main text was calculated by averaging the number of kb between each 10% loss of signal. Loops were placed into five equally sized categories (quintiles) based on AUC values. AUC values between WT, ΔZF1, and ΔZF10 were normalized by the diagonal to account for differences in the expected decay. Hi-C for WT, ΔZF1, and ΔZF10 was obtained from GSE125595^44^, while WT vs. ΔZF8 was obtained from GSE153948^45^.

TADs were identified by the onTAD v1.4^18^ at 5 kb with default parameters. Note that we tried higher resolutions and alternate TAD callers but met with errors related to extensive memory usage. Overlap with compartment borders vs. CTCF loop anchors was performed by extending the TAD border 10 kb in either direction and taking the closest feature. TAD strength was directly derived by TAD. Fit-Hi-C^76^ interactions were identified in 1 kb bin-pairs with an FDR of 0.05.

Enhancer promoter interactions called in Hi-C were identified by FitHiC v2.07^76^ while H3K27ac HiChIP significant interactions were called by FitHiChIP^42^. Randomization of connections was done by taking the identified enhancer-promoter interactions on chr1 and shuffling the anchors randomly amongst themselves. H3K27ac HiChIP data in GM12878 cells were obtained from GSE101498^21^.

### Physical modeling of compartment structures

The physical model is based on the Minimal Chromatin Model (MiChroM) ^54^, but here we represent each nucleosome, a molecular assembly with a roughly cylindrical shape and a diameter of 10 nm, as a spherical particle with the same diameter. The distance between the centers of neighboring nucleosomes varies from 10 to 20 nm, i.e., the length of a straight linker DNA of about 50 bp. Similar to MiChroM, the energy function of the developed model consists of a term accounting for a generic homopolymer (*U*_*HP*_) as already described, together with interactions accounting for phase separation (*U*_*type-to-type*_) and a translational invariant term accounting for lengthwise compaction (*U*_*IC*_).

The energy function of the developed physical model takes the form: (20)

$${U}_{{MiChroM}-{nucleosome}}\left(\vec{r}\right)=	 {U}_{{HP}}\left(\vec{r}\right)+{U}_{{ty}{pe}-{to}-{type}}+{U}_{{IC}}={U}_{{HP}}\left(\vec{r}\right) \\ 	+\mathop{\sum}\limits_{\begin{array}{c}k > l\\ k,{l}\in {{{{{\rm{Types}}}}}}\end{array}}{\alpha }_{{kl}}\mathop{\sum}\limits_{\begin{array}{c}i\in \left\{{{{{{\rm{Nucleosome}}}}}}\; {{{{{\rm{of}}}}}}\; {{{{{\rm{Type}}}}}}k\right\}\\ j\in \left\{{{{{{\rm{Nucleosome}}}}}}\; {{{{{\rm{of}}}}}}\; {{{{{\rm{Type}}}}}}l\right\}\end{array}}f({r}_{{ij}}) \\ 	+\mathop{\sum }\limits_{d=10}^{{d}_{{cutoff}}}{\gamma }_{d}\mathop{\sum}\limits_{i}f({r}_{i,i+d})$$

In this energy function expression, *U*_*HP*_ indicates the homo-polymer potential of the chromatin fiber and consists of the following five terms, *U*_*FENE*_ (Finite Extensible Nonlinear Elastic potential), *U*_*Angle*_ (angle potential), *U*_*hc*_ (hard-core repulsive potential between nucleosome beads), *U*_*sc*_ (soft-core repulsive potential for non-bonded pairs of nucleosome beads) and *U*_*c*_ (confinement potential between the chromatin and a spherical wall). The functional form of individual terms above are the same as in MiChroM^54^, except in this case, the *U*_*FENE*_ is tuned to control the distance between neighboring nucleosomes consistently with the length of the linker DNA. Besides *U*_*HP*_, both *U*_*type-to-type*_ and *U*_*IC*_ are defined by the crosslinking probability (21) $f({r}_{{ij}})=\frac{1}{2}(1+{{\tanh }}[\mu ({r}_{c}-{r}_{{ij}})])$, with $\mu=1.79\sigma$, ${r}_{c}=3.43\sigma$ and $\sigma=10$ nm, and by the tunable coefficients $\alpha$ and $\gamma$ (values are provided in attachments).

Molecular dynamics simulations were performed following the protocol described in ref. ^54,77^. The production simulation of each chromatin segment presented in this work was carried out over eight replicas with $1.25\times {10}^{8}$ steps, storing a frame every $1\times {10}^{3}$ steps that generated a total of one million 3D structures. These structures were used to calculate the in silico Hi-C maps which are compared with the experimental ones. S

Parameters in the energy function were trained to reproduce the Hi-C contact map at 1 kb resolution on the 39.5–42.5 Mb region of chromosome 7. Once trained, the model is then used to predict the 3D structural ensembles of other regions with the sole input of the eigenvector for such regions. Then, the bead-to-bead distances in the ensemble 3D structures are used to generate, in silico, Hi-C maps at 1 kb resolution.

Differences between the MiChroM distance maps and Hi-C were quantified by taking the Pearson correlation compartmental matrix of each and calculating the mean of the squared differences (MSD), (22) ${Mean}({({{PearsonHiC}}_{{ij}}-{{PearsonSim}}_{{ij}})}^{2})$ between the two matrices. To estimate a null background, we took the MSD compared to Juicer’s expected matrix.

### OligoSTORM oligopaint analysis

The compartment classification of the OligoSTORM density map relative to 8.16 Mb region extending from chr19:7,400,000 (19p13.2) to chr19:15,560,000 (19p13.12) in PGP1f was obtained from previously published work^27^. Briefly, each of the nine chromosomal segments was clustered based on five structural and spatial measures (the distance score, the entanglement score, the surface area, the volume, and the sphericity score) in two major clusters. A feature vector was created for each homolog, which is a binary 1 × 9 vector encoding the cluster types of each chromosomal segment in the region. The resulting chromosome matrix was hierarchically clustered using the one-way unweighted pair group method with arithmetic means (UPGMA) based on the Jaccard similarity Jaccard index. POSSUMM was used to call compartments at 5 kb resolution in the Hi-C data. Compartment similarity matrices were calculated as abs (probeComp_i_ – probeComp_j_), where probeComp represents the median Hi-C eigenvector within the probed region or the relative A/B percentages of each probed region in OligoSTORM images.

### Comparison with other datasets

Hi-C read-pairs from CTCF ΔZF1, ΔZF1, and wild-type were downloaded from GSE125595^44^ and processed with juicer to the mm10 genome. Hi-C maps from the *D. melanogaster* dm6 genome and the *C. elegans* ce10 genome were obtained from our previously published work^31,32^. Hi-C maps used in our metric comparison are listed in Tables S2 and S7.

Enhancers were downloaded from DENdb^19^^,^ and active enhancers were defined as those that overlap with H3K27ac ChIP-seq peaks in GM12878. Histone modification ChIP-seq data were obtained from the ENCODE reference epigenome series ENCSR977QPF and RNAPII ChIP-seq peaks were combined from RNAPII, RNAPIISer2ph, and RNAPIISer5ph from ENCSR447YYN and ENCSR000DZK^20,78^, with overlapping peaks merged into a single peak. GRO-seq data from GM12878 was downloaded from GSM1480326^79^, and chromHMM states for GM12878 were downloaded from the Roadmap Epigenomics Project^75^.

### Study consent, sex, and/or gender considerations

Consent from individuals was obtained under the Institutional Review Board of Mass General Brigham #2013P000323 as a secondary analysis. The approval number C-0806-023-246 for the AK1 individual was assigned based on the Institutional Review Board of Seoul National University guidelines. This study combines samples from both male and female donors. Male lines include GM12891, GM11168, GM19239, AK1. Female lines include GM12892, GM18951, GM18526, GM13976, GM13977, GM12878. It was necessary to combine the datasets to obtain the resolution; therefore, we cannot compare and account for sex in this study. Technological limitations and costs prevent achieving a comparable resolution in each. We demonstrate the problems by comparing low-resolution maps to high-resolution maps in the supplementary figures. However, we provide the individual correlations between individual maps along with the gender of each Source Data. The individual maps are also available through the relevant accessions listed in Data Availability.

### Reporting summary

Further information on research design is available in the Nature Portfolio Reporting Summary linked to this article.

## Supplementary information

Supplementary Information

Peer Review File

Reporting Summary

## Data Availability

The human genome 19 (hg19) assembly is available from the NCBI accession GCF_000001405.13. The Hi-C data from public LCLs generated in this study have been deposited in the ENCODE database under accession codes: ENCSR261EVH for GM13977 (https://www.encodeproject.org/experiments/ENCSR261EVH/), ENCSR196MPD for GM11168 (https://www.encodeproject.org/experiments/ENCSR196MPD/), ENCSR118FFR for GM18951 (https://www.encodeproject.org/experiments/ENCSR118FFR/), ENCSR634FNY for GM13976 (https://www.encodeproject.org/experiments/ENCSR634FNY/), ENCSR410MDC for GM12878 (https://www.encodeproject.org/experiments/ENCSR410MDC/), ENCSR508EMN for AK1 (https://www.encodeproject.org/experiments/ENCSR508EMN/), ENCSR859YSL for GM12891 https://www.encodeproject.org/experiments/ENCSR859YSL/), ENCSR075VWI for GM12892 https://www.encodeproject.org/experiments/ENCSR075VWI/), ENCSR693CIM for GM18526 (https://www.encodeproject.org/experiments/ENCSR693CIM/), and ENCSR264SMC for GM19239 (https://www.encodeproject.org/experiments/ENCSR264SMC/). The combined signal matrix is browsable using juicebox.js by selecting Harris HL, Gu H. et al. from the Juicebox Archive menu. The previously published data used in this study are available in the ENCODE database under accessions ENCSR977QPF for histone modifications and DNase-seq, ENCSR447YYN for histone marks and RNAPIIser5ph, ENCSR000DZK for RNAPIISer2ph, and from the Gene Expression Omnibus (GEO) under accession GSM1480326 for GRO-seq, GSE123552 for PGP1f Hi-C, GSE125595 for Hi-C in ZF mutants, GSE101498 for H3K27ac HiChIP, GSE132640 for Hi-C in *C. elegans*, GSE80701 for Hi-C in *D. melanogaster* cells, and from the Roadmap Epigenomics Project (https://egg2.wustl.edu/roadmap/data/byFileType/chromhmmSegmentations/ChmmModels/coreMarks/jointModel/final/E116_15_coreMarks_dense.bed.gz) for chromHMM states. Chromatin 3D structures are deposited in the Nucleome Data Bank https://ndb.rice.edu/Data and can be downloaded by selecting Harris_etal_NatComm_2023 from the dropdown menu^80^. Source data for Fig. 1g, 2d, 3c, 3f, 3g, 5d, cell lines by sex and/or gender, and MiChroM model parameters are included in the Source Data file. Source data are provided with this paper.

## Source data

Source Data

## Code availability

Our programs for subsampling, noise estimation, and eigenvector calculation on sparse matrices can be downloaded from https://github.com/JRowleyLab/HiCSampler^81^, https://github.com/JRowleyLab/HiCNoiseMeasurer^82^, and https://github.com/aidenlab/EigenVector^83^. These are open source and include source code as well as implementations in python and C + +. Simulation software can be found at https://github.com/DiPierroLab/NuChroM^84^.
