# Supplementary material for: Chromatin alternates between A and B compartments at kilobase scale for subgenic organization
Source: Nat Commun. 2023 Jun 6;14:3303. doi: 10.1038/s41467-023-38429-1 (PMC10244318; doi:10.1038/s41467-023-38429-1)
Supplement: Supplementary file 1 — Supplementary Information [file 41467_2023_38429_MOESM1_ESM.docx]

**Chromatin alternates between A and B compartments at kilobase scale for subgenic organization**

Supplementary Information

Supplementary Discussion

Supplementary References

Supplementary Figures 1-13

Supplementary Tables 1-7

**Supplemental Discussion**

*Hi-C Sequencing Depth Guidelines*

Because this Hi-C sequencing effort represents 90x higher coverage than the average published map (Supplementary Figure 1c), we tested the effects of coverage on analysis with the aim of establishing sequencing depth guidelines for *in situ* Hi-C data. We randomly subsampled read-pairs to create Hi-C maps with various sequencing depths. While the slope of diagonal decay (i.e., the average signal at each distance) was unchanged (Supplementary Figure 1f), the information contained in bin pairs was dependent on sequencing depth (Supplementary Figure 1g-j). For example, when examining all bin-pairs within 1 Mb of each other, 3 billion intra-chromosomal read-pairs are required to achieve a frequency at which 90% of the bin-pairs have at least 1 read (Supplementary Figure 1g&h). We also found that Hi-C heatmaps from samples with low read coverage result in bigger signal differences between neighboring bin pairs, creating noisier maps (Supplementary Figure 1k-m). Notably, by these two metrics, published Hi-C maps with less than 3 billion intra-chromosomal read-pairs have low information content and high noise levels when placed in bins smaller than 5 kb.

We also tested how sequencing depth affects feature identification. Sequencing depth dramatically influences long-range compartment interactions (Supplementary Figure 2l-m), and maps with <7 billion intra-chromosomal read-pairs failed to identify compartments at 500 bp resolution (Supplementary Figure 2m).

We examined CTCF loops in subsampled maps and found that low sequencing depth resulted in lower signal-to-background ratios measurable by aggregate peak analysis (APA) (Supplementary Figure 10b-d). As such, sequencing depth correlates with the number of identifiable loops (Supplementary Figure S10e). For example, we estimate that *in situ* Hi-C maps with <=500 million intra-chromosomal read-pairs, i.e., 90% of published maps (Supplementary Table 2), may be missing more than 57% of loops simply due to low coverage (Supplementary Figure 10f). However, sequencing depth does not dramatically impact the potential false-positive rate (Supplementary Figure 10g). We investigated why some loops are missed with lower sequencing depths, finding that convergent motifs are prominent on loops called at all depths (Supplementary Figure 10h). Instead, sequencing depth correlates with the ability to call loops at longer distances (Supplementary Figure 10i). Most published Hi-C datasets have less than 500 million intra-chromosomal contacts, suggesting that these data were unable to detect the majority of CTCF loops larger than 1 Mb. Based on the plateau of the distance bias (Supplementary Figure 10i), we estimate that only 5% of CTCF loops are greater than 3.4 Mb in size (Supplementary Figure 10j).

Recent methodological advances, particularly Micro-C, have provided fine-fragmentation maps of chromatin organization. Therefore, we asked why subgenic discordant compartmentalization was not previously examined. We used POSSUMM in H1 Micro-C maps and achieved compartment calls at 5 kb resolution (Supplementary Figure 13a). Note that the eigenvector with smaller bins failed to denote compartments in these maps; thus, the Ultra-Res map reported in this study provides A/B compartments at an order of magnitude higher than possible from these previous Micro-C maps. We note that the 5 kb resolution was also possible due to POSSUMM and that these previous studies did analyze compartment calls beyond 100 kb resolution^1,2^. To further examine compartments in Hi-C vs. Micro-C, we compared the eigenvectors resulting from Hi-C and Micro-C in H1 cells, finding a correlation of .92 between them (Supplementary Figure 13b).

**Supplementary References**

1. Krietenstein, N. *et al.* Ultrastructural Details of Mammalian Chromosome Architecture. *Mol Cell* **78**, 554-565 e557 (2020). https://doi.org:10.1016/j.molcel.2020.03.003
2. Hsieh, T. S. *et al.* Resolving the 3D Landscape of Transcription-Linked Mammalian Chromatin Folding. *Mol Cell* **78**, 539-553 e538 (2020). https://doi.org:10.1016/j.molcel.2020.03.002

**Supplementary Figures 1-13 (Next Page)**


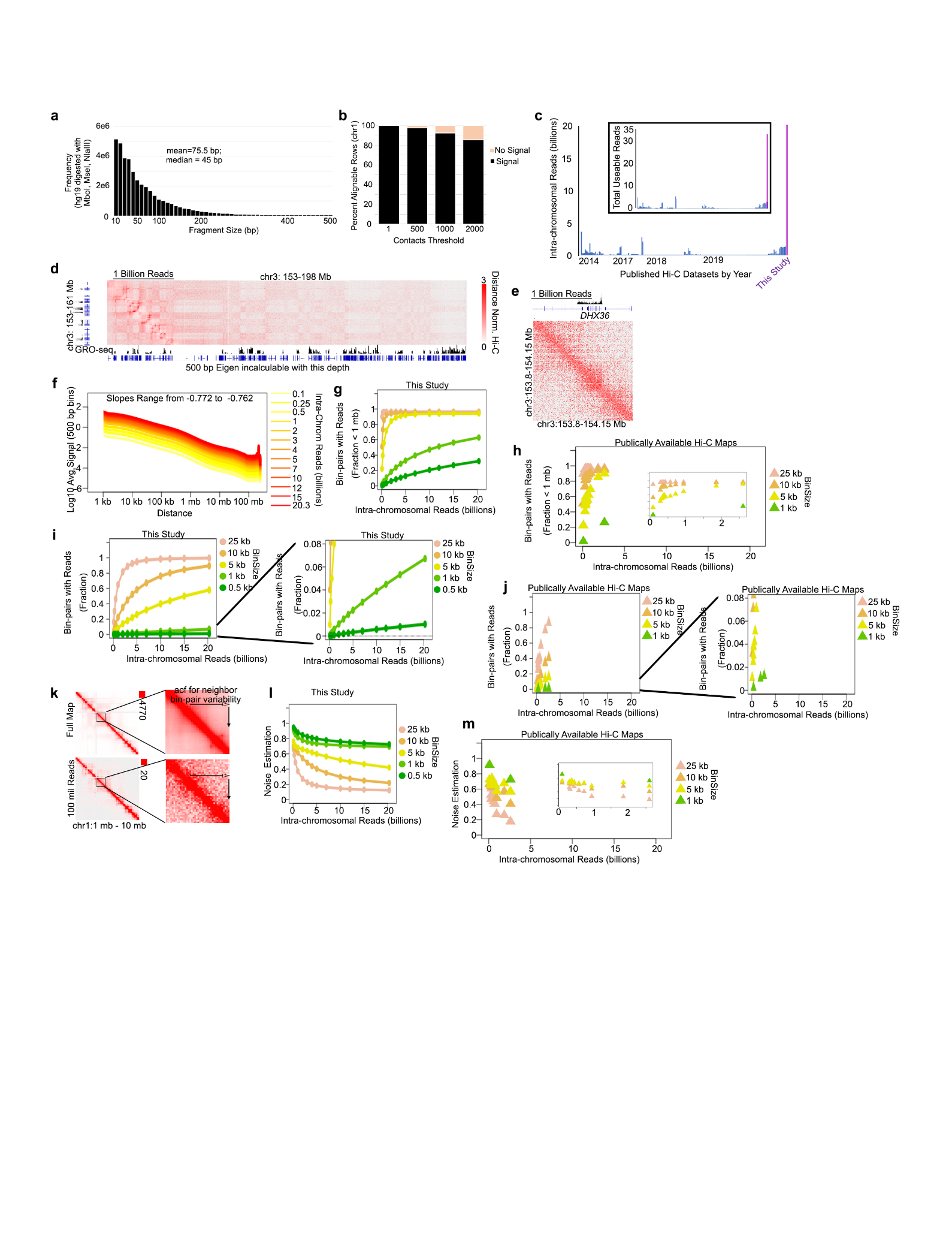


Supplementary Figure 1. **Hi-C map information content is reliant on sequencing depth. a** Frequency of finding a digested end after digestion with MboI, MseI, or NlaIII. **b** Percent of alignable rows with Hi-C signal at different thresholds. **C.** Number of intra-chromsosomal and total (inset) useable read-pairs in the full LCL Hi-C map (purple) compared to publicly available maps (blue). **d&e** Example of long-range compartment interactions (d) and a compartment domain (e) in a Hi-C map when with only 1 billion intra-chromosomal read-pairs. The black track displays transcription measured by GRO-seq. **f** Diagonal decay in our full 20.3 billion map and maps with subsampled read-pairs. **g&h** Fraction of bin-pairs at distances < 1 Mb that has at least 1 read in the full and subsampled maps (g) and published maps (h). **i&j** Fraction of bin-pairs at all distances that have at least 1 read considering all distances in the full 20.3 billion map and in maps with subsampled read-pairs (i) or in published maps (j). The right panels show the data when zoomed in on the y-axis. **k** Example of how sequencing coverage reduces noise in Hi-C maps and how the autocorrelation function (ACF), which measures similarities between neighboring bin-pairs, can be used to estimate noise. **l&m** Noise estimated from the inverse of the autocorrelation function for the full and subsampled maps (l) and in published Hi-C maps (m).


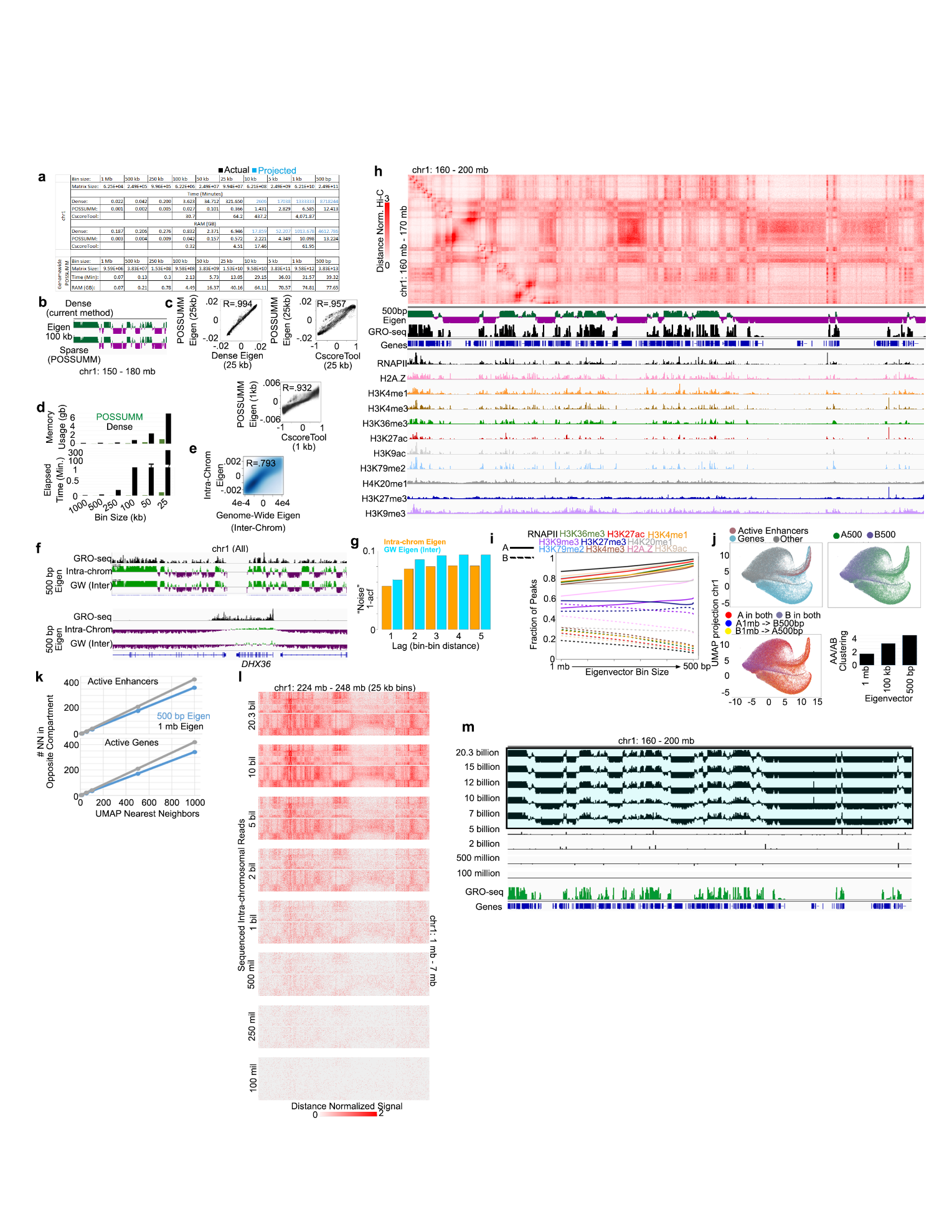
Supplementary Figure 2. **POSSUMM combined with deep sequencing provides A/B compartments at 500 bp resolution. a-f** Evaluation of POSSUMM including time and memory (a&d) [Note, that resolutions beyond 25 kb were not possible to calculate using the dense matrix method, thus projected values are calculated from a trend line fit to the available data points], example loci showing similarity to other methods (b), correlation to other methods in 25 kb bins R=0.994 (Pearson), including CScoreTool at 25 kb (R=0.957) and at 1 kb (bottom) R=0.932 (c), and correlation between processing the intrachromosomal vs. genome-wide maps (e) along with example loci (f). **g** Noise calculated by 1- the autocorrelation function (ACF) with varying amounts of lag. **h** Example of compartments seen by distance normalized Hi-C, the eigenvector at 500 bp resolution, and the corresponding transcription (GRO-seq), RNA polymerase (RNAPII), and various histone marks. **i** Fraction of ChIP-seq peaks assigned to the A (solid) or B (dashed) compartment when identified with various bin sizes. **j** UMAP clustering of bins based on histone marks, colored by Active enhancers (H3K27ac), orange and active genes (TPM >= 1), blue, other loci grey; by compartment status in the 500 bp map as A green B purple, or by differences between the 500bp and 1 Mb eigenvector. Bottom Right: Clustering distance of each A point to the nearest 10 A points vs. the nearest 10 B points for the 500 bp, 100 kb, and 1 Mb compartment eigenvectors. **k** In UMAP plots clustered by histone modification, the number of nearest neighbors which were in opposite compartments (Y-axis) when examining various numbers of nearest neighbors (X-axis). **l** Example of compartments seen by distance normalized Hi-C and the eigenvector at 500 bp resolution in maps with various sequencing depths. Active sites are represented by high GRO-seq signal. **m** Comparison of 500 bp compartment identification in maps with various sequencing depths.

**
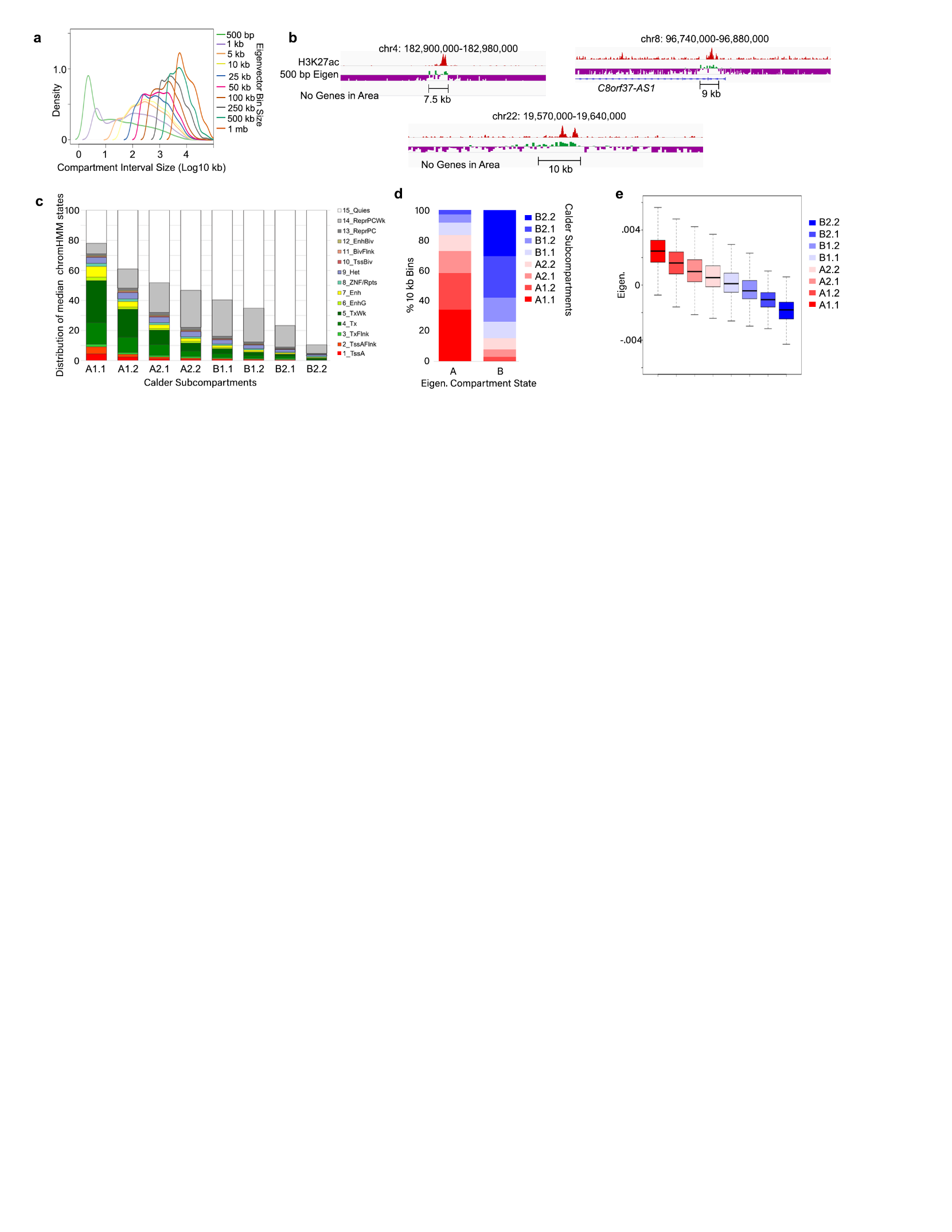
**Supplementary Figure 3. **Small compartment intervals reflect chromatin states.** **a** Histogram density plot of compartment sizes when identified at various resolutions. **b** Examples of A compartment intervals less than 10 kb in size. **c** Enrichment of chromHMM states inside subcompartments. **d** Distribution of subcompartments for eigenvector A and B compartments. **e** Eigenvector at each subcompartment classification. Boxplots represent the median and the interquartile range (IQR), with whiskers representing 1.5*IQR. n =bins in each subcompartment 1542236 B2.2, 755398 B2.1, 470441 B1.2, 378580 B1.1, 349045 A2.2, 398312 A2.1, 580964 A1.2, 1007893 A1.1.


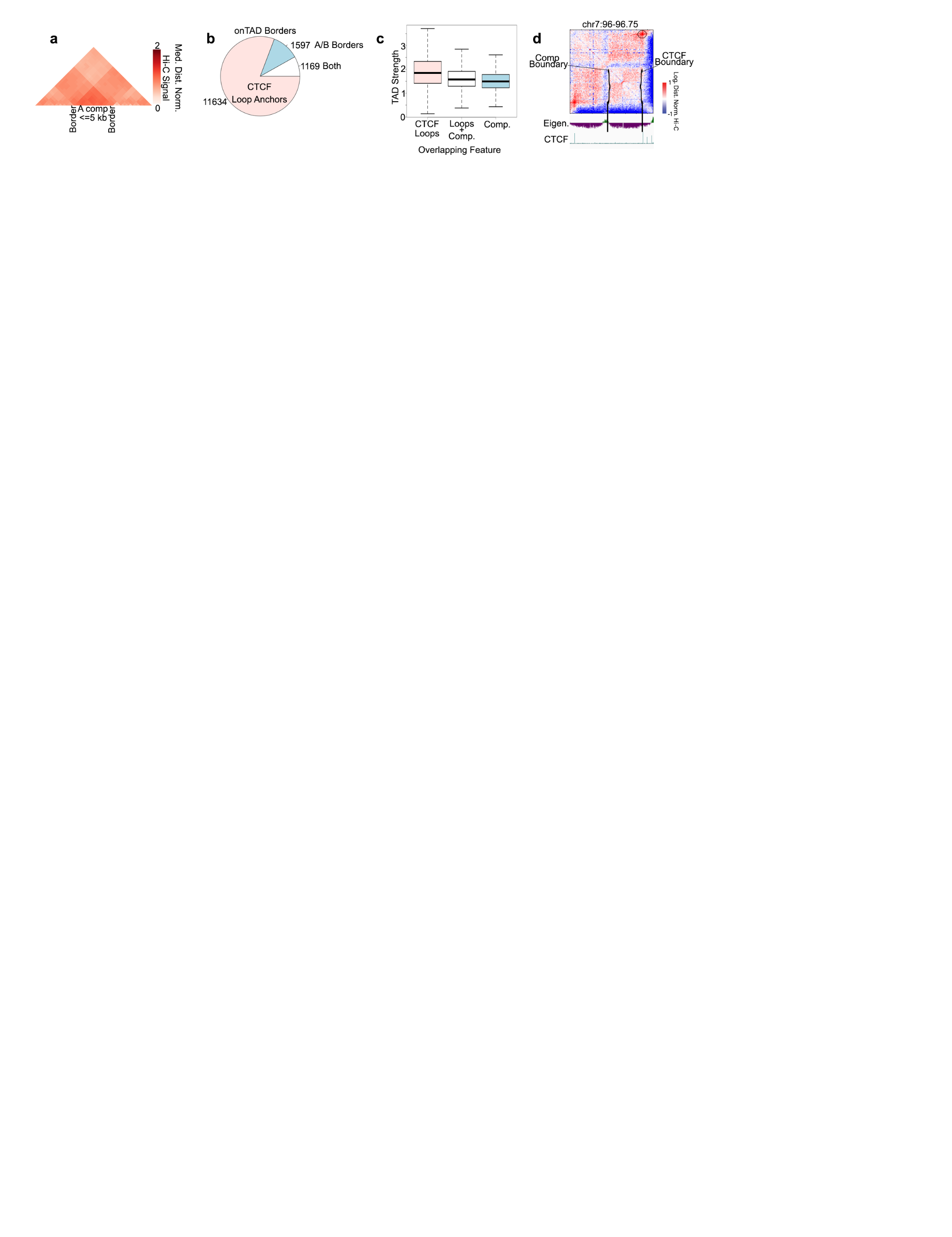


Supplementary Figure 4. **Small compartments create domains of interactions. a** Median distance normalized Hi-C signal near the diagonal for small, <= 5 kb A compartment domains identified by the POSSUMM eigenvector. **b** Number of onTAD borders corresponding to CTCF loop anchors vs. compartment domains. **c** Strength of TADs with borders overlapping CTCF loop anchors or compartment domains. Boxplots represent the median and the interquartile range (IQR), with whiskers representing 1.5*IQR. N= 28884 (CTCF loops), 3131 (Loops + Comp), 3322 (Comp). **d** Example of a domain where one border is compartmental while the other is a CTCF loop anchor. Distance-normalized Hi-C signal is shown.


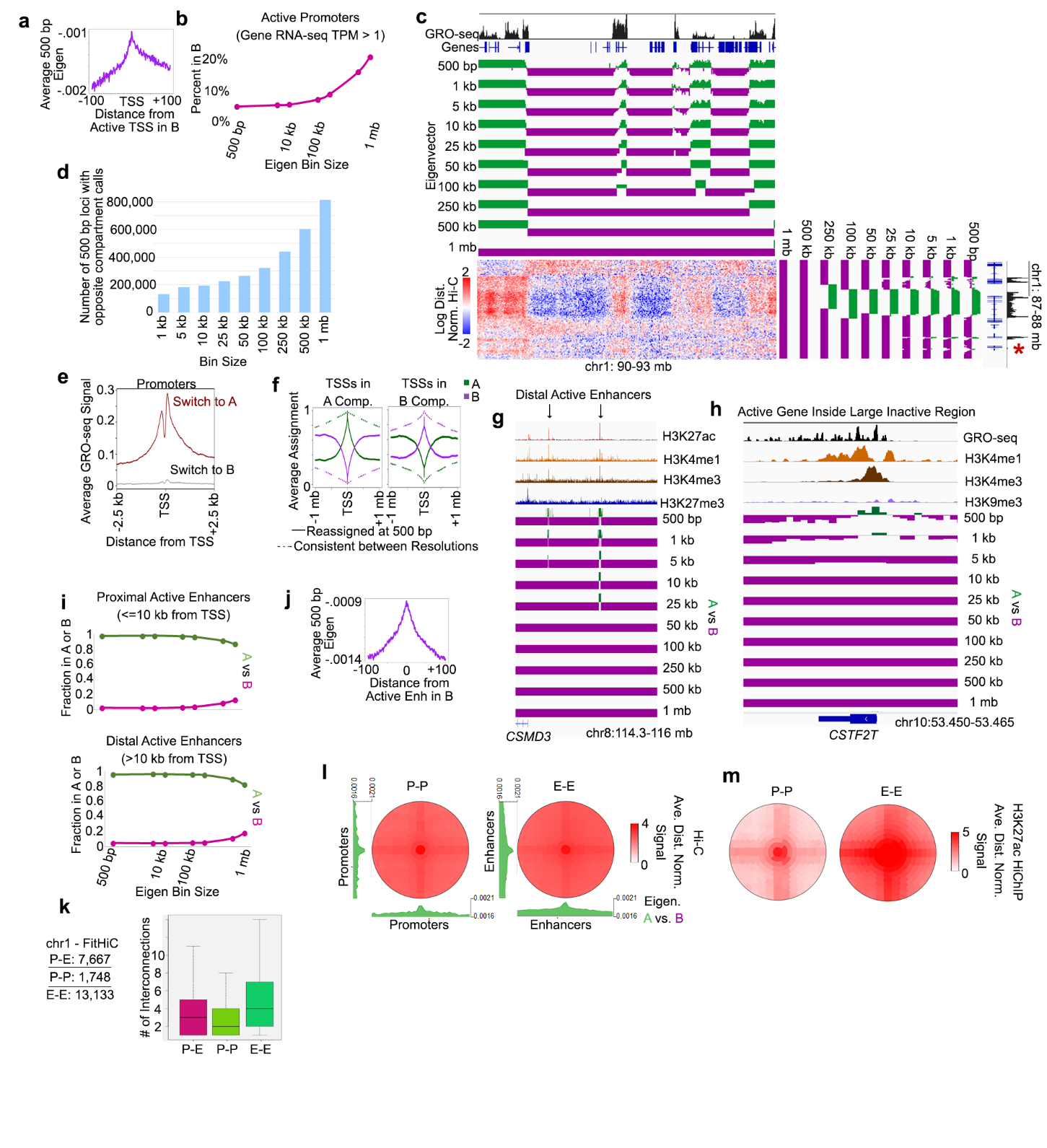


Supplementary Figure 5. **Active regulatory elements are overwhelmingly in the A compartment. a** Average POSSUMM eigenvector around TSSs assigned to the B compartment. **b** Percentage of active promoters assigned to B when compartments are identified at various resolutions. **c** Example of small compartment domains only identifiable at high-resolution (red asterisks). Log transformed and distance normalized Hi-C map is shown alongside the eigenvector tracks at various bin sizes. **d** Number of loci with opposite compartment calls when they are identified in coarser bins. **e** Average GRO-seq signal near TSSs that are reassigned to A (red) of B (grey) compartments. **f** The average A (green) and B (purple) compartmental status at 500 bp resolution for TSSs and the surrounding region for those TSSs reassigned compartments (solid line) vs. those consistent between resolutions (dashed). **g** Examples of distal active enhancers with mismatched compartment assignments at coarse resolutions. **h** Example of an active promoter with mismatched compartment assignment at coarse resolutions. **i** Percentage of active proximal (top) or distal (bottom) enhancers assigned to A (green) or B (purple) when compartments are identified at various resolutions. **j** Average POSSUMM eigenvector around active enhancers assigned to the B compartment. **k** Number of FitHiC Interactions and the number of connections each promoter or enhancer has with other elements. Boxplots represent the median and the interquartile range (IQR), with whiskers representing 1.5*IQR; n=7667 P-E, 1748 P-P, 13133 E-E. **l** Average eigenvector (green tracks) at anchors and average Hi-C signal at FitHiC Promoter-Promoter (P-P) and Enhancer-Enhancer (E-E) loops. **m** Average H3K27ac HiChIP signal at these loops.


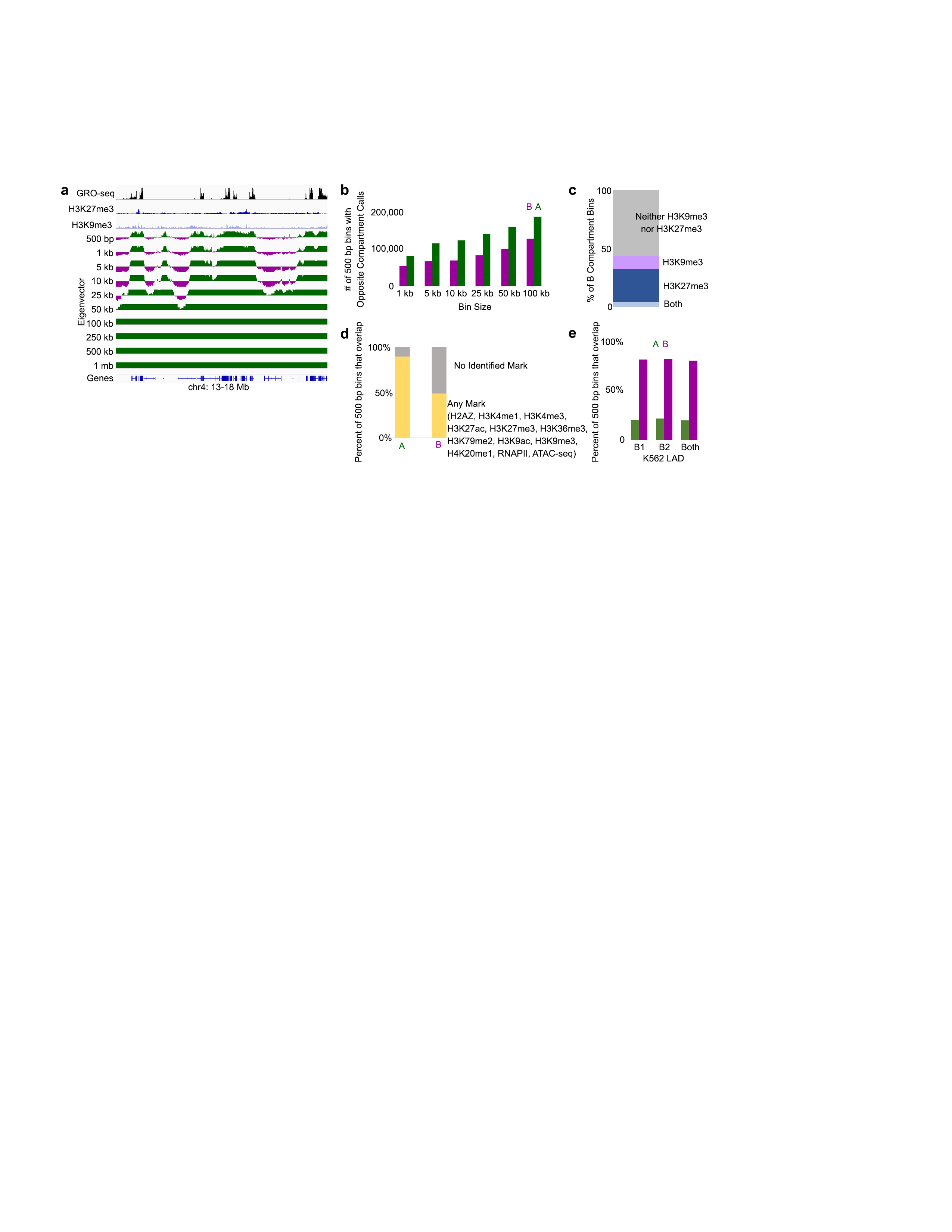
Supplementary Figure 6. **Characteristics of the B compartment. a** Example of a small B compartment interval only identifiable at high-resolution. **b** Number of 500 bp bins with opposite A or B compartment calls at coarser resolutions. **c** Percentage of B compartment bins that overlap H3K27me3, H3K9me3, or neither peaks. **d** Percentage of A and B compartment bins overlapping any listed marks. **e** Percentage of A and B compartment bins that overlap LADs defined in K562 cells.


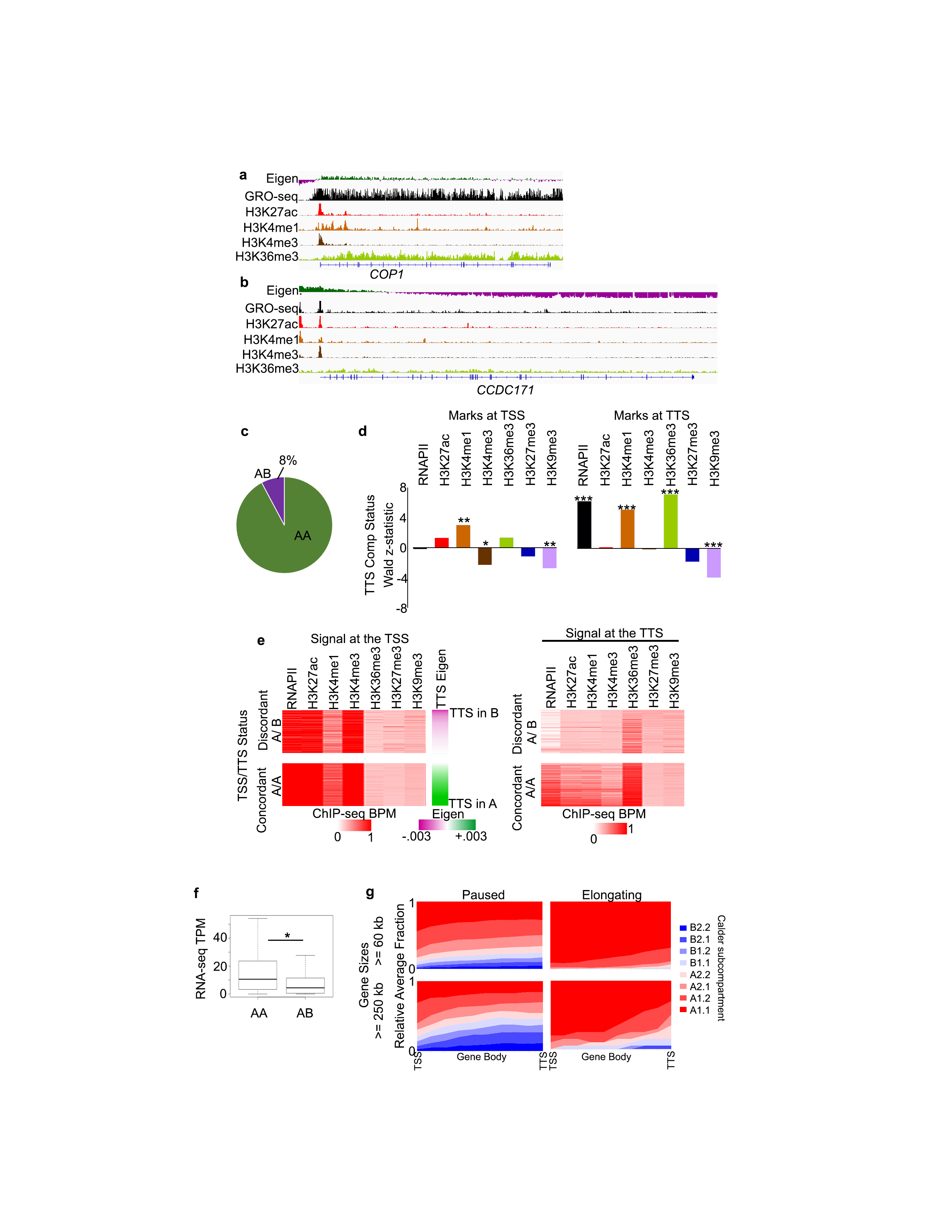
Supplementary Figure 7. **Chromatin marks at the TTS can explain compartmental segregation from the TSS. a&b** Examples of a gene with different GRO-seq distributions and A compartmental distributions along the gene body. **c** Percentage of genes that exhibit discordant compartment status irrespective of size. **d** Results of logistic regression tests for the relationship between marks at the TSS or TTS vs. the compartmental status of the TTS. * p<.05, ** p<.01, *** p<.001 from the two-tailed t-test within the logistic regression model. For TSS and TTS these p-vaules were RNAPII=0.90625 and 5.83e^-10^, H3K27ac=0.18643 and 0.89943, H3K4me1=.00254 and 3.35e^-7^, H3K4me3=0.0266 and 0.91468, H3K36me3=0.18109 and 1.03e^-12^, H3K27me3=0.27309 and 0.08, H3K9me3=0.00819 and 0.00012. **e** ChIP-seq signal at the TSS and TTS of discordant A/B genes vs. concordant A/A genes. Genes are sorted by the TTS compartmental signal. The intensity of red corresponds to the ChIP-seq signal, while green to pink represents negative to positive A/B compartment eigenvector. **f** Expression of genes with concordant (AA) vs. discordant (AB) compartment status measured by RNA-seq, TPM normalized – Transcripts Per Million. Boxplots represent the median and the interquartile range (IQR), with whiskers representing 1.5*IQR. * indicate p< 2.2e-16 two-sided Wilcoxon rank-sum test; n=6021 AA and 510 AB. **g** Average fraction of each gene body segment overlapping the different subcompartments. Two different size limits, >=60 kb (n=1,660 paused and n=415 elongating) and >= 250 kb (n=274 paused and n=20 elongating), are shown for comparison. Red to blue shadings correspond to A1.1 to B2.2 subcompartment designations, respectively.


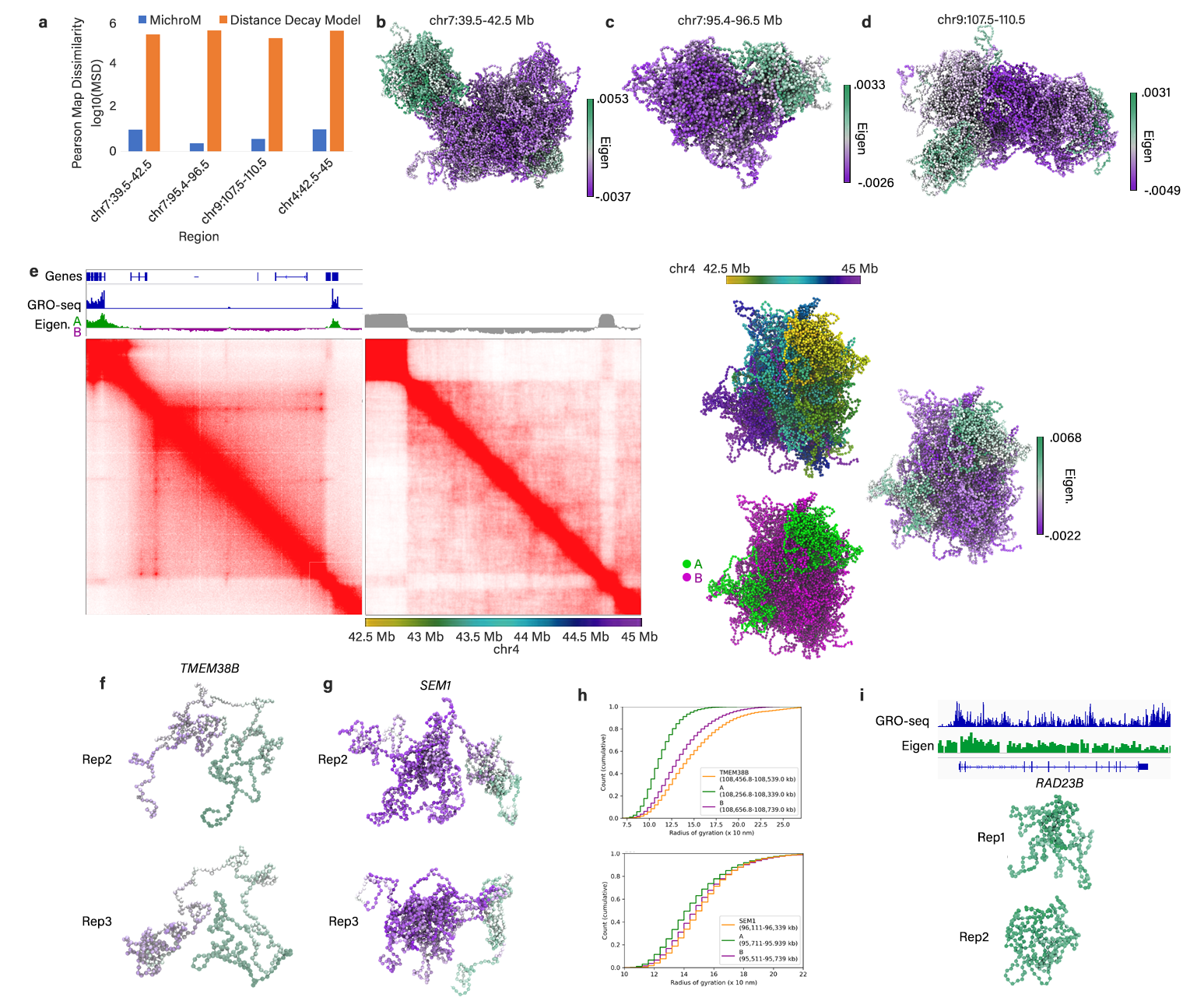
Supplementary Figure 8. **MiChroM models of genes with discordant compartments.** **a** Log10 Mean Squared Difference (MSD) for the MiChroM (blue) or simple distance decay (orange) model compared to the actual Hi-C map. **b-d** Representative modeled structures colored by the corresponding Hi-C eigenvector. **e** Example of simulated chromatin segment for chr4:42.5-45 Mb. Hi-C map (left) compared to simulated map (right) at 1 kb resolution after modeling. A representative structure of the region is shown, colored by chromosomal coordinates (top), A/B state (bottom), or Hi-C eigenvector (mid). **f&g** Two additional representative models of discordant compartments on *TMEM38B* and *SEM1* produced during simulation. **h** Radius of gyration calculations for *TMEM3B* and *SEM1* (yellow) compared to randomly chosen equal-sized regions completely in A (green) or B (purple). The two-sided Kolmogorov-Smirnov test of each compared to A and B gave p=0.0 for each. **i** GRO-seq, compartment eigenvector, and structural simulations of *RAD23B*, a gene entirely in the A compartment.


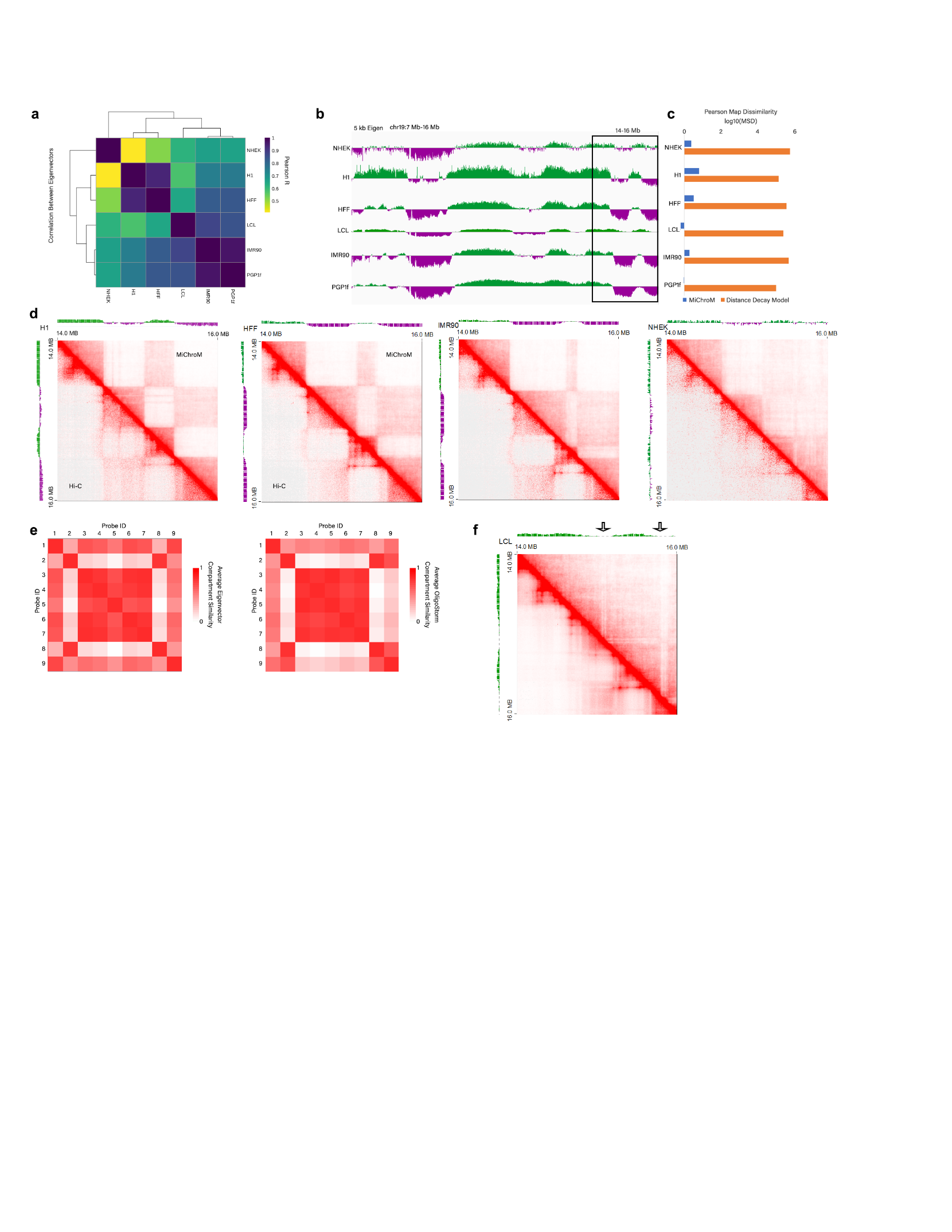


Supplementary Figure 9. **MiChroM models in various cell types. a-c** Correlation of the **a** eigenvector and **c** log10 MSD (Mean Square Difference) **c** for the region shown in different cell lines. To determine similarity of compartment signal, MSD is calculated on the Pearson correlation matrices. **d** Comparison of Hi-C and MiChroM maps in different cell lines. **e** Compartment similarity matrix for each imaged segment based on the imaging results (right) or the Hi-C eigenvector (left). **f** MiChroM for the OligoStorm imaged region in LCL, which has ambiguous compartment status, highlighting a limitation of MiChroM to simulate regions with an ambiguous eigenvector.


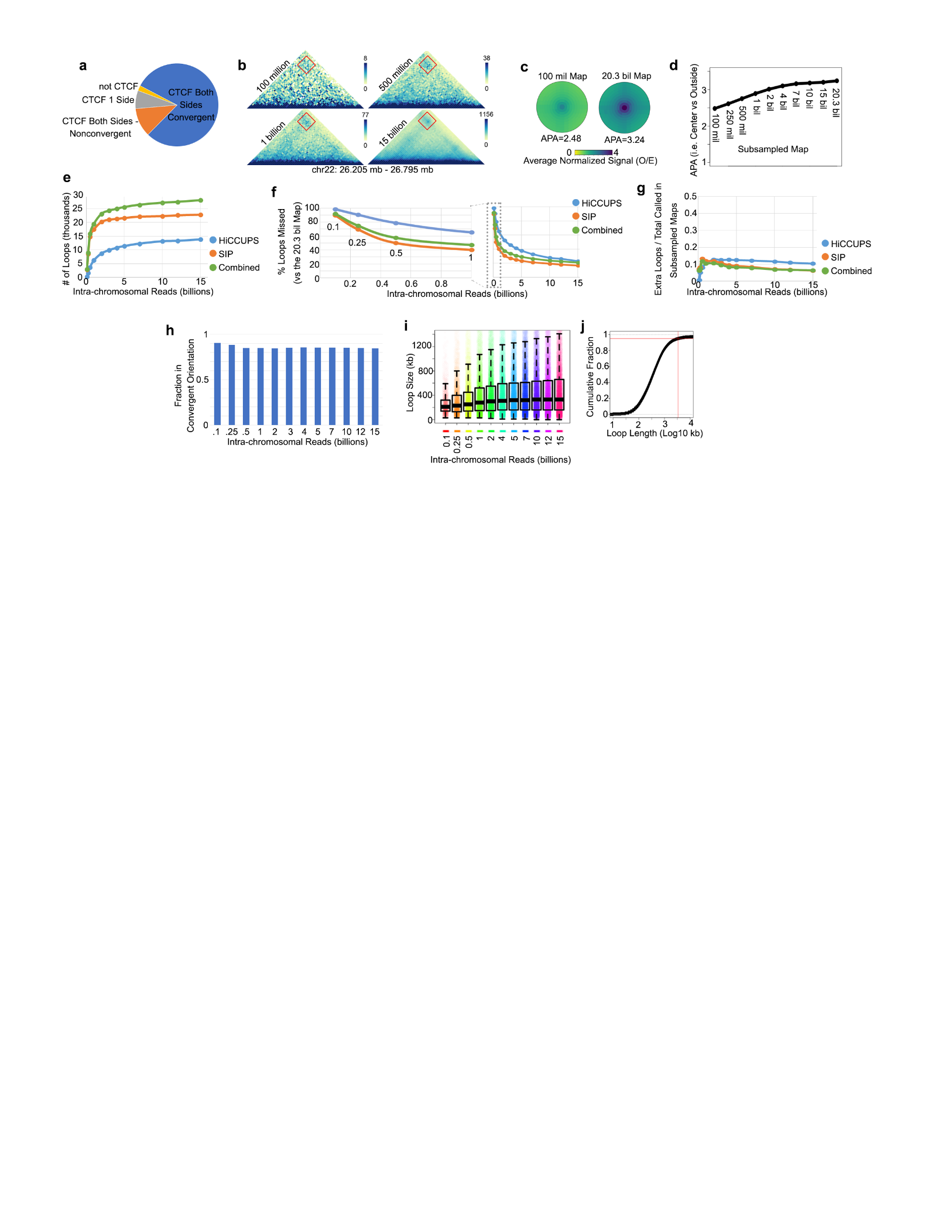
Supplementary Figure 10. **The impact of sequencing depth on CTCF loop analysis. a** The distribution of loops that had CTCF in convergent orientation on both anchors (blue), in other orientations (orange), on only one side (grey), or had no evidence of CTCF (yellow). **b** Example of a loop that is missed with lower sequencing depth. Numbers represent intra-chromosomal read pairs. **c** Average CTCF loops signal in a Hi-C contact map with 100 million reads vs. 20.3 billion reads. **d** Aggregate peaks analysis (APA) in subsampled maps and the full 20.3 billion map. Numbers represent intra-chromosomal read pairs. **e** Number of loops called in subsampled maps as called by HiCCUPS (blue), SIP (orange), or when combined (green). **f** Percentage of loops missed in subsampled maps. **g** The number of loops called only in the subsampled map vs. the full 20.3 billion map as a ratio to the total called for HiCCUPS (blue), SIP (orange), or when combined (green). **h** The fraction of loops called in subsampled maps that have CTCF in convergent orientation. **i** Sizes of loops identifiable in Hi-C maps with various sequencing depths. Boxplots represent the median and the interquartile range (IQR), with whiskers representing 1.5*IQR. Data density is shown behind and colored according to the sequencing depth. From left to right, n= 2,838; 8513; 14802; 17441; 20203; 21254; 21607; 22081; 22281; 22572; 22795. **j** Cumulative fraction of loop sizes in the full map.


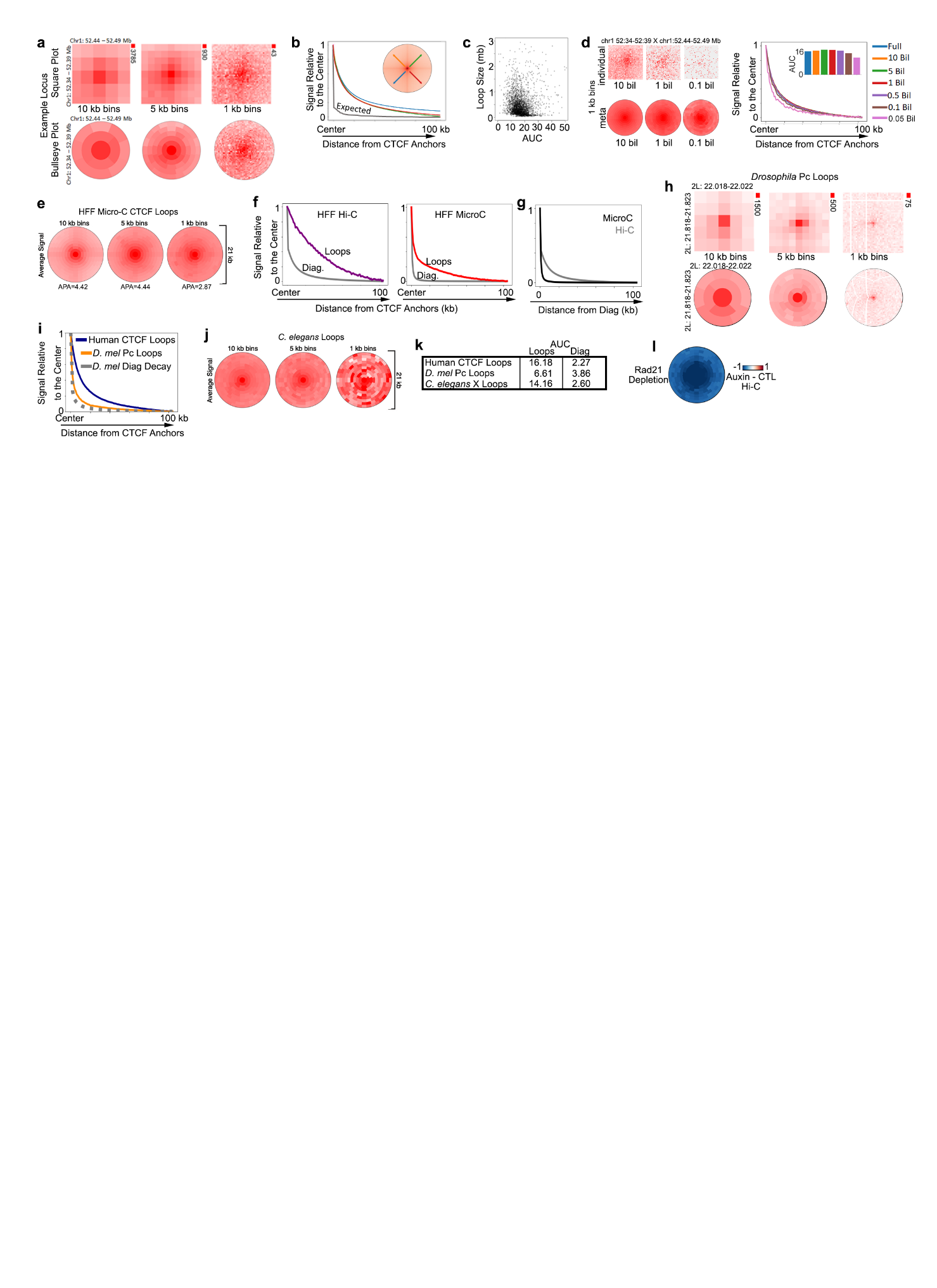
Supplementary Figure 11. **Characteristics of diffuse vs. punctate loops. a** Square (top) and bullseye (bottom) views of an example CTCF loop in human cells when binned at 10, 5, or 1 kb. **b** Average loss of signal starting at loops and moving inside (blue), parallel (red and orange), or outward (green). Average signal loss at the diagonal is shown for reference (grey). **c** The area under the curve (AUC) vs. the size of each CTCF loop. **d** Example of how sequencing depth impacts visual identification of diffuse CTCF loop interactions at individual loci (top) vs. metaplot analysis (bottom), and the impact on AUC measurements (right). **e** Average HFF Micro-C signal at CTCF loops when binned at 10, 5, or 1 kb. **f** Average loop-proximal interactions (purple) in HFF Hi-C maps and Micro-C maps vs. the diagonals of each (gray). **g** Average diagonal decay of Micro-C (black) and Hi-C (gray). **h** Square (top) and bullseye (bottom) views of an example Pc loop in *D. melanogaster* cells when binned at 10, 5, or 1 kb. **i** Average Hi-C signal in 1 kb bins at each radial distance away from human CTCF loop anchors (blue) vs. *D. melanogaster* Pc loops (orange). Average signal at the *D. melanogaster* Hi-C diagonal is shown for reference (grey). **j** Metaplots of *C. elegans* loops at 10, 5, and 1 kb resolutions. **k** Table of AUC values for loops and the diagonal in human, *D. melanogaster*, and *C. elegans* Hi-C maps. **l** Average plots after RAD21 degradation showing the change in Hi-C signal at the loop and in proximal regions.


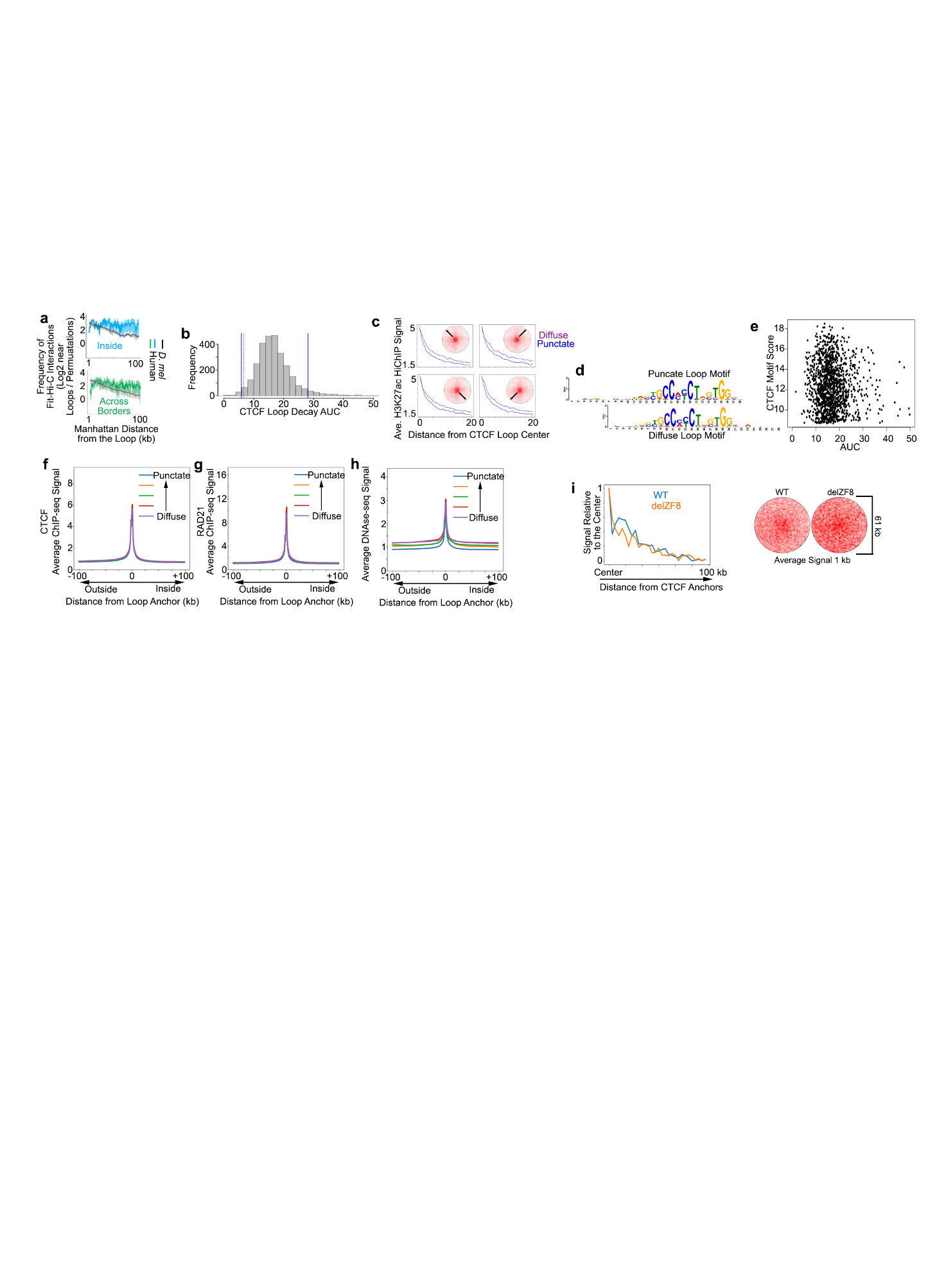
Supplementary Figure 12. **Chromatin accessibility correlates with diffuse loops.** **a** Frequency of enhancer-promoter interactions determined by Fit-Hi-C in the area proximal to CTCF loops in human cells vs. *Drosophila* Pc loops. Interactions that are completely interior to loops vs. those that cross over one or both anchors are shown. The shaded area represents the standard deviation across 10 permutations. b Histogram of diffuse signal AUC (area under the curve) values for CTCF loops. Black vertical lines indicate a standard deviation of 1.5. The red vertical line indicates the average AUC of *D. melanogaster* Pc loops. **c** H3K27ac HiChIP signal proximal to diffuse (purple) vs. punctate (blue) CTCF loops. Arrows indicate the direction of measurement. **d** CTCF motifs found at diffuse vs. punctate loops. **e** Comparison of CTCF motif score at anchors to the AUC. **f-h** Average profile of CTCF ChIP-seq signal, RAD21 ChIP-seq signal, and DNAse-seq signal at the anchors of loops that show different diffusiveness. **i** Metaplots and proximal signal comparison of wild-type to ZF8 mutant.


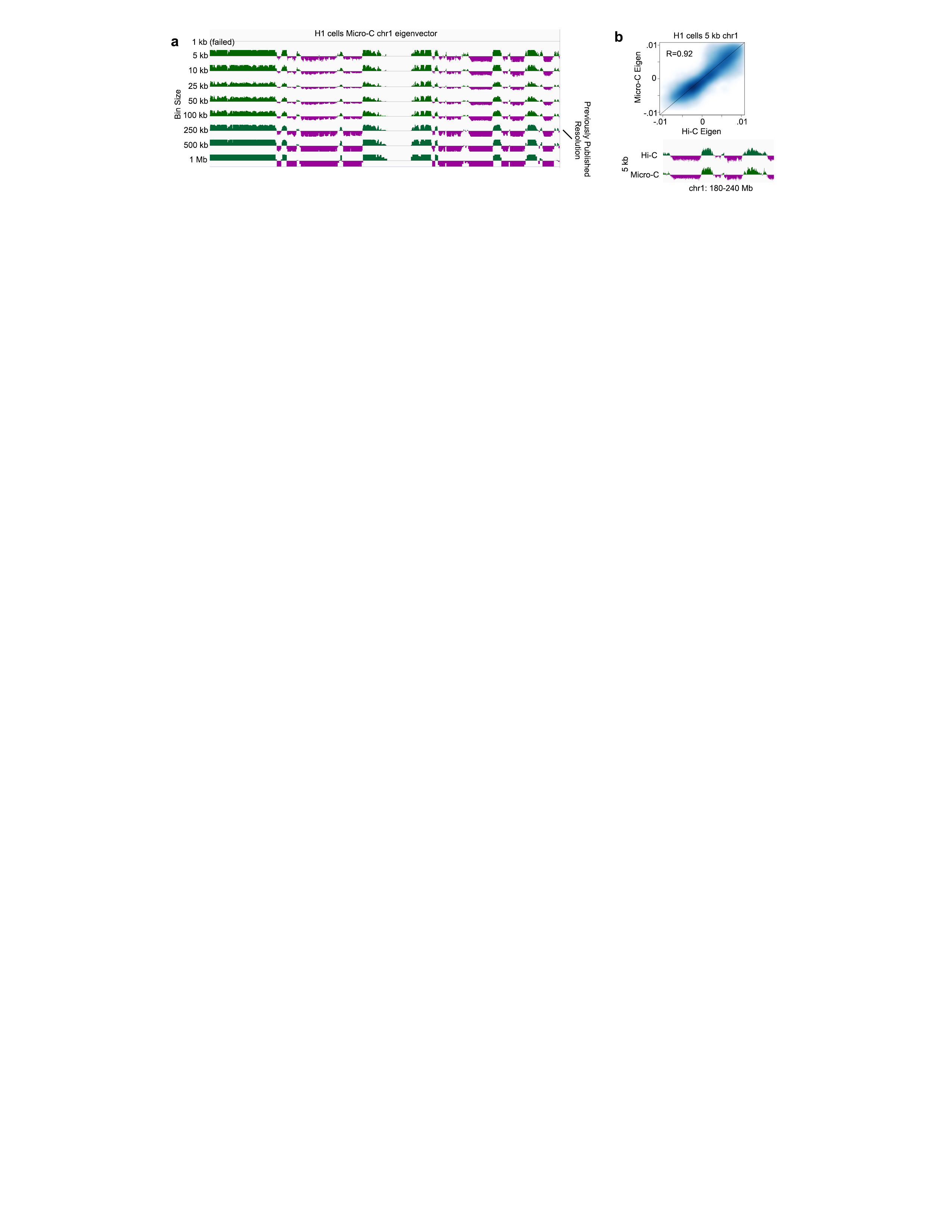
Supplementary Figure 13. **Compartments measured by Micro-C. a** POSSUMM compartment eigenvector at various resolutions in H1 Micro-C data. **b** Comparison of POSSUMM calls in Micro-C vs. Hi-C in H1 cells at 5 kb. R=0.92 (Pearson). The bottom tracks show an example locus.

**Supplementary Tables**

| Sequenced Read Pairs: 42,467,875,383 |
| --- |
| Normal Paired: 23,937,977,379 (56.37%) |
| Chimeric Paired: 14,294,168,018 (33.66%) |
| Chimeric Ambiguous: 3,382,008,102 (7.96%) |
| Unmapped: 853,721,882 (2.01%) |
| Alignable (Normal+Chimeric Paired): 38,232,145,397 (90.03%) |
| Unique Reads: 34,806,960,717 |
| PCR Duplicates: 3,385,126,713 |
| Optical Duplicates: 203,288,499 |
| Intra-fragment Reads: 840,557,070 (1.98% / 2.41%) |
| Below MAPQ Threshold: 3,217,457,396 (7.58% / 9.23%) |
| Hi-C Contacts: 30,815,303,487 (72.56% / 88.36%) |
| Ligation Motif Present: 0 (0.00% / 0.00%) |
| 3' Bias (Long Range): 0% - 0% |
| Pair Type % (L-I-O-R): 25% - 25% - 25% - 25% |
| Inter-chromosomal: 7,663,533,305 (18.05% / 21.98%) |
| Intra-chromosomal: 23,151,770,182 (54.52% / 66.39%) |
| Short Range (<20Kb): 7,905,477,803 (18.62% / 22.67%) |
| Long Range (>20Kb): 15,246,211,030 (35.90% / 43.72%) |

Supplementary Table 1. **Mapping and filtering statistics for the Hi-C in this study.** Number of reads before and after each processing step.

| Accession | File Type | | Total reads | Cis reads | Short cis reads (<20kb) | Trans reads | Date Published |
| --- | --- | --- | --- | --- | --- | --- | --- |
| 4DNES7L8Z2KV | contact | list-combined | 6.94E+08 | 4.1E+08 | 1.28E+08 | 1.55E+08 | 2/21/2017 |
| 4DNES49TDMJM | contact | list-combined | 7.18E+08 | 4.25E+08 | 1.76E+08 | 1.16E+08 | 2/21/2017 |
| 4DNESWEF2AHT | contact | list-combined | 5.79E+08 | 3.87E+08 | 1.01E+08 | 91143388 | 2/21/2017 |
| 4DNESHGZUBL9 | contact | list-combined | 5.64E+08 | 3.35E+08 | 1.2E+08 | 1.09E+08 | 2/21/2017 |
| 4DNES5R3O24W | contact | list-combined | 5.76E+08 | 3.23E+08 | 1.68E+08 | 85259649 | 2/21/2017 |
| 4DNESU4BQU4G | contact | list-combined | 8.12E+08 | 5.32E+08 | 1.55E+08 | 1.26E+08 | 2/21/2017 |
| 4DNES1QUXG92 | contact | list-combined | 4.53E+08 | 1.26E+08 | 2.64E+08 | 62876368 | 3/20/2017 |
| 4DNESWDLDMGN | contact | list-combined | 5.14E+08 | 2.68E+08 | 1.7E+08 | 76066471 | 3/20/2017 |
| 4DNESQMM4EBN | contact | list-combined | 4.11E+08 | 1.38E+08 | 2.33E+08 | 40843919 | 3/20/2017 |
| 4DNESKKSKG7Y | contact | list-combined | 1.07E+09 | 6.06E+08 | 3.29E+08 | 1.38E+08 | 3/20/2017 |
| 4DNES76KXUJ3 | contact | list-combined | 7.08E+08 | 4.84E+08 | 1.36E+08 | 88085859 | 5/4/2017 |
| 4DNESNLXBFMY | contact | list-combined | 4.16E+08 | 2.46E+08 | 92808070 | 77261380 | 5/4/2017 |
| 4DNESIU6F8HF | contact | list-combined | 4.23E+08 | 2.71E+08 | 91207112 | 61303212 | 5/4/2017 |
| 4DNESSQU7B76 | contact | list-combined | 4.14E+08 | 2.57E+08 | 94621908 | 62062447 | 5/4/2017 |
| 4DNES7RYT7KA | contact | list-combined | 62579604 | 35344938 | 16624521 | 10610145 | 7/12/2017 |
| 4DNES6U3U9PJ | contact | list-combined | 1.49E+08 | 83973483 | 37095565 | 28112410 | 7/12/2017 |
| 4DNESTHQ7CR1 | contact | list-combined | 65279070 | 35325834 | 18033073 | 11920163 | 7/12/2017 |
| 4DNESO6J5SH9 | contact | list-combined | 1.16E+08 | 55766173 | 32981980 | 26879414 | 7/12/2017 |
| 4DNESUQT299T | contact | list-combined | 61980196 | 34443888 | 16757618 | 10778690 | 7/12/2017 |
| 4DNESMXBLGKA | contact | list-combined | 1.45E+08 | 91998198 | 31232024 | 21990245 | 7/12/2017 |
| 4DNESSL62JQK | contact | list-combined | 2.03E+08 | 1.14E+08 | 47805198 | 40881991 | 7/12/2017 |
| 4DNESWPY49OL | contact | list-combined | 2.48E+08 | 1.41E+08 | 74199682 | 32828558 | 7/12/2017 |
| 4DNESXZHIUAU | contact | list-combined | 2.75E+08 | 1.29E+08 | 77376888 | 68396455 | 7/12/2017 |
| 4DNESICXDTH7 | contact | list-combined | 1.89E+08 | 91228461 | 56852135 | 40428780 | 7/12/2017 |
| 4DNES1J8MC4Q | contact | list-combined | 1.99E+08 | 94010460 | 61633474 | 42964423 | 7/12/2017 |
| 4DNES3JWOSVS | contact | list-combined | 1.05E+08 | 51189516 | 32975902 | 20789345 | 7/12/2017 |
| 4DNESOBRQ1WW | contact | list-combined | 66631303 | 47584250 | 16984296 | 2062757 | 7/12/2017 |
| 4DNESTRKO6LB | contact | list-combined | 86910877 | 21628543 | 33000862 | 32281472 | 7/12/2017 |
| 4DNES4KDIQNN | contact | list-combined | 5.73E+08 | 2.59E+08 | 1.55E+08 | 1.59E+08 | 10/5/2017 |
| 4DNES7RDRS69 | contact | list-combined | 6.16E+08 | 2.84E+08 | 1.58E+08 | 1.75E+08 | 10/5/2017 |
| 4DNESCOJ3ADI | contact | list-combined | 6.27E+08 | 3.04E+08 | 1.53E+08 | 1.71E+08 | 10/5/2017 |
| 4DNESJV9TH8Q | contact | list-combined | 2.55E+09 | 1.24E+09 | 4.39E+08 | 8.67E+08 | 10/5/2017 |
| 4DNES3QAGOZZ | contact | list-combined | 2.69E+09 | 1.38E+09 | 5.36E+08 | 7.73E+08 | 10/5/2017 |
| 4DNESD117NVX | contact | list-combined | 31998016 | 16377342 | 6762826 | 8857848 | 10/5/2017 |
| 4DNESLLMJ3JO | contact | list-combined | 27618880 | 13471392 | 6496379 | 7651109 | 10/5/2017 |
| 4DNES64ITOWM | contact | list-combined | 24853147 | 13011614 | 5580821 | 6260712 | 10/5/2017 |
| 4DNESNGDNQSG | contact | list-combined | 18841677 | 8862368 | 3918843 | 6060466 | 10/5/2017 |
| 4DNESD6N2Y6L | contact | list-combined | 9105818 | 4081727 | 2238539 | 2785552 | 10/5/2017 |
| 4DNESFPM1OFO | contact | list-combined | 8590091 | 4243171 | 1255600 | 3091320 | 10/5/2017 |
| 4DNES7N8J8KG | contact | list-combined | 10596090 | 5295320 | 1866483 | 3434287 | 10/5/2017 |
| 4DNESOGIV7NG | contact | list-combined | 6.45E+08 | 3.31E+08 | 1.77E+08 | 1.38E+08 | 10/5/2017 |
| 4DNESOQTEWX8 | contact | list-combined | 1.61E+08 | 80055591 | 37598299 | 43024339 | 10/5/2017 |
| 4DNESMLKX1ZM | contact | list-combined | 1.74E+08 | 75981091 | 45995947 | 52321369 | 10/5/2017 |
| 4DNES1NX2TKW | contact | list-combined | 3.68E+08 | 1.51E+08 | 1.75E+08 | 41456705 | 10/19/2017 |
| 4DNESQT4SR5G | contact | list-combined | 3.9E+08 | 1.55E+08 | 1.92E+08 | 43528194 | 10/19/2017 |
| 4DNES3Y26CEU | contact | list-combined | 3.77E+08 | 1.94E+08 | 1.34E+08 | 49026144 | 10/19/2017 |
| 4DNESUUFHGKJ | contact | list-combined | 3.95E+08 | 1.53E+08 | 1.99E+08 | 43420099 | 10/19/2017 |
| 4DNESGC3Z7E3 | contact | list-combined | 3.61E+08 | 1.5E+08 | 1.73E+08 | 38318988 | 10/19/2017 |
| 4DNES68MSHVU | contact | list-combined | 3.99E+08 | 1.57E+08 | 1.97E+08 | 44501456 | 10/19/2017 |
| 4DNESXS1M9JR | contact | list-combined | 3.68E+08 | 1.67E+08 | 1.56E+08 | 44970447 | 10/19/2017 |
| 4DNESBBYGJFA | contact | list-combined | 3.96E+08 | 1.6E+08 | 1.91E+08 | 45645804 | 10/19/2017 |
| 4DNESU4Y9CBF | contact | list-combined | 55197390 | 34219851 | 12113983 | 8863556 | 10/19/2017 |
| 4DNESXI5NKKT | contact | list-combined | 63878936 | 40020438 | 13333271 | 10525227 | 10/19/2017 |
| 4DNESUCLJAZ8 | contact | list-combined | 1.24E+08 | 81519000 | 26848021 | 15735191 | 10/19/2017 |
| 4DNESJ9SIAV5 | contact | list-combined | 5.37E+09 | 2.82E+09 | 1.05E+09 | 1.51E+09 | 10/19/2017 |
| 4DNESDXUWBD9 | contact | list-combined | 4.26E+09 | 2.11E+09 | 1.52E+09 | 6.27E+08 | 10/19/2017 |
| 4DNESI9RVI9Y | contact | list-combined | 10359452 | 4864232 | 1891456 | 3603764 | 4/26/2018 |
| 4DNESJ5Y5ZN8 | contact | list-combined | 12491179 | 7407430 | 2748362 | 2335387 | 4/26/2018 |
| 4DNESKZ1WYCN | contact | list-combined | 11381792 | 6460445 | 2748858 | 2172489 | 4/26/2018 |
| 4DNESKY5IGMT | contact | list-combined | 7369807 | 4233324 | 1505950 | 1630533 | 4/26/2018 |
| 4DNESWSTOQ92 | contact | list-combined | 8199465 | 3346903 | 1661083 | 3191479 | 4/26/2018 |
| 4DNES51DMKBB | contact | list-combined | 9107600 | 4663121 | 2021000 | 2423479 | 4/26/2018 |
| 4DNESHDMG1LU | contact | list-combined | 10249066 | 4937027 | 2759414 | 2552625 | 4/26/2018 |
| 4DNESTHU1G8W | contact | list-combined | 11133033 | 5445668 | 2125039 | 3562326 | 4/26/2018 |
| 4DNESG1W727L | contact | list-combined | 6336554 | 3121657 | 1145143 | 2069754 | 4/26/2018 |
| 4DNESHN4FT5O | contact | list-combined | 6905684 | 3268653 | 2010613 | 1626418 | 4/26/2018 |
| 4DNESD21O5UU | contact | list-combined | 10247395 | 5255517 | 2109025 | 2882853 | 4/26/2018 |
| 4DNES1XC6D9N | contact | list-combined | 13914617 | 7384859 | 3194524 | 3335234 | 4/26/2018 |
| 4DNESZ1UN35D | contact | list-combined | 10212946 | 5084766 | 2794188 | 2333992 | 4/26/2018 |
| 4DNESS3E2ITC | contact | list-combined | 9304013 | 5004953 | 1809702 | 2489358 | 4/26/2018 |
| 4DNESCQZYHLJ | contact | list-combined | 35216706 | 19249398 | 7887417 | 8079891 | 4/26/2018 |
| 4DNESEZ64E3W | contact | list-combined | 6414221 | 2983799 | 1834735 | 1595687 | 4/26/2018 |
| 4DNESM6U2PMJ | contact | list-combined | 7997210 | 4230465 | 1396519 | 2370226 | 4/26/2018 |
| 4DNESFSFD7PV | contact | list-combined | 11118934 | 5731655 | 2606103 | 2781176 | 4/26/2018 |
| 4DNESH3WR89R | contact | list-combined | 9202375 | 4821359 | 2055255 | 2325761 | 4/26/2018 |
| 4DNESIXD38O6 | contact | list-combined | 6693212 | 3463826 | 1478523 | 1750863 | 4/26/2018 |
| 4DNESUZCBCG9 | contact | list-combined | 32132036 | 17330693 | 6956046 | 7845297 | 4/26/2018 |
| 4DNESFJ1LEZ2 | contact | list-combined | 15373316 | 8454835 | 3831220 | 3087261 | 4/26/2018 |
| 4DNES5S3N3UN | contact | list-combined | 9448660 | 4855834 | 1554712 | 3038114 | 4/26/2018 |
| 4DNES2Z1IO3Y | contact | list-combined | 12368134 | 7038222 | 2289322 | 3040590 | 4/26/2018 |
| 4DNESA51WBOJ | contact | list-combined | 17400405 | 8068400 | 2266837 | 7065168 | 4/26/2018 |
| 4DNESV19UH2A | contact | list-combined | 20462007 | 10187566 | 3828421 | 6446020 | 4/26/2018 |
| 4DNESXWCEU6I | contact | list-combined | 6848462 | 3241053 | 1829758 | 1777651 | 4/26/2018 |
| 4DNESP7EFMLT | contact | list-combined | 12085658 | 6293931 | 2637159 | 3154568 | 4/26/2018 |
| 4DNESQNC15UK | contact | list-combined | 9489458 | 5026168 | 1834053 | 2629237 | 4/26/2018 |
| 4DNESZRLWUML | contact | list-combined | 10863843 | 5515148 | 2288664 | 3060031 | 4/26/2018 |
| 4DNES84ZICUS | contact | list-combined | 7005771 | 3565314 | 1589797 | 1850660 | 4/26/2018 |
| 4DNESK7WOJRD | contact | list-combined | 29824479 | 15574661 | 6293842 | 7955976 | 4/26/2018 |
| 4DNESLFAFVGO | contact | list-combined | 7183999 | 3479407 | 1848958 | 1855634 | 4/26/2018 |
| 4DNESOSF88NZ | contact | list-combined | 5757416 | 2919548 | 1553750 | 1284118 | 4/26/2018 |
| 4DNESXVOAC79 | contact | list-combined | 10959850 | 5613961 | 2254765 | 3091124 | 4/26/2018 |
| 4DNESD7DATO7 | contact | list-combined | 9095627 | 4784495 | 1656056 | 2655076 | 4/26/2018 |
| 4DNES2N7SYC4 | contact | list-combined | 13808614 | 7252491 | 3710579 | 2845544 | 4/26/2018 |
| 4DNES8YBULP1 | contact | list-combined | 7980137 | 4282007 | 1596003 | 2102127 | 4/26/2018 |
| 4DNESFNBTRO3 | contact | list-combined | 31649075 | 17502838 | 6781025 | 7365212 | 4/26/2018 |
| 4DNESDC4XDHT | contact | list-combined | 9708096 | 4789075 | 2205270 | 2713751 | 4/26/2018 |
| 4DNESERXQSXY | contact | list-combined | 3190568 | 1519402 | 724800 | 946366 | 4/26/2018 |
| 4DNESHF65RC4 | contact | list-combined | 31899568 | 16163097 | 6973336 | 8763135 | 4/26/2018 |
| 4DNESKLFZ31S | contact | list-combined | 36782146 | 19164835 | 8039651 | 9577660 | 4/26/2018 |
| 4DNES4GDH4BG | contact | list-combined | 1.16E+09 | 5.26E+08 | 3.58E+08 | 2.79E+08 | 4/26/2018 |
| 4DNES7C6LBWI | contact | list-combined | 9.15E+08 | 5.06E+08 | 2.19E+08 | 1.91E+08 | 4/26/2018 |
| 4DNES3O1B45O | contact | list-combined | 3.4E+08 | 1.73E+08 | 84295274 | 82883909 | 4/26/2018 |
| 4DNESE1VMAMD | contact | list-combined | 3.84E+08 | 2.25E+08 | 1.01E+08 | 57745415 | 4/26/2018 |
| 4DNES25ABNZ1 | contact | list-combined | 1.87E+09 | 1.1E+09 | 2.82E+08 | 4.92E+08 | 4/26/2018 |
| 4DNES7ODZ4MZ | contact | list-combined | 1.2E+09 | 6.86E+08 | 2.13E+08 | 3.05E+08 | 4/26/2018 |
| 4DNESYX7AQRY | contact | list-combined | 1.13E+09 | 6.16E+08 | 2.46E+08 | 2.63E+08 | 4/26/2018 |
| 4DNESVZ6QH33 | contact | list-combined | 2.39E+08 | 1.43E+08 | 55283623 | 40617843 | 7/16/2019 |
| 4DNESR93N4E3 | contact | list-combined | 2.09E+08 | 1.28E+08 | 40115834 | 41084468 | 7/16/2019 |
| 4DNES9J6QJQS | contact | list-combined | 3.05E+08 | 1.64E+08 | 60119989 | 81107895 | 7/16/2019 |
| 4DNESQICH2XW | contact | list-combined | 5.18E+08 | 98403965 | 89983326 | 3.29E+08 | 7/16/2019 |
| 4DNES1E3ET5M | contact | list-combined | 1.2E+08 | 54388106 | 24650549 | 40782522 | 7/16/2019 |
| 4DNESQV6Y4JL | contact | list-combined | 4.6E+08 | 1.86E+08 | 86015400 | 1.88E+08 | 7/16/2019 |
| 4DNESFM66XDL | contact | list-combined | 3.18E+08 | 1.44E+08 | 38102676 | 1.36E+08 | 7/16/2019 |
| 4DNESZUWCRVN | contact | list-combined | 1.24E+08 | 43713555 | 13865704 | 65997996 | 7/16/2019 |
| 4DNESCQRIZ7D | contact | list-combined | 3.67E+08 | 1.13E+08 | 36634927 | 2.18E+08 | 7/16/2019 |
| 4DNESLVTLWPX | contact | list-combined | 3.83E+08 | 87090094 | 37604772 | 2.59E+08 | 7/16/2019 |
| 4DNESF17LNZE | contact | list-combined | 6.02E+08 | 1.21E+08 | 83117695 | 3.98E+08 | 7/16/2019 |
| 4DNESSH9ICEW | contact | list-combined | 4.07E+08 | 42832515 | 36913681 | 3.28E+08 | 7/16/2019 |
| 4DNESC23ZYOF | contact | list-combined | 1.87E+08 | 77275861 | 21730571 | 87950217 | 7/16/2019 |
| 4DNES54GS5KI | contact | list-combined | 2.29E+08 | 92586821 | 22912944 | 1.13E+08 | 7/16/2019 |
| 4DNESPXSO8GB | contact | list-combined | 30309937 | 10084212 | 3493937 | 16731788 | 7/16/2019 |
| 4DNESHJVC7MP | contact | list-combined | 24260947 | 10069323 | 3211492 | 10980132 | 7/16/2019 |
| 4DNESMG7JML8 | contact | list-combined | 26557616 | 7187529 | 2945713 | 16424374 | 7/16/2019 |
| 4DNESYVC49SD | contact | list-combined | 31793184 | 3267321 | 3280687 | 25245176 | 7/16/2019 |
| 4DNEST7Y7S69 | contact | list-combined | 1.83E+08 | 30730514 | 19326779 | 1.33E+08 | 7/16/2019 |
| 4DNESFOADERB | contact | list-combined | 1.19E+08 | 54618975 | 52185862 | 12403776 | 9/10/2019 |
| 4DNESFI64TG3 | contact | list-combined | 1.31E+08 | 64055039 | 50035974 | 16538488 | 9/10/2019 |
| 4DNESU95RUNO | contact | list-combined | 1.09E+08 | 60862224 | 22611193 | 25902425 | 9/10/2019 |
| 4DNESPOC41XG | contact | list-combined | 1.09E+08 | 61490978 | 23253507 | 24375960 | 9/10/2019 |
| 4DNES9BTO2FB | contact | list-combined | 84627724 | 34287159 | 12166372 | 38174193 | 11/4/2019 |
| 4DNESVR7L225 | contact | list-combined | 73553715 | 39447923 | 9723846 | 24381946 | 11/4/2019 |
| 4DNESCJHJ477 | contact | list-combined | 69063444 | 37277597 | 9894992 | 21890855 | 11/4/2019 |
| 4DNESS4NRI4U | contact | list-combined | 54056677 | 29138384 | 7173891 | 17744402 | 11/4/2019 |
| 4DNESPLDAR9W | contact | list-combined | 56066981 | 28905603 | 8001049 | 19160329 | 11/4/2019 |
| 4DNESI74G82F | contact | list-combined | 66284431 | 34407786 | 9672447 | 22204198 | 11/4/2019 |
| 4DNESXSGHXDQ | contact | list-combined | 61341839 | 30843728 | 8288121 | 22209990 | 11/4/2019 |
| 4DNESAEUDBTF | contact | list-combined | 98026008 | 48107077 | 13600974 | 36317957 | 11/4/2019 |
| 4DNESGQ1XP78 | contact | list-combined | 99968456 | 47095351 | 14955898 | 37917207 | 11/4/2019 |
| 4DNESYJGWM4L | contact | list-combined | 54692690 | 28103097 | 7704805 | 18884788 | 11/4/2019 |
| 4DNES25QLF7A | contact | list-combined | 69870837 | 33019683 | 10731200 | 26119954 | 11/4/2019 |
| 4DNESDRC93UG | contact | list-combined | 70764650 | 31587088 | 11887913 | 27289649 | 11/4/2019 |
| 4DNESAM3IMUG | contact | list-combined | 42955346 | 19972996 | 6585265 | 16397085 | 11/4/2019 |
| 4DNESQ5LDJKC | contact | list-combined | 46324722 | 21461734 | 7761014 | 17101974 | 11/4/2019 |
| 4DNESDW7JTOW | contact | list-combined | 49635515 | 22870510 | 7645823 | 19119182 | 11/4/2019 |
| 4DNES1V3OHQH | contact | list-combined | 57101295 | 24850336 | 9057263 | 23193696 | 11/4/2019 |
| 4DNESALYYHNP | contact | list-combined | 67387872 | 30576380 | 10631075 | 26180417 | 11/4/2019 |
| 4DNESYK6LDN6 | contact | list-combined | 41331359 | 16694619 | 9203271 | 15433469 | 11/4/2019 |
| 4DNESYB59H2X | contact | list-combined | 51504851 | 30463740 | 11276438 | 9764673 | 11/4/2019 |
| 4DNESA2GN9N4 | contact | list-combined | 48599921 | 26771279 | 10100089 | 11728553 | 11/4/2019 |
| 4DNESFMZQ64I | contact | list-combined | 47432185 | 25523957 | 10256859 | 11651369 | 11/4/2019 |
| 4DNESL8HVDR5 | contact | list-combined | 55161932 | 28814687 | 11583457 | 14763788 | 11/4/2019 |
| 4DNES42T87S8 | contact | list-combined | 62472199 | 31890334 | 13078718 | 17503147 | 11/4/2019 |
| 4DNESDRECPY8 | contact | list-combined | 58922990 | 28741898 | 12282804 | 17898288 | 11/4/2019 |
| 4DNESITQCT9N | contact | list-combined | 53120647 | 25203982 | 12550239 | 15366426 | 11/4/2019 |
| 4DNESLBDF71X | contact | list-combined | 63944354 | 27203567 | 14721929 | 22018858 | 11/4/2019 |
| 4DNESNG6M4GI | contact | list-combined | 61852389 | 28384806 | 14909680 | 18557903 | 11/4/2019 |
| 4DNES1UQQCQC | contact | list-combined | 60005554 | 24674534 | 13606965 | 21724055 | 11/4/2019 |
| 4DNESXQ13LRW | contact | list-combined | 72262090 | 32063684 | 15678063 | 24520343 | 11/4/2019 |
| 4DNESW54C11P | contact | list-combined | 72543704 | 29653210 | 15874213 | 27016281 | 11/4/2019 |
| 4DNESOV38MUI | contact | list-combined | 74624833 | 33742066 | 17160097 | 23722670 | 11/4/2019 |
| 4DNESOUACCOP | contact | list-combined | 56665874 | 23622736 | 13486194 | 19556944 | 11/4/2019 |
| 4DNESJN4ZXIC | contact | list-combined | 74595647 | 32410216 | 18003058 | 24182373 | 11/4/2019 |
| 4DNESES8DB3N | contact | list-combined | 62623171 | 25359140 | 15310847 | 21953184 | 11/4/2019 |
| 4DNESFRB6NSI | contact | list-combined | 60128977 | 25380982 | 15300728 | 19447267 | 11/4/2019 |
| 4DNESO57HS3X | contact | list-combined | 59710055 | 23988716 | 14342513 | 21378826 | 11/4/2019 |
| 4DNESFKQBEV1 | contact | list-combined | 46222283 | 17129632 | 10442825 | 18649826 | 11/4/2019 |
| 4DNESPFYORMI | contact | list-combined | 33372434 | 17135810 | 7303204 | 8933420 | 11/4/2019 |
| 4DNES2AHLK4O | contact | list-combined | 33571600 | 15698988 | 7750515 | 10122097 | 11/4/2019 |
| 4DNES2R93GH1 | contact | list-combined | 49589536 | 23710175 | 11032235 | 14847126 | 11/4/2019 |
| 4DNES6VPZLD6 | contact | list-combined | 47877494 | 21791745 | 11310249 | 14775500 | 11/4/2019 |
| 4DNES8BXQHNL | contact | list-combined | 68558785 | 32926003 | 14279638 | 21353144 | 11/4/2019 |
| 4DNES3J3DFUS | contact | list-combined | 40449605 | 18390102 | 8855428 | 13204075 | 11/4/2019 |
| 4DNESXZV4GUI | contact | list-combined | 43731567 | 20882863 | 9610233 | 13238471 | 11/4/2019 |
| 4DNESAF3KM2R | contact | list-combined | 39869181 | 17464086 | 9094630 | 13310465 | 11/4/2019 |
| 4DNESZNNP25B | contact | list-combined | 57892128 | 26051763 | 11583865 | 20256500 | 11/4/2019 |
| 4DNESEX4VDTH | contact | list-combined | 46590765 | 21923960 | 11464260 | 13202545 | 11/4/2019 |
| 4DNES98NQ39I | contact | list-combined | 68275321 | 28837106 | 15486760 | 23951455 | 11/4/2019 |
| 4DNESMYI53QK | contact | list-combined | 37030856 | 14408243 | 9397758 | 13224855 | 11/4/2019 |
| 4DNES86MYL3E | contact | list-combined | 44688535 | 18701982 | 10649986 | 15336567 | 11/4/2019 |
| 4DNES33L8EEV | contact | list-combined | 48002002 | 17652644 | 11645418 | 18703940 | 11/4/2019 |
| 4DNESPWGWJYA | contact | list-combined | 47747039 | 16348909 | 12654029 | 18744101 | 11/4/2019 |
| 4DNESJ55821X | contact | list-combined | 44180679 | 16914062 | 11111826 | 16154791 | 11/4/2019 |
| 4DNESF7LJ88J | contact | list-combined | 55732796 | 20383704 | 13169110 | 22179982 | 11/4/2019 |
| 4DNESM7PB81R | contact | list-combined | 53618431 | 20314108 | 12157788 | 21146535 | 11/4/2019 |
| 4DNESCMQ9JOF | contact | list-combined | 31316228 | 11077310 | 6674332 | 13564586 | 11/4/2019 |
| 4DNESM14SDMG | contact | list-combined | 45377214 | 16061949 | 11483108 | 17832157 | 11/4/2019 |
| 4DNES5GB1X5P | contact | list-combined | 32261393 | 11522472 | 8178850 | 12560071 | 11/4/2019 |
| 4DNES2RQ6BDT | contact | list-combined | 31255257 | 10359201 | 8096660 | 12799396 | 11/4/2019 |
| 4DNES2R6PUEK | contact | list-combined | 2.93E+09 | 1.35E+09 | 6.63E+08 | 9.17E+08 | 3/14/2020 |
| 4DNES18BMU79 | contact | list-combined | 5.33E+08 | 2.57E+08 | 82209238 | 1.94E+08 | 2019-01 |
| 4DNESH4UTRNL | contact | list-combined | 2.02E+09 | 8.7E+08 | 3.71E+08 | 7.83E+08 | 2019-01 |
| 4DNESNYBDSLY | contact | list-combined | 1.2E+09 | 5.11E+08 | 2.55E+08 | 4.36E+08 | 2019-01 |
| 4DNES54YB6TQ | contact | list-combined | 1.59E+09 | 6.47E+08 | 3.56E+08 | 5.83E+08 | 2019-01 |
| 4DNESRE7AK5U | contact | list-combined | 2.96E+08 | 1.77E+08 | 61431613 | 57263487 | 2019-01 |
| 4DNES425UDGS | contact | list-combined | 6.19E+08 | 2.88E+08 | 1.21E+08 | 2.1E+08 | 2019-01 |
| 4DNESEPDL6KY | contact | list-combined | 5.58E+08 | 2.11E+08 | 1.43E+08 | 2.04E+08 | 2019-01 |
| 4DNESZW7OOTL | contact | list-combined | 1.88E+09 | 1.06E+09 | 3.21E+08 | 5.07E+08 | 2019-09 |
| 4DNESAL82BWY | contact | list-combined | 2.28E+09 | 1.29E+09 | 5.05E+08 | 4.83E+08 | 2019-09 |
| 4DNESMU2MA2G | contact | list-combined | 2.32E+09 | 1.28E+09 | 4.2E+08 | 6.17E+08 | 2019-09 |
| 4DNES1ONB8TD | contact | list-combined | 2.44E+09 | 1.18E+09 | 4.94E+08 | 7.64E+08 | 2019-09 |
| 4DNES8IIWFGK | contact | list-combined | 2.27E+09 | 1.23E+09 | 4.93E+08 | 5.4E+08 | 2019-09 |
| 4DNES1INHSG7 | contact | list-combined | 2.25E+09 | 1.3E+09 | 5.05E+08 | 4.43E+08 | 2019-09 |
| 4DNESWNF3Y23 | contact | list-combined | 4.77E+08 | 2.22E+08 | 1.73E+08 | 82014857 | 2019-12 |
| 4DNESWLWNWV8 | contact | list-combined | 4E+08 | 2.31E+08 | 1.28E+08 | 41988812 | 2019-12 |
| 4DNESL3AW546 | contact | list-combined | 4.49E+08 | 2.55E+08 | 1.54E+08 | 40210601 | 2019-12 |
| 4DNESTI1YC1H | contact | list-combined | 4.39E+08 | 2.79E+08 | 1.19E+08 | 40234238 | 2019-12 |
| 4DNESXX38FO6 | contact | list-combined | 4.14E+08 | 2.86E+08 | 1.07E+08 | 21173768 | 2019-12 |

Supplementary Table 2. **Sequencing depth of Hi-C maps in the 4DNucleome database used in our analysis.** A list of 4DNucleome datasets and their corresponding reads and publication date.

|  |  |  |  |  |  | Calculating first 4 eigenvectors | | | |
| --- | --- | --- | --- | --- | --- | --- | --- | --- | --- |
|  |  |  |  |  |  | Package: irlba | | Package: POSSUMM | |
| Format | Matrix Name | Group | Rows | Columns | Nonzeros | Time (h:m:s) | Mem. (GB) | Time (h:m:s) | Mem. (GB) |
| .mtx | webbase-1M | Williams | 1,000,005 | 1,000,005 | 3.11E+06 | 0:00:27 | 0.6 | 0:00:04 | 0.53 |
| .mtx | pdb1HYS | Williams | 36,417 | 36,417 | 2.19E+06 | 0:00:17 | 0.153 | 0:00:02 | 0.09 |
| .mtx | tx2010 | DIMACS10 | 914,231 | 914,231 | 2.23E+06 | 0:00:13 | 0.52 | 0:00:07 | 0.65 |
| .mtx | human_gene1 | Belcastro | 22,283 | 22,283 | 1.23E+07 | 0:00:41 | 0.55 | 0:00:05 | 0.16 |
| .mtx | cage14 | vanHeukelum | 1,505,785 | 1,505,785 | 2.71E+07 | 0:57:26 | 1.96 | 0:00:39 | 2.02 |
| .mtx | mawi  201512020330 | MAWI | 226,196,185 | 226,196,185 | 2.40E+08 | 1:18:24 | 125.50 | 0:02:31 | 48.29 |
| .mtx | GAP-urand | GAP | 134,217,728 | 134,217,728 | 2.15E+09 | - | - | 0:22:34 | 74.45 |
| .mtx | MOLIERE  2016 | Sybrant | 30,239,687 | 30,239,687 | 3.34E+09 | - | - | 0:34:00 | 55.19 |
| .mtx | GAP-twitter | GAP | 61,578,415 | 61,578,415 | 1.47E+09 | - | - | 0:17:48 | 51.37 |
|  |  |  |  |  |  |  |  |  |  |
| .hic | UltraRes-HiC-chr1_500bp | This Study | 498,501 | 498,501 | 8.42E+08 | - | - | 0:02:32 | 23.09 |
| .hic | UltraRes-HiC-INTER_500bp | This Study | 6,191,350 | 6,191,350 | 6.23E+09 | - | - | 0:39:19 | 77.65 |

Supplementary Table 3. **Time and Memory usage.** Time and memory usage comparison for irlba and POSSUMM calculating the first 4 eigenvectors of various matrices. Large matrices were obtained from <https://sparse.tamu.edu> and demonstrate the widespread utility of POSSUMM for PCA analysis. The descriptions of these matrices include - webbase-1M: web-connectivity data; pdb1HYS: protein data bank; tx2010: US census; humang_gene1: human gene network; cage14: DNA electrophoresis, 14 monomers in polymer; mawi: network traffic data; GAP_urand: benchmark matrix with random integer edge weights; MOLIERE: Automatic Biomedical Hypothesis Generation System; GAP-twitter: social newtwork topology.

| Computer Specs: | Ubuntu 18.04.3, Intel Xeon Gold, 6140 CPU @ 2.3 GHz | | | | | | | | | | |
| --- | --- | --- | --- | --- | --- | --- | --- | --- | --- | --- | --- |
| Bin Size (kb) | | 0.5 | 1 | 5 | 10 | 25 | 50 | 100 | 250 | 500 | 1000 |
| Time (minutes) | Dense | NA | NA | NA | NA | 321.65 | 34.7 | 3.623 | 0.2 | 0.042 | 0.02 |
|  | Sparse | 12.38 | 6.585 | 2.829 | 1.431 | 0.366 | 0.101 | 0.027 | 0.005 | 0.002 | 0.001 |
| Memory (mb) | Dense | NA | NA | NA | NA | 6945.6 | 2371 | 831.7 | 276 | 206.3 | 187 |
|  | Sparse | 13224 | 10098 | 4349 | 2221 | 572.1 | 157 | 42.4 | 8.1 | 4.21 | 2.9 |

Supplementary Table 4. **Compartment identification on sparse vs. dense matrices.** Time and memory usage of identifying compartments on sparse vs. dense matrices at various bin sizes.


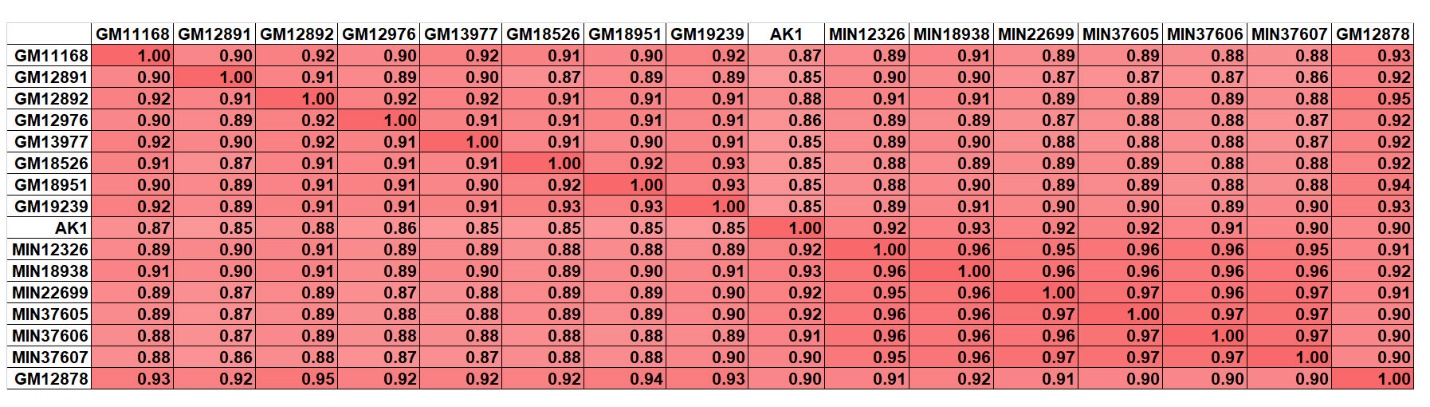


Supplementary Table 5. **Correlation between individual LCL maps.** HiCRep Stratum Adjusted Correlation Coefficient (SCC) pairwise reproducibility scores for each lymphoblastoid cell line were used to form the full dataset.


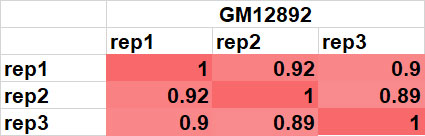


Supplementary Table 6. **Correlation between individual replicates.** HiCRep Stratum Adjusted Correlation Coefficient (SCC) pairwise reproducibility scores for each replicate within a single cell line.

| Hi-C used for quality comparison |
| --- |
| GM12878 (GSE63525) |
| HCT-116 (GSE104334) |
| K562 (4DNESU95RUNO) |
| HMEC (GSE63525) |
| HUVEC (GSE63525) |
| HELA (GSE63525) |
| IMR90 (GSE63525) |
| K562 (GSE63525) |
| KBM7 (GSE63525) |
| NHEK (GSE63525) |
| HAP1 (GSE95014) |
| H9 (GSE105028) |

Supplementary Table 7. **Hi-C datasets used for quality metric comparisons.** A list of the maps used for quality comparisons.
