# Supplementary material for: Chromatin alternates between A and B compartments at kilobase scale for subgenic organization
Source: Nat Commun. 2023 Jun 6;14:3303. doi: 10.1038/s41467-023-38429-1 (PMC10244318; doi:10.1038/s41467-023-38429-1)
Supplement: Supplementary file 3 — Reporting Summary [file 41467_2023_38429_MOESM3_ESM.pdf]

nature portfolio

Corresponding author(s): M Jordan Rowley

Last updated by author(s): March 31, 2023

Reporting Summary

Nature Portfolio wishes to improve the reproducibility of the work that we publish. This form provides structure for consistency and transparency in reporting. For further information on Nature Portfolio policies, see our Editorial Policies and the Editorial Policy Checklist.

Statistics

For all statistical analyses, confirm that the following items are present in the figure legend, table legend, main text, or Methods section.

n/a	Confirmed	
☐	☒	The exact sample size (*n*) for each experimental group/condition, given as a discrete number and unit of measurement
☐	☒	A statement on whether measurements were taken from distinct samples or whether the same sample was measured repeatedly
☐	☒	The statistical test(s) used AND whether they are one- or two-sided<br>*Only common tests should be described solely by name; describe more complex techniques in the Methods section.*
☐	☒	A description of all covariates tested
☐	☒	A description of any assumptions or corrections, such as tests of normality and adjustment for multiple comparisons
☐	☒	A full description of the statistical parameters including central tendency (e.g. means) or other basic estimates (e.g. regression coefficient) AND variation (e.g. standard deviation) or associated estimates of uncertainty (e.g. confidence intervals)
☐	☒	For null hypothesis testing, the test statistic (e.g. *F*, *t*, *r*) with confidence intervals, effect sizes, degrees of freedom and *P* value noted<br>*Give P values as exact values whenever suitable.*
☐	☒	For Bayesian analysis, information on the choice of priors and Markov chain Monte Carlo settings
☐	☒	For hierarchical and complex designs, identification of the appropriate level for tests and full reporting of outcomes
☐	☒	Estimates of effect sizes (e.g. Cohen's *d*, Pearson's *r*), indicating how they were calculated

Our web collection on statistics for biologists contains articles on many of the points above.

Software and code

Policy information about availability of computer code

Data collection	Illumina HiSeq 4000 software
Data analysis	JuicerTools v1.14.08; HiCRep v1.12.2; avocado v0.1.0; scikit-learn v1.0.2; statsmodels v0.13.2; SIP v1.4, SIPMeta v1.3; OnTAD v1.4; Fit-Hi-C v2.0.7; R package irlba v2.3.5; CScoreTool v1.1; Calder v1.0; HiCNoiseMeasurer v1.0(custom) - https://github.com/JRowleyLab/HiCNoiseMeasurer v1.0; HiCSampler (custom) - https://github.com/JRowleyLab/HiCSampler; POSSUMM v 1.0 (custom) - https://github.com/aidenlab/EigenVector; MiChroM (custom) - https://github.com/DiPierroLab/;

For manuscripts utilizing custom algorithms or software that are central to the research but not yet described in published literature, software must be made available to editors and reviewers. We strongly encourage code deposition in a community repository (e.g. GitHub). See the Nature Portfolio guidelines for submitting code & software for further information.

Data

Policy information about availability of data

All manuscripts must include a data availability statement. This statement should provide the following information, where applicable:

- Accession codes, unique identifiers, or web links for publicly available datasets
- A description of any restrictions on data availability
- For clinical datasets or third party data, please ensure that the statement adheres to our policy

The human genome 19 (hg19) assembly is available from the NCBI accession GCF_000001405.13 (https://www.ncbi.nlm.nih.gov/assembly/GCF_000001405.13/).

The Hi-C data from public LCLs generated in this study have been deposited in the ENCODE database under accession codes: ENCSR261EVH for GM13977 (https://www.encodeproject.org/experiments/ENCSR261EVH/), ENCSR196MPD for GM11168 (https://www.encodeproject.org/experiments/ENCSR196MPD/), ENCSR118FFR for GM18951 (https://www.encodeproject.org/experiments/ENCSR118FFR/), ENCSR634FNY for GM13976 (https://www.encodeproject.org/experiments/ENCSR634FNY/), ENCSR410MDC for GM12878 (https://www.encodeproject.org/experiments/ENCSR410MDC/), ENCSR508EMN for AK1 (https://www.encodeproject.org/experiments/ENCSR508EMN/), ENCSR859YSL for GM12891 https://www.encodeproject.org/experiments/ENCSR859YSL/), ENCSR075VWI for GM12892 https://www.encodeproject.org/experiments/ENCSR075VWI/), ENCSR693CIM for GM18526 (https://www.encodeproject.org/experiments/ENCSR693CIM/), and ENCSR264SMC for GM19239 (https://www.encodeproject.org/experiments/ENCSR693CIM/). The combined signal matrix is browsable using juicebox.js at https://tinyurl.com/yf2losh7. The previously published data used in this study are available in the ENCODE database under accessions ENCSR977QPF (https://www.encodeproject.org/reference-epigenomes/ENCSR977QPF/) for histone modifications and DNase-seq, ENCSR447YYN (https://www.encodeproject.org/reference-epigenomes/ENCSR447YYN/) for histone marks and RNAPIIser5ph, ENCSR000DZK (https://www.encodeproject.org/experiments/ENCSR000DZK/) for RNAPIISer2ph, and from the Gene Expression Omnibus (GEO) unders accession GSM1480326 (https://www.ncbi.nlm.nih.gov/geo/query/acc.cgi?acc=GSE60454) for GRO-seq, GSE123552 (https://www.ncbi.nlm.nih.gov/geo/query/acc.cgi?acc=GSE123552) for PGP1f Hi-C, GSE125595 (https://www.ncbi.nlm.nih.gov/geo/query/acc.cgi?acc=GSE125595) for Hi-C in ZF mutants , GSE101498 (https://www.ncbi.nlm.nih.gov/geo/query/acc.cgi?acc=GSE101498) for H3K27ac HiChIP, GSE132640 (https://www.ncbi.nlm.nih.gov/geo/query/acc.cgi?acc=GSE132640) for Hi-C in C. elegans, GSE80701 (https://www.ncbi.nlm.nih.gov/geo/query/acc.cgi?acc=GSE80701) for Hi-C in D. melanogaster cells, and from the Roadmap Epigenomics Project (https://egg2.wustl.edu/roadmap/data/byFileType/chromhmmSegmentations/ChmmModels/coreMarks/jointModel/final/E116_15_coreMarks_dense.bed.gz) for chromHMM states. Chromatin 3D structures are deposited in the Nucleome Data Bank.

Human research participants

Policy information about studies involving human research participants and Sex and Gender in Research.

Reporting on sex and gender	This study combines samples from both male and female donors. Male lines include GM12891, GM11168, GM19239, AK1. Female lines include GM12892, GM18951, GM18526, GM13976, GM13977, GM12878. It was necessary to combine to obtain the resolution, and therefore we do not compare and account for sex in this study. However, we provide the individual correlations between individual maps along with the gender of each, see Source Data. The individual maps are also available through the relevant accessions listed in Data Availability.
Population characteristics	Samples were lymphoblastoid cell lines with unspecified age in the current Cellosaurus database and were derived from peripheral blood with ancestry from China, Northern and Western Europe, Japan, Korea, Nigeria.
Recruitment	Data was mostly obtained from publicly available cell lines. Secondary analysis was performed on samples that were recruited under the original IRB protocol #2013P000323.
Ethics oversight	Institutional Review Board of MassGeneral Brigham. Secondary analysis usder MGB IRB Protocol #2013P000323

Note that full information on the approval of the study protocol must also be provided in the manuscript.

Field-specific reporting

Please select the one below that is the best fit for your research. If you are not sure, read the appropriate sections before making your selection.

☒ Life sciences ☐ Behavioural & social sciences ☐ Ecological, evolutionary & environmental sciences

For a reference copy of the document with all sections, see nature.com/documents/nr-reporting-summary-flat.pdf

Life sciences study design

All studies must disclose on these points even when the disclosure is negative.

Sample size	Genome-wide Hi-C from cell lines from 17 individuals that comprise 150 individual Hi-C experiments. Sample size statistics were not applicable to this study as we do not seek to compare individuals, but rather use these samples to generate an ultra-deep map of an lymphoblastoid cell. The rationale to combine these maps is derived from the statistical testing of similarity via stratum adjusted correlation coefficient which showed high similarity among individuals and between replicates.
Data exclusions	No dataset was excluded from the analysis.
Replication	Each individual cell line was replicated at least 3 times. HiCRep Stratum Adjusted Correlation Coefficient (SCC) was used to measure pairwise reproducibility between individual replicates as well as between individuals.
Randomization	This study seeks to uncover new aspects of chromatin organization by ultra-deep sequencing. Randomized groupings of samples would have less depth than the fully combined map and be uncomparable at the resolution we use. We demonstrate the issue of comparing high depth to low depth data within supplementary figures 1 and 2. Because of the observed reproducibility between individual maps by stratum adjusted correlation coefficient, Hi-C maps were merged to create the ultra-deep map of chromatin interactions and samples were not split into distinct groupings.
Blinding	Blinding was irrelevant as group comparison was not part of the study.

Reporting for specific materials, systems and methods

We require information from authors about some types of materials, experimental systems and methods used in many studies. Here, indicate whether each material, system or method listed is relevant to your study. If you are not sure if a list item applies to your research, read the appropriate section before selecting a response.

Materials & experimental systems

n/a	Involved in the study
☒	☐ Antibodies
☐	☒ Eukaryotic cell lines
☒	☐ Palaeontology and archaeology
☒	☐ Animals and other organisms
☒	☐ Clinical data
☒	☐ Dual use research of concern

Methods

n/a	Involved in the study
☒	☐ ChIP-seq
☒	☐ Flow cytometry
☒	☐ MRI-based neuroimaging

Eukaryotic cell lines

Policy information about cell lines and Sex and Gender in Research

Cell line source(s)	Cell lines are obtained via ENCODE from the Coriell Institute for Medical Research, including GM13977, GM11168, GM18951, GM13976, GM12878, GM12891, GM12892, GM18526, and GM19239.
Authentication	Cells were authenticated and cultured according to the ENCODE guidelines including Nucleoside Phosphorylase, Glucose-6-Phosphate Dehydrogenase, and Lactate Dehydrogenase Isoenzyme electrophoresis.
Mycoplasma contamination	Lymphoblastoid cell lines obtained from the Coriell Cell Repositories were established by Epstein-Barr virus transformation of peripheral blood mononuclear cells using phytohemagluttinin as a mitogen. All cells lines are free of bacterial, fungal, or mycoplasma contamination.
Commonly misidentified lines (See ICLAC register)	No commonly misidentified cell lines were used in the study.
